# Mendelian randomisation study of height and body mass index as modifiers of ovarian cancer risk in 22,588 *BRCA1* and *BRCA2* mutation carriers

**DOI:** 10.1038/s41416-019-0492-8

**Published:** 2019-06-19

**Authors:** Frank Qian, Matti A. Rookus, Goska Leslie, Harvey A. Risch, Mark H. Greene, Cora M. Aalfs, Muriel A. Adank, Julian Adlard, Bjarni A. Agnarsson, Munaza Ahmed, Kristiina Aittomäki, Irene L. Andrulis, Norbert Arnold, Banu K. Arun, Margreet G. E. M. Ausems, Jacopo Azzollini, Daniel Barrowdale, Julian Barwell, Javier Benitez, Katarzyna Białkowska, Valérie Bonadona, Julika Borde, Ake Borg, Angela R. Bradbury, Joan Brunet, Saundra S. Buys, Trinidad Caldés, Maria A. Caligo, Ian Campbell, Jonathan Carter, Jocelyne Chiquette, Wendy K. Chung, Kathleen B. M. Claes, J. Margriet Collée, Marie-Agnès Collonge-Rame, Fergus J. Couch, Mary B. Daly, Capucine Delnatte, Orland Diez, Susan M. Domchek, Cecilia M. Dorfling, Jacqueline Eason, Douglas F. Easton, Ros Eeles, Christoph Engel, D. Gareth Evans, Laurence Faivre, Lidia Feliubadaló, Lenka Foretova, Eitan Friedman, Debra Frost, Patricia A. Ganz, Judy Garber, Vanesa Garcia-Barberan, Andrea Gehrig, Gord Glendon, Andrew K. Godwin, Encarna B. Gómez Garcia, Ute Hamann, Jan Hauke, John L. Hopper, Peter J. Hulick, Evgeny N. Imyanitov, Claudine Isaacs, Louise Izatt, Anna Jakubowska, Ramunas Janavicius, Esther M. John, Beth Y. Karlan, Carolien M. Kets, Yael Laitman, Conxi Lázaro, Dominique Leroux, Jenny Lester, Fabienne Lesueur, Jennifer T. Loud, Jan Lubiński, Alicja Łukomska, Lesley McGuffog, Noura Mebirouk, Hanne E. J. Meijers-Heijboer, Alfons Meindl, Austin Miller, Marco Montagna, Thea M. Mooij, Emmanuelle Mouret-Fourme, Katherine L. Nathanson, Bita Nehoray, Susan L. Neuhausen, Heli Nevanlinna, Finn C. Nielsen, Kenneth Offit, Edith Olah, Kai-ren Ong, Jan C. Oosterwijk, Laura Ottini, Michael T. Parsons, Paolo Peterlongo, Georg Pfeiler, Nisha Pradhan, Paolo Radice, Susan J. Ramus, Johanna Rantala, Gad Rennert, Mark Robson, Gustavo C. Rodriguez, Ritu Salani, Maren T. Scheuner, Rita K. Schmutzler, Payal D. Shah, Lucy E. Side, Jacques Simard, Christian F. Singer, Doris Steinemann, Dominique Stoppa-Lyonnet, Yen Yen Tan, Manuel R. Teixeira, Mary Beth Terry, Mads Thomassen, Marc Tischkowitz, Silvia Tognazzo, Amanda E. Toland, Nadine Tung, Christi J. van Asperen, Klaartje van Engelen, Elizabeth J. van Rensburg, Laurence Venat-Bouvet, Jeroen Vierstraete, Gabriel Wagner, Lisa Walker, Jeffrey N. Weitzel, Drakoulis Yannoukakos, KConFab Investigators, HEBON Investigators, GEMO Study Collaborators, EMBRACE Collaborators, Antonis C. Antoniou, David E. Goldgar, Olufunmilayo I. Olopade, Georgia Chenevix-Trench, Timothy R. Rebbeck, Dezheng Huo, on behalf of CIMBA

**Affiliations:** 1 Department of Medicine, University of Chicago, Chicago, IL USA; 2 Department of Epidemiology, The Netherlands Cancer Institute, Amsterdam, The Netherlands; 3 Centre for Cancer Genetic Epidemiology, Department of Public Health and Primary Care, University of Cambridge, Cambridge, UK; 4 Chronic Disease Epidemiology, Yale School of Public Health, New Haven, CT USA; 5 Clinical Genetics Branch, Division of Cancer Epidemiology and Genetics, National Cancer Institute, Bethesda, MD USA; 6 Department of Clinical Genetics, Amsterdam UMC, Location AMC, Amsterdam, The Netherlands; 7 Family Cancer Clinic, The Netherlands Cancer Institute - Antoni van Leeuwenhoek Hospital, Amsterdam, The Netherlands; 8 Yorkshire Regional Genetics Service, Chapel Allerton Hospital, Leeds, UK; 9 Department of Pathology, Landspitali University Hospital, Reykjavik, Iceland; 10 School of Medicine, University of Iceland, Reykjavik, Iceland; 11 North East Thames Regional Genetics Service, Great Ormond Street Hospital, London, UK; 12 Department of Clinical Genetics, Helsinki University Hospital, University of Helsinki, Helsinki, Finland; 13 Fred A. Litwin Center for Cancer Genetics, Lunenfeld-Tanenbaum Research Institute of Mount Sinai Hospital, Toronto, ON Canada; 14 Department of Molecular Genetics, University of Toronto, Toronto, ON Canada; 15 Department of Gynaecology and Obstetrics, University Hospital of Schleswig-Holstein, Campus Kiel, Christian-Albrechts University Kiel, Kiel, Germany; 16 Institute of Clinical Molecular Biology, University Hospital of Schleswig-Holstein, Campus Kiel, Christian-Albrechts University Kiel, Kiel, Germany; 17 Department of Breast Medical Oncology, University of Texas MD Anderson Cancer Center, Houston, TX USA; 18 Division Laboratories, Pharmacy and Biomedical Genetics, Department of Medical Genetics, University Medical Center Utrecht, Utrecht, The Netherlands; 19 Unit of Medical Genetics, Department of Medical Oncology and Hematology, Fondazione IRCCS Istituto Nazionale dei Tumori di Milano, Milan, Italy; 20 Leicestershire Clinical Genetics Service, University Hospitals of Leicester NHS Trust, Leicester, UK; 21 Human Cancer Genetics Programme, Spanish National Cancer Research Centre (CNIO), Madrid, Spain; 22 Biomedical Network on Rare Diseases (CIBERER), Madrid, Spain; 23 Department of Genetics and Pathology, Pomeranian Medical University, Szczecin, Poland; 24 Unité de Prévention et d’Epidémiologie Génétique, Centre Léon Bérard, Lyon, France; 25 Center for Integrated Oncology (CIO), University Hospital of Cologne, Cologne, Germany; 26 Center for Molecular Medicine Cologne (CMMC), University of Cologne, Cologne, Germany; 27 Center for Hereditary Breast and Ovarian Cancer, University Hospital of Cologne, Cologne, Germany; 28 Department of Oncology, Lund University and Skåne University Hospital, Lund, Sweden; 29 Department of Medicine, Abramson Cancer Center, Perelman School of Medicine at the University of Pennsylvania, Philadelphia, PA USA; 30 Genetic Counseling Unit, Hereditary Cancer Program, IDIBGI (Institut d’Investigació Biomèdica de Girona), Catalan Institute of Oncology, CIBERONC, Girona, Spain; 31 Department of Medicine, Huntsman Cancer Institute, Salt Lake City, UT USA; 32 Medical Oncology Department, Hospital Clínico San Carlos, Instituto de Investigación Sanitaria San Carlos (IdISSC), Centro Investigación Biomédica en Red de Cáncer (CIBERONC), Madrid, Spain; 33 Section of Molecular Genetics, Dept. of Laboratory Medicine, University Hospital of Pisa, Pisa, Italy; 34 Peter MacCallum Cancer Center, Melbourne, Victoria, Australia; 35 Sir Peter MacCallum Department of Oncology, The University of Melbourne, Melbourne, VIC Australia; 36 Department of Gynaecological Oncology, Chris O’Brien Lifehouse and The University of Sydney, Camperdown, NSW Australia; 37 CRCHU de Québec- oncologie, Centre des maladies du sein Deschênes-Fabia, Hôpital du Saint-Sacrement, Québec, QC Canada; 38 Departments of Pediatrics and Medicine, Columbia University, New York, NY USA; 39 Centre for Medical Genetics, Ghent University, Gent, Belgium; 40 Department of Clinical Genetics, Erasmus University Medical Center, Rotterdam, The Netherlands; 41 Service de Génétique, CHU de Besançon, Besançon, France; 42 Department of Laboratory Medicine and Pathology, Mayo Clinic, Rochester, MN USA; 43 Department of Clinical Genetics, Fox Chase Cancer Center, Philadelphia, PA USA; 44 Unité d’Oncogénétique, ICO-Centre René Gauducheau, Saint Herblain, France; 45 Oncogenetics Group, Clinical and Molecular Genetics Area, Vall d’Hebron Institute of Oncology (VHIO), University Hospital Vall d’Hebron, Barcelona, Spain; 46 Department of Genetics, University of Pretoria, Arcadia, South Africa; 47 Nottingham Clinical Genetics Service, Nottingham University Hospitals NHS Trust, Nottingham, UK; 48 Centre for Cancer Genetic Epidemiology, Department of Oncology, University of Cambridge, Cambridge, UK; 49 Oncogenetics Team, The Institute of Cancer Research and Royal Marsden NHS Foundation Trust, London, UK; 50 Institute for Medical Informatics, Statistics and Epidemiology, University of Leipzig, Leipzig, Germany; 51 Division of Evolution and Genomic Medicine, School of Biological Sciences, Faculty of Biology, Medicine and Health, University of Manchester, Manchester Academic Health Science Centre, Manchester, UK; 52 Manchester Centre for Genomic Medicine, St Mary’s Hospital, Central Manchester University Hospitals NHS Foundation Trust, Manchester Academic Health Science Centre, Manchester, UK; 53 Unité d’oncogénétique, Centre de Lutte Contre le Cancer, Centre Georges-François Leclerc, Dijon, France; 54 Centre de Génétique, CHU Dijon, Dijon, France; 55 Molecular Diagnostic Unit, Hereditary Cancer Program, ICO-IDIBELL (Bellvitge Biomedical Research Institute, Catalan Institute of Oncology), CIBERONC, Barcelona, Spain; 56 Department of Cancer Epidemiology and Genetics, Masaryk Memorial Cancer Institute, Brno, Czech Republic; 57 The Susanne Levy Gertner Oncogenetics Unit, Chaim Sheba Medical Center, Ramat Gan, Israel; 58 Sackler Faculty of Medicine, Tel Aviv University, Ramat Aviv, Israel; 59 Schools of Medicine and Public Health, Division of Cancer Prevention & Control Research, Jonsson Comprehensive Cancer Centre, UCLA, Los Angeles, CA USA; 60 Cancer Risk and Prevention Clinic, Dana-Farber Cancer Institute, Boston, MA USA; 61 Centre of Familial Breast and Ovarian Cancer, Department of Medical Genetics, Institute of Human Genetics, University Würzburg, Würzburg, Germany; 62 Department of Pathology and Laboratory Medicine, Kansas University Medical Center, Kansas City, KS USA; 63 Department of Clinical Genetics and GROW, School for Oncology and Developmental Biology, Maastricht University Medical Center, Maastricht, The Netherlands; 64 Molecular Genetics of Breast Cancer, German Cancer Research Center (DKFZ), Heidelberg, Germany; 65 Centre for Epidemiology and Biostatistics, Melbourne School of Population and Global Health, The University of Melbourne, Melbourne, VIC Australia; 66 Center for Medical Genetics, NorthShore University HealthSystem, Evanston, IL USA; 67 The University of Chicago Pritzker School of Medicine, Chicago, IL USA; 68 N.N. Petrov Institute of Oncology, St. Petersburg, Russia; 69 Lombardi Comprehensive Cancer Center, Georgetown University, Washington, DC USA; 70 Clinical Genetics, Guy’s and St Thomas’ NHS Foundation Trust, London, UK; 71 Independent Laboratory of Molecular Biology and Genetic Diagnostics, Pomeranian Medical University, Szczecin, Poland; 72 Hematology, oncology and transfusion medicine center, Dept. of Molecular and Regenerative Medicine, Vilnius University Hospital Santariskiu Clinics, Vilnius, Lithuania; 73 State Research Institute, Innovative Medicine Center, Vilnius, CA Lithuania; 74 Department of Medicine, Division of Oncology, and Stanford Cancer Institute, Stanford University School of Medicine, Stanford, CA USA; 75 Women’s Cancer Program at the Samuel Oschin Comprehensive Cancer Institute, Cedars-Sinai Medical Center, Los Angeles, CA USA; 76 Department of Human Genetics, Radboud University Medical Center, Nijmegen, The Netherlands; 77 Département de Génétique, CHU de Grenoble, Grenoble, France; 78 Genetic Epidemiology of Cancer team, Inserm U900, Paris, France; 79 Institut Curie, Paris, France; 80 Mines ParisTech, Fontainebleau, France; 81 Department of Clinical Genetics, Amsterdam UMC, Location VU University Medical Center, Amsterdam, The Netherlands; 82 Department of Gynecology and Obstetrics, Ludwig Maximilian University of Munich, Munich, Germany; 83 NRG Oncology, Statistics and Data Management Center, Roswell Park Cancer Institute, Buffalo, NY USA; 84 Immunology and Molecular Oncology Unit, Veneto Institute of Oncology IOV - IRCCS, Padua, Italy; 85 Service de Génétique, Institut Curie, Paris, France; 86 Clinical Cancer Genomics, City of Hope, Duarte, CA USA; 87 Department of Population Sciences, Beckman Research Institute of City of Hope, Duarte, CA USA; 88 Department of Obstetrics and Gynecology, Helsinki University Hospital, University of Helsinki, Helsinki, Finland; 89 Center for Genomic Medicine, Rigshospitalet, Copenhagen University Hospital, Copenhagen, Denmark; 90 Clinical Genetics Research Lab, Department of Cancer Biology and Genetics, Memorial Sloan-Kettering Cancer Center, New York, NY USA; 91 Clinical Genetics Service, Department of Medicine, Memorial Sloan-Kettering Cancer Center, New York, NY USA; 92 Department of Molecular Genetics, National Institute of Oncology, Budapest, Hungary; 93 West Midlands Regional Genetics Service, Birmingham Women’s Hospital Healthcare NHS Trust, Birmingham, UK; 94 Department of Genetics, University Medical Center Groningen, University Groningen, Groningen, The Netherlands; 95 Department of Molecular Medicine, University La Sapienza, Rome, Italy; 96 Department of Genetics and Computational Biology, QIMR Berghofer Medical Research Institute, Brisbane, QLD Australia; 97 Genome Diagnostics Program, IFOM - the FIRC (Italian Foundation for Cancer Research) Institute of Molecular Oncology, Milan, Italy; 98 Department of OB/GYN and Comprehensive Cancer Center, Medical University of Vienna, Vienna, Austria; 99 Unit of Molecular Bases of Genetic Risk and Genetic Testing, Department of Research, Fondazione IRCCS Istituto Nazionale dei Tumori (INT), Milan, Italy; 100 School of Women’s and Children’s Health, Faculty of Medicine, University of NSW Sydney, Sydney, NSW Australia; 101 The Kinghorn Cancer Centre, Garvan Institute of Medical Research, Sydney, NSW Australia; 102 Clinical Genetics, Karolinska Institutet, Stockholm, Sweden; 103 Clalit National Cancer Control Center, Carmel Medical Center and Technion Faculty of Medicine, Haifa, Israel; 104 Division of Gynecologic Oncology, NorthShore University HealthSystem, University of Chicago, Evanston, IL USA; 105 Wexner Medical Center, The Ohio State University, Columbus, OH USA; 106 Cancer Genetics and Prevention Program, University of California San Francisco, San Francisco, CA USA; 107 Wessex Clinical Genetics Service, University Hospitals Southampton NHS Trust, Southampton, UK; 108 Genomics Center, Centre Hospitalier Universitaire de Québec – Université Laval, Research Center, Québec City, QC Canada; 109 Institute of Cell and Molecular Pathology, Hannover Medical School, Hannover, Germany; 110 Department of Tumour Biology, INSERM U830, Paris, France; 111 Université Paris Descartes, Paris, France; 112 Department of Genetics, Portuguese Oncology Institute, Porto, Portugal; 113 Biomedical Sciences Institute (ICBAS), University of Porto, Porto, Portugal; 114 Department of Epidemiology, Mailman School of Public Health, Columbia University, New York, NY USA; 115 Department of Clinical Genetics, Odense University Hospital, Odence C, Denmark; 116 Program in Cancer Genetics, Departments of Human Genetics and Oncology, McGill University, Montréal, QC Canada; 117 Department of Medical Genetics, University of Cambridge, Cambridge, UK; 118 Department of Cancer Biology and Genetics, The Ohio State University, Columbus, OH USA; 119 Department of Medical Oncology, Beth Israel Deaconess Medical Center, Boston, MA USA; 120 Department of Clinical Genetics, Leiden University Medical Center, Leiden, The Netherlands; 121 Department of Clinical Genetics, Amsterdam UMC, Vrije Universiteit Amsterdam, Amsterdam, The Netherlands; 122 Department of Medical Oncology, CHU Dupuytren, Limoges, France; 123 Oxford Regional Genetics Service, Churchill Hospital, Oxford, UK; 124 Molecular Diagnostics Laboratory, INRASTES, National Centre for Scientific Research ‘Demokritos’, Athens, Greece; 125 The Hereditary Breast and Ovarian Cancer Research Group Netherlands (HEBON), Coordinating Center: The Netherlands Cancer Institute, Amsterdam, The Netherlands; 126 Department of Dermatology, Huntsman Cancer Institute, University of Utah School of Medicine, Salt Lake City, UT USA; 127 Center for Clinical Cancer Genetics, The University of Chicago, Chicago, IL USA; 128 Harvard T.H. Chan School of Public Health, Boston, MA USA; 129 Dana-Farber Cancer Institute, Boston, MA USA; 130 Department of Public Health Sciences, University of Chicago, Chicago, IL USA

**Keywords:** Risk factors, Cancer epidemiology, Epidemiology

## Abstract

**Background:**

Height and body mass index (BMI) are associated with higher ovarian cancer risk in the general population, but whether such associations exist among *BRCA1/2* mutation carriers is unknown.

**Methods:**

We applied a Mendelian randomisation approach to examine height/BMI with ovarian cancer risk using the Consortium of Investigators for the Modifiers of *BRCA1/2* (CIMBA) data set, comprising 14,676 *BRCA1* and 7912 *BRCA2* mutation carriers, with 2923 ovarian cancer cases. We created a height genetic score (height-GS) using 586 height-associated variants and a BMI genetic score (BMI-GS) using 93 BMI-associated variants. Associations were assessed using weighted Cox models.

**Results:**

Observed height was not associated with ovarian cancer risk (hazard ratio [HR]: 1.07 per 10-cm increase in height, 95% confidence interval [CI]: 0.94–1.23). Height-GS showed similar results (HR = 1.02, 95% CI: 0.85–1.23). Higher BMI was significantly associated with increased risk in premenopausal women with HR = 1.25 (95% CI: 1.06–1.48) and HR = 1.59 (95% CI: 1.08–2.33) per 5-kg/m^2^ increase in observed and genetically determined BMI, respectively. No association was found for postmenopausal women. Interaction between menopausal status and BMI was significant (*P*_interaction_ < 0.05).

**Conclusion:**

Our observation of a positive association between BMI and ovarian cancer risk in premenopausal *BRCA1/2* mutation carriers is consistent with findings in the general population.

## Background

Ovarian cancer is the fifth leading cause of cancer deaths in US women, due to its typically advanced stage at presentation.^1,2^ Furthermore, unlike breast or colorectal cancer, there is no proven screening method for ovarian cancer to identify early disease and initiate treatment to improve survival.^3,4^ Family history, oral contraceptive use, parity, body mass index (BMI), and genetic variants are potentially useful in estimating lifetime risk.^1^ In particular, inherited *BRCA1* and *BRCA2* mutations are associated with increased lifetime risk of ovarian cancer and account for ~10–15% of overall disease incidence.^5–7^ However, among mutation carriers, age at diagnosis is variable. Penetrance of *BRCA1/2* mutations is likely modified by other genetic variants and lifestyle or reproductive factors.^8,9^ Investigation of these factors could aid in implementation of strategies to reduce ovarian cancer risk among mutation carriers.

Both height and BMI are quantitative traits with substantial genetic bases. In recent genome-wide association studies (GWAS), numerous genetic variants were found to be associated with these traits.^10,11^ In the general population, both height and BMI appear to be positively but inconsistently associated with risk of ovarian cancer.^12–14^ Previous studies also showed that the association between BMI and ovarian cancer was stronger in premenopausal women.^12,15,16^ Because of differences in age at onset and tumour histology/grade, risk factors for ovarian cancer might be different for *BRCA1/2* mutation carriers than women in the general population.^17^ Only one case–control study, with 469 ovarian cancer cases, has examined anthropometric measurements in *BRCA1/2* mutation carriers and found that neither height nor BMI were related to ovarian cancer risk.^18^ Larger, adequately powered studies are needed to assess whether a relationship between either height or BMI and ovarian cancer risk exists for *BRCA1/2* mutation carriers, and whether the direction of association is concordant with that in the general population.

Mendelian randomisation (MR) methods use genetic markers associated with a trait as an instrumental variable (IV) to assess their potential relationship with a disease outcome.^19–21^ Compared to traditional epidemiologic approaches, MR can reduce biases such as reverse causation and residual confounding, which can interfere with causal interpretations. However, the MR approach requires that the genetic variants are associated with the exposure, the variants are not or only weakly associated with confounding factors in the causal pathway, and the variants only affect disease risk through the exposure (i.e. absence of pleiotropic effects).^20,21^ To the degree that these assumptions are met, the MR approach can strengthen the evidence for a causal relationship between exposure and disease.

Herein, using traditional epidemiologic and MR methods, we conducted analyses of height and BMI and their association with ovarian cancer risk in the Consortium of Investigators for the Modifiers of *BRCA1/2* (CIMBA) with 22,588 participants. We examined heterogeneity of these associations with respect to the mutation carried (*BRCA1* vs *BRCA2*), menopausal status, tumour histology, and tumour grade.

## Methods

Characteristics of the CIMBA consortium and information on specific genotyping protocols are provided in Supplementary Methods and were described previously.^22–24^

### Selection of genetic variants

From the latest publications of the Genetic Investigation of Anthropometric Traits, we identified single-nucleotide polymorphisms (SNPs) associated with height or BMI at genome-wide significance level (*P* < 5 × 10^−8^).^11,25^ SNPs with low imputation quality (<0.5) were excluded, leaving 586 SNPs for height and 93 for BMI. Supplementary Tables 1 and 2 provide additional details on these SNPs.

### Statistical analysis

Calculation of the height and BMI genetic scores (GS) was described in detail previously.^24^ Briefly, we calculated the weighted sums of all of the height- and BMI-associated variants under additive models, which do not include interactions between variants. Namely, we used the formulas: ${\mathrm{Height - GS}} = \mathop {\sum}\nolimits_{i = 1}^{586} {\beta _{XGi}{\mathrm{SNP}}_i}$ and ${\mathrm{BMI - GS}} = \mathop {\sum}\nolimits_{i = 1}^{93} {\beta _{XGi}{\mathrm{SNP}}}$, where *β*_*XGi*_ is the literature-reported per-allele magnitude of association of the *i*th SNP for height and BMI, respectively. A scaling factor was calculated by regressing each GS against its respective trait among non-case carriers. The corresponding regression coefficients were *β*_0_ (intercept = 165.455) and *β*_1_ (slope = 5.217) for height and *β*_0_ (22.607) and *β*_1_ (5.523) for BMI. In the present study, BMI-GS was scaled to BMI at the date of questionnaire, rather than BMI at age 18 years, as previous GWAS were based on BMI measurements in middle-aged adults.

We subsequently modelled each scaled GS against ovarian cancer risk using weighted Cox models. Our primary outcome of interest was ovarian cancer diagnosis, with individuals censored for breast cancer diagnosis, risk-reducing bilateral salpingo-oophorectomy, death, or end of follow-up, whichever occurred first. Owing to the study design of CIMBA, weights in the model were applied for cases and non-cases based on previously observed incidence of ovarian cancer in *BRCA1/2* carriers.^26,27^ We applied a robust sandwich variance-estimation approach to the risk estimates to account for non-independence among multiple carriers per family. In addition, we performed subgroup analyses by *BRCA1/2* mutations and menopausal status. Menopausal status was defined as a time-varying covariate, coded as premenopausal from birth until age at natural menopause or bilateral salpingo-oophorectomy. For individuals with missing age at menopause, we imputed the age as 50 years. Imputing missing age at menopause as 46 years did not materially change the results. The mean and median ages at natural menopause in this population were 46 and 48 years, respectively. All analyses were adjusted for the first eight principal components (to account for ethnicity and population stratification), birth cohort, and country of enrolment. Additional analyses assessed the associations of height and BMI with ovarian cancer subgroups by histological type (serous vs. non-serous) and by tumour grade (well or moderately differentiated tumours vs. poorly or undifferentiated).

In addition, phenotype associations with each individual height and BMI variant were assessed and pooled using inverse variance-weighted meta-analysis. The individual associations were obtained by first extracting *β*_*XGi*_ for each SNP *i*, which represents the per-allele magnitude of association with height or BMI from previous GWAS. Next, we calculated *β*_*YGi*_ and $SE\left( {\beta _{YGi}} \right)$ using multivariate-adjusted weighted Cox models for each SNP using the CIMBA data, where ovarian cancer risk is predicted by genotype *G* (with *G* = 0, 1, 2 for the allele corresponding to greater height or BMI), principal components, birth cohort, *BRCA* mutation, and country of enrolment. The overall causal association (*β*_*YX*_) is calculated using inverse-variance weighted estimate of each variant’s effect: $\beta _{YX} = \frac{{\mathop {\sum }\nolimits_i \beta _{XGi}\beta _{YGi}SE(\beta _{YGi})^{ - 2}}}{{\mathop {\sum }\nolimits_i \beta _{XGi}^2SE(\beta _{YGi})^{ - 2}}}$. Standard error was estimated as $SE_{YX} = \sqrt {\frac{1}{{\mathop {\sum }\nolimits_i \beta _{XGi}^2SE(\beta _{YGi})^{ - 2}}}}$ using the Burgess’s method.^19,28^ Egger’s test was used to assess for possible pleiotropic effects of the variants (i.e. whether variants influence the outcome through other pathways), to ensure that this assumption held.^29^

Finally, in participants with available data on height and BMI, we conducted a formal IV analysis using the method of two-stage residual inclusion regression.^30^ In stage one, observed height or BMI was regressed against the corresponding GS, principal components, birth cohort, country, and mutation status. In the second stage, we used a Cox model to fit ovarian cancer risk against height or BMI, birth cohort, country, mutation status, and residuals from stage one. Variance estimates were obtained through 10,000 boot-straps (see details in Supplementary Methods). In these individuals, we also analysed the association between observed measurements of height or BMI and ovarian cancer risk using weighted Cox models, adjusted for established ovarian cancer risk factors, including birth cohort, menopausal status, age at menarche (years), and parity (continuous). The BMI values used were obtained at the date of questionnaire, usually close to the date of genetic testing and recalled for BMI at age 18 years.

In models with menopausal status as time-varying variable, the test for heterogeneity by menopausal status was essentially a test of the proportional hazards assumption. All analyses were performed using SAS 9.4 (SAS Institute, Cary, NC) and Stata 14.0 (StataCorp, College Station, TX). A two-sided *P*-value < 0.05 was considered statistically significant unless stated otherwise.

## Results

### Demographic and clinical characteristics

Characteristics for the 22,588 individuals in the CIMBA consortium, comprising 14,676 *BRCA1* and 7912 *BRCA2* mutation carriers, are shown in Table 1. We documented 2923 women with ovarian cancer (*BRCA1:* 2319; *BRCA2*: 604). Compared with non-cases, participants who developed ovarian cancer were more often parous women, were younger at first live birth, and were from earlier birth cohorts. At the date of questionnaire/interview, height measurement was available for 7657 participants and BMI measurement for 7516 participants. Most tumours for *BRCA1/2* mutation carriers were invasive, of serous, poorly, or undifferentiated grade, and stages 3 or 4 at diagnosis, characteristics which are consistent with prior reports.^31^

**Table 1:** Baseline characteristics of participants in the CIMBA consortium with genotype information

Variable	Ovarian cancer cases, *N* = 2923	Non-cases, *N* = 19,665	*P* value^b^
Mutation carrier status			<0.0001
* BRCA1*	2319 (79.3)	12,357 (62.8)	
* BRCA2*	604 (20.7)	7308 (37.2)	
Year of birth, median (IQR)	1948 (1940, 1955)	1960 (1951, 1969)	<0.0001
Age at diagnosis or censoring, years (mean ± SD)	52.5 ± 9.8	44.7 ± 12.4	<0.0001
Ethnicity, *n* (%)			0.07
Caucasian, not otherwise specified	2060 (89.7)	13,613 (88.4)	
Ashkenazi Jewish	237 (10.3)	1780 (11.6)	
Height in cm, *n*	784	6873	
Mean ± SD	163.2 ± 6.5	164.8 ± 6.9	<0.0001
Weight at baseline^a^ in kg, *n*	780	6789	
Mean ± SD	69.0 ± 14.6	68.5 ± 14.1	0.32
Body mass index at baseline^a^ in kg/m^2^, *n*	772	6744	
Mean ± SD	25.9 ± 5.3	25.2 ± 5.1	0.0002
Weight in early adulthood in kg, *n*	536	4,912	
Mean ± SD	56.5 ± 8.3	57.9 ± 9.5	0.0007
Body mass index in early adulthood in kg/m^2^, *n*	536	4881	
Mean ± SD	21.2 ± 3.0	21.3 ± 3.3	0.43
Age at menarche in years, *n*	771	6688	
Mean ± SD	13.0 ± 1.5	13.0 ± 1.5	0.90
Parous, *n* (%)			<0.0001
Yes	805 (88.3)	5790 (77.4)	
No	107 (11.7)	1692 (22.6)	
Age at first live birth in years, *n*	735	5555	
Mean ± SD	24.4 ± 4.5	25.4 ± 4.9	<0.0001
Menopausal status, *n* (%)			<0.0001
Premenopausal	112 (11.5)	3816 (51.1)	
Postmenopausal	863 (88.5)	3654 (48.9)	
Age at menopause, years (mean ± SD)	46.8 ± 5.7	44.7 ± 6.1	<0.0001
Tumour behaviour, *n* (%)			
Invasive	1228 (99.2)		
Borderline	10 (0.8)		
Tumour histotype, *n* (%)			
Serous	892 (67.9)		
Mucinous	20 (1.5)		
Endometrioid	141 (10.7)		
Clear cell	17 (1.3)		
Other	243 (18.5)		
Tumour grade, *n* (%)			
Well differentiated	43 (4.6)		
Moderately differentiated	196 (21.0)		
Poorly/undifferentiated	696 (74.4)		
Tumour stage, *n* (%)			
Borderline	2 (0.3)		
Stage 1	121 (16.4)		
Stage 2	93 (12.6)		
Stage 3	412 (55.7)		
Stage 4	112 (15.1)		

*CIMBA* Consortium of Investigators for the Modifiers of *BRCA1/2*, *IQR* interquartile range, *SD* standard deviation

^a^Reported at the date of questionnaire

^b^*P* values for comparing cases and non-cases were calculated from logistic regression models with robust sandwich variance estimator

### Observed and predicted height on risk of ovarian cancer

In the survival modelling of ovarian cancer risk, age was used as the underlying timescale and the numbers of individuals retained in the analysis were 20535, 14647, 7375, and 2832 at ages 30, 40, 50, and 60 years, respectively, suggesting that statistical power for the late age is limited. After adjustment for birth cohort, country of enrolment, mutation, menopausal status, and principal components, a nonsignificant association was found for observed height and ovarian cancer risk (hazard ratio (HR) = 1.07 per 10-cm increase, 95% confidence interval (CI): 0.94–1.23, *P* = 0.31) (Table 2). We found broadly consistent associations of height in both *BRCA1* and *BRCA2* mutation carriers by menopausal status and by tumour histological type and grade.

**Table 2:** Association of height and ovarian cancer risk using observed height among 7657 participants

	N/events	HR (95% CI)	*P* value	
Per 10 cm increase in observed height				
All participants (confounding adjustment sequentially)				
Adjusted for principal components	7657/784	1.12 (0.97–1.29)		0.12
Additionally adjusted for country	7657/784	1.15 (1.00–1.32)		0.06
Additionally adjusted for birth cohort	7657/784	1.05 (0.91–1.21)		0.53
Additionally adjusted for mutation status	7657/784	1.06 (0.92–1.22)		0.42
** Additionally adjusted for menopausal status**	**7657/784**	**1.07 (0.94**–**1.23)**		**0.31**
Additionally adjusted for parity and age at menarche	7090/724	1.09 (0.94–1.26)		0.24
By mutation status^a^				
* BRCA1* carrier	4502/552	1.07 (0.91–1.24)		0.42
* BRCA2* carrier	3155/232	1.11 (0.85–1.45)		0.44
* P*_interaction_				0.64
By menopausal status^b^				
Premenopausal	7657/105	1.02 (0.72–1.42)		0.93
Postmenopausal	4328/679	1.09 (0.94–1.26)		0.27
* P*_interaction_				0.71
By tumour subtype^c^				
Serous	7360/319	1.07 (0.87–1.31)		0.52
Non-serous^d^	7360/168	1.30 (1.01–1.68)		0.045
* P*_het_				0.24
By tumour grade^c^				
Well or moderately differentiated	7252/111	1.12 (0.83–1.52)		0.46
Poorly/undifferentiated	7252/268	1.15 (0.93–1.43)		0.19
* P*_het_				0.89

*HR* hazard ratio, *CI* confidence interval

^a^Adjusted for principal components, birth cohort, country of enrolment, and menopausal status in weighted Cox model

^b^Adjusted for principal components, mutation status, birth cohort, and country of enrolment

^c^Adjusted for principal components, birth cohort, country of enrolment, mutation status, and menopausal status

^d^Includes endometrioid, mucinous, clear cell, and other histologic types

Bolded line refers to the model corresponding to our main results

The height GS was significantly associated with height in all participants, in ovarian cancer cases, and in non-case participants (all *P* < 10^−24^) (Supplementary Table 3). Overall, approximately 13.4% of the variation in height was explained by the height GS. Besides height, we found weaker associations between the height GS and body weight and age at menarche.

In MR analysis, height GS had a nonsignificant positive association with ovarian cancer risk, HR = 1.02 per 10-cm increase in genetically predicted height, 95% CI: 0.85–1.23, *P* = 0.82 (Table 3). We found similar associations by subgroups of mutation, menopausal status, and tumour grade.

**Table 3:** Association of height and ovarian cancer risk among 22,588 participants in CIMBA per 10-cm increase in genetically predicted height

	*N*/events	HR (95% CI)	*P* value	Heterogeneity (*I*^2^)
Height GS^a^				
All participants (confounding adjustment sequentially)				
Adjusted for principal components	22,588/2923	0.99 (0.82–1.19)	0.89	
Additionally adjusted for country	22,588/2923	0.97 (0.81–1.17)	0.77	
Additionally adjusted for birth cohort	22,588/2923	0.98 (0.82–1.18)	0.83	
Additionally adjusted for mutation status	22,588/2923	1.02 (0.85–1.22)	0.13	
** Additionally adjusted for menopausal status**	22,588/2923	**1.02 (0.85–1.23)**	**0.82**	
By mutation status^b^				
* BRCA1* carrier	14,676/2319	1.02 (0.83–1.25)	0.87	
* BRCA2* carrier	7912/604	1.04 (0.68–1.57)	0.87	
* P*_interaction_			0.99	
By menopausal status^c^				
Premenopausal	22,588/967	0.96 (0.73–1.26)	0.77	
Postmenopausal	9219/1955	1.08 (0.85–1.38)	0.52	
* P*_interaction_			0.50	
By tumour subtype^d^				
Serous	20,978/892	1.36 (0.97–1.90)	0.08	
Non-serous	20,978/421	0.95 (0.58–1.56)	0.84	
* P*_het_			0.25	
By tumour grade^d^				
Well or moderately differentiated	20,600/239	1.63 (0.86–3.09)	0.14	
Poorly/undifferentiated	20,600/696	1.20 (0.82–1.74)	0.35	
* P*_het_			0.42	
Meta-analysis method^e^				
All participants	22,588/2923	1.02 (0.83–1.26)	0.83	0.0%
* BRCA1* carrier	14,676/2319	1.02 (0.81–1.28)	0.89	0.0%
* BRCA2* carrier	7912/604	1.05 (0.67–1.66)	0.82	7.0%
* P*_interaction_			0.89	
Two-stage residual inclusion method^f^				
All participants	7657/784	1.20 (0.86–1.69)	0.29	
* BRCA1* carrier	4502/552	1.40 (0.94–2.10)	0.10	
* BRCA2* carrier	3155/232	0.93 (0.49–1.74)	0.81	

*HR* hazard ratio, *CI* confidence interval, *CIMBA* Consortium of Investigators for the Modifiers of *BRCA1/2*, *GS* genetic score

^a^Height genetic score combining 586 height-associated single-nucleotide polymorphisms (SNPs)

^b^Adjusted for principal components, birth cohort, country of enrolment, and menopausal status in weighted Cox model

^c^Adjusted for principal components, mutation status, birth cohort, and country of enrolment

^d^Adjusted for principal components, mutation status, menopausal status, birth cohort, and country of enrolment

^e^HRs were calculated using inverse-variance meta-analysis and re-scaled to the corresponding units by calculating the height measurements per *z*-score among controls. Effect estimates for ovarian cancer for each SNP were calculated from weighted Cox model adjusting for principal components, birth cohort, country of enrolment, menopausal status, and mutation status

^f^Analysis was performed among 7657 participants with measured height

Bolded line refers to the model corresponding to our main results

Combining the effects of all 586 height-associated variants using inverse-variance weighted meta-analysis, we obtained similar findings (HR = 1.02, 95% CI: 0.83–1.26, *P* = 0.83) (*λ*). Among the SNPs that were combined, there was a low degree of heterogeneity (*I*^2^ = 0%). Examining small-study effects using Egger’s test did not suggest likely pleiotropic effects. In the two-stage residual inclusion analysis, the estimated relative risk was larger though with wide CIs, which overlapped with those derived using other methods (HR = 1.20, 95% CI: 0.86–1.69, *P* = 0.29).

### Observed and predicted BMI on risk of ovarian cancer

After multivariable adjustment, we found a nonsignificant positive association between BMI at the date of questionnaire completion and ovarian cancer risk, HR = 1.04 per 5-kg/m^2^ increase in BMI, 95% CI: 0.95–1.14, *P* = 0.42 (Table 4). In a pre-specified analysis, the association between BMI and ovarian cancer risk was stronger in premenopausal women (HR = 1.25, 95% CI: 1.06–1.48; *P* = 0.009), whereas no association was found in postmenopausal women (HR = 0.98, 95% CI: 0.88–1.10), with significant interaction (*P* = 0.02). We found that BMI was a significant predictor of non-serous ovarian cancer risk (HR = 1.25, 95% CI: 1.06–1.49) but not for serous ovarian cancer (HR = 0.98, 95% CI: 0.84–1.15).

**Table 4:** Association of body mass index (BMI) and ovarian cancer risk using observed BMI

	*N*/events	HR (95% CI)	*P* value
Per 5 kg/m^2^ increase in BMI at date of questionnaire			
All participants (confounding adjustment sequentially)			
Adjusted for principal components	7516/772	1.00 (0.90–1.10)	0.96
Additionally adjusted for country	7516/772	0.99 (0.90–1.09)	0.84
Additionally adjusted for birth cohort	7516/772	1.02 (0.93–1.12)	0.72
Additionally adjusted for mutation status	7516/772	1.06 (0.96–1.16)	0.26
** Additionally adjusted for menopausal status**	**7516/772**	**1.04 (0.95–1.14)**	**0.42**
Additionally adjusted for parity and age at menarche	6964/715	1.04 (0.94–1.14)	0.48
By mutation status^a^			
* BRCA1* carrier	4401/543	1.06 (0.95–1.17)	0.31
* BRCA2* carrier	3115/229	0.96 (0.81–1.15)	0.67
* P*_interaction_			0.35
By menopausal status^b^			
** Premenopausal**	**7516/102**	**1.25 (1.06**–**1.48)**	**0.009**
Postmenopausal	4257/670	0.98 (0.88–1.10)	0.78
*** P***_**interaction**_			**0.02**
By tumour subtype^c^			
Serous	7223/312	0.98 (0.84–1.15)	0.83
** Non-serous**^**d**^	**7223/167**	**1.25 (1.06**–**1.49)**	**0.01**
* P*_*het*_			**0.04**
By tumour grade^c^			
Well or moderately differentiated	7252/109	1.05 (0.84–1.32)	0.65
Poorly/undifferentiated	7252/268	0.95 (0.82–1.11)	0.54
* P*_het_			0.47
Per 5 kg/m^2^ increase in BMI in young adulthood			
All participants (confounding adjustment sequentially)			
Unadjusted	5417/536	0.86 (0.69–1.07)	0.17
Adjusted for country	5417/536	0.86 (0.69–1.08)	0.19
Additionally adjusted for birth cohort	5417/536	0.87 (0.70–1.08)	0.21
Additionally adjusted for mutation status	5417/536	0.91 (0.73–1.13)	0.39
Additionally adjusted for menopausal status	5417/536	0.93 (0.76–1.16)	0.53
Additionally adjusted for parity and age at menarche	5210/516	0.92 (0.74–1.14)	0.42
By mutation status^a^			
* BRCA1* carrier	3134/380	0.92 (0.71–1.18)	0.50
* BRCA2* carrier	2283/156	1.00 (0.74–1.36)	0.99
* P*_interaction_			0.73
By menopausal status^b^			
** Premenopausal**	**5417/67**	**1.34 (0.97**–**1.84)**	**0.07**
Postmenopausal	3094/469	0.82 (0.65–1.04)	0.11
*** P***_**interaction**_			**0.01**

*HR* hazard ratio, *CI* confidence interval

^a^Adjusted for principal components, birth cohort, country of enrolment, and menopausal status in weighted Cox model

^b^Adjusted for principal components, mutation status, birth cohort, and country of enrolment

^c^Adjusted for principal components, birth cohort, country of enrolment, mutation status, and menopausal status

^d^Includes endometrioid, mucinous, clear cell, and other histological types

Bolded lines refer to the model corresponding to our main results

Similar to BMI at the date of questionnaire completion, we detected a significant interaction of BMI in young adulthood and menopausal status (*P* = 0.01), with a stronger association for premenopausal women (HR = 1.34, 95% CI: 0.97–1.84) compared with postmenopausal women (HR = 0.82, 95% CI: 0.65–1.04).

BMI-GS was strongly associated with BMI at both the date of questionnaire completion and young adulthood (Supplementary Table 4). Overall, the BMI-GS explained 2.6% of the variation in BMI at the date of questionnaire completion and 1.7% of the variation in young adulthood BMI. We found associations between the BMI-GS and height and age at menarche, though the strength of the association was weaker than the association with BMI.

In the entire consortium, the BMI-GS had a nonsignificant positive association with ovarian cancer risk with a HR = 1.10 per 5-kg/m^2^ of genetically predicted BMI, 95% CI: 0.86–1.42, *P* = 0.44 (Table 5). We found heterogeneity by menopausal status (*P* = 0.006). BMI-GS was positively associated with ovarian cancer risk in premenopausal women (HR = 1.59, 95% CI: 1.08–2.33) but not in postmenopausal women (HR = 0.80, 95% CI: 0.58–1.11). BMI-GS also tended to be more associated with non-serous (HR = 1.60, 95% CI: 0.83–3.08) than serous tumours (HR = 0.92, 95% CI: 0.59–1.43).

**Table 5:** Association of body mass index genetic score (BMI-GS) and ovarian cancer risk among 22,588 participants in CIMBA, per 5 kg/m^2^ increase in genetically predicted BMI

Breast cancer group	*N*/events	HR (95% CI)	*P* value	Heterogeneity (*I*^2^)
BMI-GS^a^				
All participants (confounding adjustment sequentially)				
Adjusted for principal components	22,588/2923	1.12 (0.87–1.45)	0.37	
Additionally adjusted for country	22,588/2923	1.11 (0.86–1.44)	0.41	
Additionally adjusted for birth cohort	22,588/2923	1.12 (0.87–1.45)	0.36	
Additionally adjusted for mutation status	22,588/2923	1.11 (0.86–1.42)	0.43	
** Additionally adjusted for menopausal status**	**22,588/2923**	**1.10 (0.86–1.42)**	**0.44**	
By mutation status^b^				
* BRCA1* carrier	14,676/2319	1.16 (0.88–1.53)	0.31	
* BRCA2* carrier	7912/604	0.81 (0.46–1.43)	0.46	
* P*_interaction_			0.27	
By menopausal status^c^				
** Premenopausal**	**22,588/967**	**1.59 (1.08–2.33)**	**0.02**	
Postmenopausal	9219/1955	0.80 (0.58–1.11)	0.18	
*** P***_**interaction**_			**0.006**	
By tumour subtype^d^				
Serous	20,978/892	0.92 (0.59–1.43)	0.71	
** Non-serous**	**20,978/421**	**1.60 (0.83–3.08)**	**0.16**	
*** P***_**het**_			**0.17**	
By tumour grade^d^				
Well or moderately differentiated	20,600/239	1.20 (0.52–2.75)	0.67	
Poorly/undifferentiated	20,600/696	0.74 (0.45–1.21)	0.23	
* P*_het_			0.33	
Meta-analysis method^e^				
All participants	22,588/2923	1.12 (0.86–1.46)	0.39	15.9%
* BRCA1* carrier	14,676/2319	1.18 (0.88–1.57)	0.26	17.2%
* BRCA2* carrier	7912/604	0.80 (0.45–1.43)	0.45	0.0%
* P*_interaction_			0.24	
Two-stage residual inclusion method^f^				
All participants	7516/772	1.37 (0.84–2.24)	0.21	
* BRCA1* carrier	4401/543	1.24 (0.67–2.27)	0.49	
* BRCA2* carrier	3115/229	1.57 (0.67–3.66)	0.30	

*HR* hazard ratio, *CI* confidence interval, *CIMBA* Consortium of Investigators for the Modifiers of *BRCA1/2*

^a^BMI-GS was constructed by combining 93 BMI-associated single-nucleotide polymorphisms (SNPs)

^b^Adjusted for principal components, birth cohort, country of enrolment, and menopausal status in weighted Cox model

^c^Adjusted for principal components, mutation status, birth cohort, and country of enrolment

^d^Adjusted for principal components, mutation status, menopausal status, birth cohort, and country of enrolment

^e^Hazard ratios were calculated using inverse-variance meta-analysis and re-scaled to the corresponding units by calculating the height measurements per *z*-score among controls. Effect estimates for ovarian cancer for each SNP were calculated from weighted Cox model adjusting for principal components, birth cohort, country of enrolment, menopausal status, and mutation status

^f^Analysis was performed among 7516 participants with measured BMI

Bolded lines refer to the model corresponding to our main results

We found similar results when we statistically combined the associations of the 93 BMI-associated variants, with an overall HR = 1.12, 95% CI: 0.86–1.46. Heterogeneity was low (*I*^2^ = 15.9%), indicating a low likelihood of pleiotropic associations. Using the two-stage residual inclusion approach, we found a generally similar association (HR = 1.37, 95% CI: 0.84–2.24, *P* = 0.21).

### Individual SNPs and ovarian cancer risk

We found 22 height-associated and 4 BMI-associated SNPs that were nominally associated with ovarian cancer risk (*P* < 0.05; Table 6). None of these SNPs were significantly associated with ovarian cancer risk after correcting for multiple testing. We cross-checked these identified SNPs with the most up-to-date list of ovarian cancer susceptibility SNPs and did not find any overlaps.^32^

**Table 6:** Height or body mass index (BMI) single-nucleotide polymorphisms (SNPs) statistically significantly associated (*P* < 0.05) with ovarian cancer risk in CIMBA

rs ID	Chromosome	Position	Nearest gene	Reference allele in CIMBA	Effect allele in CIMBA	Effect allele frequency in CIMBA	Imputation quality^a^	Association with ovarian cancer in CIMBA		
								Log hazard ratio^b^	Standard error	*P* value
Height										
rs11049611	12	28600244	*CCDC91*	C	T	0.28	1	0.127	0.036	0.0004
rs6902771	6	152157881	*ESR1*	C	T	0.46	0.98	0.091	0.032	0.005
rs584828	17	38599230	*IGFBP4*	C	T	0.39	0.68	0.109	0.040	0.006
rs3817428	15	89415247	*ACAN*	C	G	0.22	0.51	0.144	0.053	0.006
rs7517682	1	103519589	*COL11A1*	G	A	0.56	0.98	0.085	0.033	0.009
rs12470505	2	219908369	*CCDC108*	T	G	0.10	0.97	−0.143	0.055	0.009
rs26024	5	127696022	*FBN2*	A	C	0.34	1	−0.087	0.034	0.011
rs13113518	4	56399648	*CLOCK*	T	C	0.37	0.99	0.081	0.033	0.014
rs7319045	13	92024574	*GPC5*	A	G	0.61	0.92	0.084	0.035	0.017
rs2044124	17	61845425	*CCDC47*	T	C	0.95	0.91	0.187	0.079	0.018
rs9309101	2	43629612	*THADA*	A	G	0.35	1	0.076	0.033	0.021
rs11867943	17	54229842	*ANKFN1*	A	T	0.11	0.96	0.118	0.051	0.022
rs12779328	10	12943973	*CCDC3*	C	T	0.30	0.94	−0.080	0.036	0.026
rs8073371	17	46096276	*COPZ2*	C	T	0.20	1.00	−0.095	0.043	0.029
rs2013265	8	24092500	*ADAM28*	C	T	0.22	0.62	0.104	0.047	0.029
rs11687941	2	242191410	*HDLBP*	C	G	0.26	0.96	−0.079	0.037	0.031
rs6838153	4	122720999	*EXOSC9*	A	G	0.33	0.99	−0.072	0.034	0.033
rs7112925	11	66826160	*RHOD*	C	T	0.36	0.95	−0.071	0.034	0.037
rs16942341	15	89388905	*ACAN*	C	T	0.03	0.60	0.255	0.123	0.039
rs6080830	20	17771113	*BANF2*	A	G	0.43	0.68	−0.080	0.039	0.041
rs867245	4	2218888	*POLN*	C	G	0.07	1.00	0.122	0.060	0.043
rs1155939	6	126866133	*C6orf173*	C	A	0.51	0.99	0.064	0.033	0.049
BMI										
rs16851483	3	141275436	*RASA2*	G	T	0.07	1	−0.203	0.068	0.003
rs2207139	6	50845490	*TFAP2B*	A	G	0.16	0.99	0.120	0.043	0.005
rs2033732	8	85079709	*RALYL*	T	C	0.75	0.72	−0.088	0.042	0.037
rs6804842	3	25106437	*RARB*	A	G	0.58	0.58	0.087	0.044	0.046

*CIMBA* Consortium of Investigators for the Modifiers of *BRCA1/2*

^a^Imputation quality of 1 indicates genotyped SNPs

^b^Per-allele association with ovarian cancer was adjusted for principal components, birth cohort, menopausal status, age at menopause, country of enrolment, and mutation status in weighted Cox models

## Discussion

Using data from a large international consortium of *BRCA1/2* mutation carriers, we found no statistically significant association between height and ovarian cancer risk. Interestingly, we observed interactions between BMI (both observed and genetically predicted) and menopausal status on ovarian cancer risk, with increasing BMI associated with increased risk in premenopausal but not in postmenopausal women.

Our finding of a positive association between BMI and overall ovarian cancer risk in *BRCA1 and BRCA2* mutation carriers is corroborated by several prior studies in the general population.^12,14,15,33^ One MR analysis using 77 BMI-associated SNPs, conducted in the general population, found that each 1-standard deviation (SD) increment in genetically-predicted adult BMI corresponded to an odds ratio (OR) of 1.35 (95% CI: 1.05–1.72).^34^ We found that 5-kg/m^2^ (about 1 SD) increment in genetically predicted BMI was associated with an HR = 1.10 (95% CI: 0.86–1.42) in mutation carriers. However, the association of BMI with ovarian cancer risk is likely to vary by menopausal status. In the general population, significant differential association of BMI with ovarian cancer risk by menopausal status has been found in some studies^15,16,35,36^ but not in others.^12,37^ A pooled analysis of 47 epidemiologic studies with 25,157 ovarian cancer cases showed that the relative risk per 5-kg/m^2^ increase in BMI was 1.12 (95% CI: 1.07–1.17) in premenopausal women and 1.08 (95% CI: 1.04–1.12) in postmenopausal women.^12^ The largest single cohort study, with 3686 ovarian cancer cases, found that the HR per 5-kg/m^2^ increase in BMI was 1.21 (99% CI: 1.09–1.33) in premenopausal and 1.07 (99% CI: 1.02–1.12) in postmenopausal women.^15^ An MR analysis conducted in the general population also observed stronger associations for non-high-grade serous carcinomas in premenopausal women (OR = 1.62, 95% CI: 0.88–3.01) compared with postmenopausal hormone replacement therapy (HRT) users (OR = 1.26, 95% CI: 0.57–2.82) and postmenopausal HRT non-users (OR = 1.17, 95% CI: 0.61–2.24), though no formal statistical tests examining heterogeneity were performed.^14^ Similarly, we found in *BRCA1/2* mutation carriers that 5-kg/m^2^ increment in genetically predicted BMI was associated with an HR = 1.59 (95% CI: 1.08–2.33) for premenopausal ovarian cancer and an HR = 0.80 (95% CI: 0.58-1.11) for postmenopausal ovarian cancer. Studies that have not demonstrated significant variation by menopausal status tended to show that the positive association between BMI and ovarian cancer risk was primarily among those who had never used HRT.^12^ Taken together, our results and previous literature are suggestive that higher BMI may increase ovarian cancer risk in premenopausal women but not in postmenopausal women.

In addition, several studies that had sufficient numbers of cases to evaluate the relationship between BMI and ovarian cancer risk by histologic subtype have shown significant heterogeneity. Observational studies in the Ovarian Cancer Cohort Consortium found stronger associations between BMI and endometrioid (OR = 1.17 per 5-kg/m^2^, 95% CI: 1.11–1.23) or mucinous ovarian cancer (OR = 1.19, 95% CI: 1.06–1.32) but no association with serous ovarian cancer (OR = 0.98, 95% CI: 0.94–1.02).^16^ A more recent MR analysis in the same consortium using a genetic score comprised of 87 SNPs showed that a genetically predicted BMI had a stronger association with endometrioid (OR = 1.17, 95% CI: 0.87–1.59) or mucinous ovarian cancer (OR = 1.18, 95% CI: 0.84–1.67) than high-grade serous cancer (OR = 1.06, 95% CI: 0.89–1.27), though the 95% CIs for these estimates were largely overlapping.^14^ Consistent with findings in the general population, our study in *BRCA1/2* mutation carriers showed that BMI was positively associated with non-serous ovarian cancer (HR = 1.25 per 5-kg/m^2^, 95% CI: 1.06–1.49 in observed BMI and HR = 1.60, 95% CI: 0.83–3.08, per 5-kg/m^2^ in genetically predicted BMI), of which endometrioid is a major subtype. Of note, obesity is an established risk factor for endometrial cancer.^38^ However, subsequent studies with greater number of cases of different ovarian cancer subtypes are needed to assess whether the effect of obesity truly differs by tumour subtype.

Our finding of a nonsignificant positive association between height and ovarian cancer risk is also consistent with prior epidemiological studies in the general population.^12,37,39^ In the general population, 5-cm increment in height was associated with a 7% increase (95% CI: 5–9%) in ovarian cancer risk,^12^ and 5-cm increment in genetically predicted height was associated with a 6% (95% CI: 1–11%) increase in ovarian cancer risk.^39^ The associations for observed height did not differ significantly between ovarian histological types,^2,12^ while genetically predicted height had a stronger association with clear cell (OR = 1.20, 95% CI: 1.04–1.38) or low-grade/borderline serous ovarian cancers (OR = 1.15, 95% CI: 1.01–1.30) compared to high-grade serous (OR = 1.05, 95% CI: 0.99–1.11).^39^ We did not find statistically significant heterogeneity by histology in our study of mutation carriers, though point estimates varied across histology.

Several biological mechanisms potentially explain the associations observed in our study. Overweight/obese women are more likely to have anovulatory cycles and fertility issues, particularly when caused by polycystic ovarian syndrome (PCOS), and thus have an increased risk of ovarian cancer.^40,41^ The association of PCOS with ovarian cancer risk was mainly confined to premenopausal women.^42^ Some studies have suggested that *BRCA1/2* mutation carriers may have subclinical ovarian insufficiency, which could mediate the relationship between obesity-related infertility and increased ovarian cancer risk.^43^ Obesity itself also creates a proinflammatory state and adipocyte-secreted inflammatory markers have been implicated in ovarian cancer development.^44^ Circulating levels of oestradiol, androgen, and progesterone have also been implicated in the risk of ovarian cancer.^45,46^ One study in *BRCA1/2* mutation carriers showed higher oestradiol levels during each menstrual cycle compared with non-carriers, supporting the potential role of sex hormones in ovarian tumorigenesis in this population.^47^ Obese premenopausal women tend to have lower circulating levels of progesterone compared with normal weight women.^48^ Higher progesterone levels may reduce ovarian cancer risk, through upregulation of p53, leading to tumour cell apoptosis.^46,49–51^ Taken together, these pathways may explain the association of higher BMI with premenopausal ovarian cancer risk. In addition, height has been associated with higher levels of circulating insulin-like growth factor-1 (IGF-1),^52,53^ a pathway that has been implicated in tumour transformation and may exert antiapoptotic and mitogenic effects.^54,55^ Moreover, *BRCA1* may directly interact with the IGF-1 pathway to mediate cancer risk.^56^

Our study has several strengths, including large sample size, genetic scores utilising most identified height and BMI variants, several MR methods, and consistent findings between observed and genetically predicted phenotypes. Several limitations of our study should be considered. First, even with a large sample size, the CIs for most risk estimates were wide, which limits inferences about causation. While both the height- and BMI-GS were clearly associated with their respective traits, they were only able to explain 13.4% and 2.6% of the variation, respectively. This reduced the statistical precision of our risk estimates. During the preparation of our manuscript, a new genome-wide meta-analysis^57^ found a substantial number of new genetic loci related to height and BMI, increasing the amount of variation that could be explained for these two traits to 24.6% and 6.0%, respectively, although the variation that could be explained when examining these SNPs in a validation cohort was 14.0% and 2.3%. This is comparable to the amount of variation that could be explained using the set of genetic variants in our study. Including these additional SNPs may be able to improve the precision of our estimates for both height and BMI. Moreover, the inclusion of rare variants to strengthen the height and BMI genetic instruments should also be considered in future studies.^58^ Our study did not explicitly examine whether adding height or BMI (either observed or genetically predicted) to existing polygenic risk scores for ovarian cancer could further refine risk prediction. Histology was only available in a subset of ovarian cancer patients, which limits our capacity to understand subtype-specific effects of BMI and height. Our study only included women of European ancestry, which may preclude generalisation to women of other racial/ethnic groups.

In summary, our study suggests that higher BMI may be causally associated with ovarian cancer risk in *BRCA1/2* carriers, possibly more so for premenopausal women. BMI could be used to identify premenopausal women at elevated risk of ovarian cancer. Our finding of a stronger association between BMI and non-serous ovarian cancer warrants confirmation in future studies.

## Supplementary information

Supplementary Materials

## Data Availability

Owing to the sensitive nature of the data used in this study, data requests by researchers trained in maintaining human subject confidentiality may be directed to the corresponding author of this study.
