# Supplementary material for: Mendelian randomisation study of height and body mass index as modifiers of ovarian cancer risk in 22,588 BRCA1 and BRCA2 mutation carriers
Source: Br J Cancer. 2019 Jun 19;121(2):180–92. doi: 10.1038/s41416-019-0492-8 (PMC6738050; doi:10.1038/s41416-019-0492-8)
Supplement: Supplementary file 1 — Supplementary Materials [file 41416_2019_492_MOESM1_ESM.docx]

**SUPPLEMENTARY MATERIALS**

**Supplementary Methods**

*Study Population: CIMBA Consortium*

The Consortium of Investigators of Modifiers of BRCA1/2 (CIMBA) is an international collaboration developed to assess clinical and genetic risk factors in BRCA1/2 carriers and their respective associations with the development of breast and ovarian cancer [[1](#_ENREF_1), [2](#_ENREF_2)]. Investigators from 33 countries including 55 centers and multicenter consortia submitted de-identified data to a central database. The selection criteria for participants are as follows: 1) female carriers of verified disease-causing mutation in either *BRCA1* or *BRCA2* (participants were excluded if they carried mutations in both genes, N = 84) and 2), who were 18 years of age or older, with sufficient clinical data to assess risk which could include demographic/clinical characteristics, specific cancer diagnosis, ascertainment and follow-up dates, and history of prophylactic surgeries. At each center, carriers underwent interview and clinical testing or participated in a research study, after providing written informed consent in accordance with the local institutional review board. A family identifier was applied to indicate related individuals. Race/ethnicity were obtained via questionnaires in pre-defined categories or open-ended questions. Participants with self-reported non-European ancestry were excluded from the present analysis.

*Genotyping and Imputation*

The genotyping for the current analysis was conducted using the Collaborative Oncological Gene-Environment Study (iCOGS) custom array [[3-5](#_ENREF_3)]. This array was part of a multi-consortia collaboration, including the Breast Cancer Association Consortium (BCAC), Ovarian Cancer Association Consortium (OCAC), and the Prostate Cancer Association Group to Investigate Cancer Associated Alterations in the Genome (PRACTICAL), to identify and replicate common and rare genetic variants associated with the development of breast, ovarian, and prostate cancers. Development of the array was divided among the consortium in the following distribution: 25% was allotted to each of BCAC, OCAC, and PRACTICAL, 17.5% to CIMBA, and the remaining 7.5% were designed to be from common pathways that have been previously researched. Ultimately, the iCOGs array comprised of 220,123 SNPs, of which 211,155 were successfully manufactured for genotyping.

Genotyping for *BRCA1* carriers was conducted at the Mayo Clinic (USA) and for *BRCA2* carriers at McGill University and Génome Québec Innovation Centre (both in Canada). Genotypes identified through the iCOGs array were called using the Illumina GenCall algorithm [[3](#_ENREF_3), [4](#_ENREF_4)]. A total of 270 samples from Hapmap2 were used to generate a cluster file for the initial file. For the final call, 3,018 individuals were selected, which included samples from each of the study centers, consortia, and ethnicities from the iCOGs collaboration. Plates at this stage that have a consistently high call rate were used. A different cluster file was generated using 380 genotyped samples from the 1000 Genomes project and Hapmap including European, African, and Asian ethnicity along with 160 positive control samples for rare variants for the iCOGs array. This file was used to call the remaining genotype samples. Subsequently, additional exclusion criteria for quality assurance were applied: 1) single nucleotide polymorphisms (SNPs) located on the Y chromosome, 2) monomorphic SNPs, 3) SNPs that deviated significantly from Hardy-Weinberg equilibrium (*P* < 10^-7^), 4) SNPs with call rates < 95%, or 5) SNPs demonstrating extreme heterozygosity [[3](#_ENREF_3), [5](#_ENREF_5)]. After applying these quality control measures to the original 211,155 SNPs from the iCOGs array, we included data on 200,720 SNPs in *BRCA1* mutation carriers and 200,908 SNPs in *BRCA2* mutation carriers. For SNPs not genotyped on the iCOGS custom array, imputation was performed using the IMPUTE2 software based on the January 2012 version of the 1,000 Genomes haplotype [[6](#_ENREF_6)]. SNPs were excluded from the analysis if they had imputation quality *r^2^* < 0.30. After applying these quality control and exclusion criteria, the current analysis included genotype data from 14,676 *BRCA1* carriers (7,360 of them developed breast cancer) and 7,912 *BRCA2* carriers (4,091 of them developed breast cancer).

Principal components analysis was conducted using 33,661 autosomal SNPs (MAF > 0.05) that were weakly correlated with one another (pairwise *r^2^* < 0.1) with 267 HapMap samples (ancestries represented included CHB, JPT, YRI, and CEU) [[3](#_ENREF_3)]. The first 15 principal components were available for analysis in the present study.

*Two-stage Residual Inclusion Regression*

In the main instrumental variable (IV) analysis of the data, we have constructed the literature-reported per-allele associations between BMI and SNPs to build a genetic score (GS) for BMI using the formula: $BMI-GS=\sum_{i=1}^{93} \beta_{XGi}{SNP}_{i}$. Then we used $BMI-GS$ in the Cox regression of ovarian cancer risk. This method and the inverse variance-weighted meta-analysis method can both be considered as two-stage predictor substitution (2SPS) method, while the predictor is from the external genome-wide association of BMI in the literature. For non-linear models, the 2SPS methods may give biased estimates in theory [[7](#_ENREF_7)]. An alternative method of the two-stage instrumental variable analysis is the two-stage residual inclusion (2SRI) method, which can give consistent estimates [[7](#_ENREF_7), [8](#_ENREF_8)]. For the present study, we described the 2SRI regression using BMI as an example. In the first stage, we fit a linear model of observed BMI as below:

$$BMI=\alpha_{0}+\beta_{xg}GS+\boldsymbol{\alpha}_{\boldsymbol{2}}\boldsymbol{birthcohort}+\boldsymbol{\alpha}_{\boldsymbol{3}}\boldsymbol{country}_{\boldsymbol{j}}\boldsymbol{+}\boldsymbol{\alpha}_{\boldsymbol{4}}mutation+\boldsymbol{\alpha}_{\boldsymbol{4}}\boldsymbol{PC}+e$$

where $\beta_{xg}$ is the estimated effect of genetic score in the current BRCA1/2 study sample, ***birthcohort***, ***country, PC*** *(principal components)* are vectors of the dummy variables the three factors, and $e$ is the residual of the regression. Under the assumptions of instrumental variable analysis, $e$ can represent the unobserved confounders. so if it is adjusted for, then the confounding effect for BMI would cease to exist.

In the second stage, we fit a stratified Cox model of ovarian cancer including both observed BMI and the residual *e* as below:

$$\log\left( \frac{h_{j}\left( t \right)}{h_{j}\left( 0 \right)} \right)=\beta_{yx}BMI+\beta_{1}e+\boldsymbol{\beta}_{\boldsymbol{2}}\boldsymbol{birthcohort}+\beta_{3}mutation+\boldsymbol{\beta}_{\boldsymbol{4}}\boldsymbol{PC}$$

where $\beta_{yx}$ is the IV estimate of interest, and *j* indicates category of study country for stratified Cox model. In both stage 1 and 2 of the model, we used the robust sandwich estimator of variance to account for non-independence among multiple carriers per family, but there is no easy close-form formula to calculate variance for the simultaneous two equation model. Therefore, we applied the bootstrapping method to estimate variance. In brief, we generated 10,000 randomly selected datasets with replacement, run the 2SRI 10,000 times, and the variation in $\beta_{yx}$ estimates was used to calculate its 95% confidence interval.

Although the 2SRI method is preferred for non-linear IV regression in theory, its estimator $\beta_{yx}$ is less stable than the 2SPS estimator $\beta_{YX}$ because the 2SPS method utilized $\beta_{XGi}$ directly and data from all study participants even if BMI was missing. Therefore, we considered the 2SRI method as a sensitivity analysis.

**References**

1. Rebbeck TR, Mitra N, Wan F*, et al.* Association of type and location of BRCA1 and BRCA2 mutations with risk of breast and ovarian cancer. JAMA 2015;313(13):1347-61.

2. Chenevix-Trench G, Milne RL, Antoniou AC*, et al.* An international initiative to identify genetic modifiers of cancer risk in BRCA1 and BRCA2 mutation carriers: the Consortium of Investigators of Modifiers of BRCA1 and BRCA2 (CIMBA). Breast cancer research : BCR 2007;9(2):104.

3. Couch FJ, Wang X, McGuffog L*, et al.* Genome-wide association study in BRCA1 mutation carriers identifies novel loci associated with breast and ovarian cancer risk. PLoS Genet 2013;9(3):e1003212.

4. Gaudet MM, Kuchenbaecker KB, Vijai J*, et al.* Identification of a BRCA2-specific modifier locus at 6p24 related to breast cancer risk. PLoS Genet 2013;9(3):e1003173.

5. Kuchenbaecker KB, Neuhausen SL, Robson M*, et al.* Associations of common breast cancer susceptibility alleles with risk of breast cancer subtypes in BRCA1 and BRCA2 mutation carriers. Breast Cancer Res 2014;16(6):3416.

6. Howie B, Marchini J, Stephens M. Genotype imputation with thousands of genomes. G3 (Bethesda) 2011;1(6):457-70.

7. Terza JV, Basu A, Rathouz PJ. Two-stage residual inclusion estimation: addressing endogeneity in health econometric modeling. J Health Econ 2008;27(3):531-43.

8. Wooldridge JM. *Econometric Analysis of Cross Section and Panel Data*. Cambridge, MA: MIT Press; 2002.

**The institutional committees that approved individual studies**

The University of Melbourne Health Sciences Human Ethics Sub-Committee

Northern California Cancer Center Institutional Review Board

Columbia University Medical Center Institutional Review Board

Mount Sinai Hospital Research Ethics Board

Institutional Review Board Fox Chase Cancer Center

Institutional Review Board University of Utah

Centrālā medicīnas ētikas Komiteja; Lietuvos Bioetikos Komitetas

University of Pretoria and Pretoria Academic Hospitals Ethics Committee

City of Hope Medical Center Institutional Review Board

De Videnskabsetiske Komiteer I Region Hovedsladen

Instituto de Salud Carlos III Comité de Bioética y Bienestar Animal

City of Hope Institutional Review Board

Comitato Etico Indipendente della Fondazione IRCCS "Istituto Nazionale dei Tumori"

Bioethics committee of NCSR ‘‘Demokritos’’, 240/EHΔ/11.3

Papageorgiou Hospital Ethics Committee

Dana Farber Cancer Institute Institutional Review Board

Ethik-Kommission des Klinikums der Universität

Hospital Universitario de San Ignacio Comité de Investigaciones y Etica

Shaukat Khanum Memorial Cancer Hospital and Research Centre Institutional Review Board

Anglia & Oxford MREC

Universitair Ziekenhuis Gent - Ethics Committee

Ethik-Kommission der Medizinischen Fakultät der Universät zu Köln

Comité consultatif sur le traitement de I'information en matière de recherche dans le domaine de la santé

MedStar Research Institute - Georgetown University Oncology Institutional Review Board

Cancer Prevention and Control Protocol Review Committee

Comité Ético de Investigación Clínia Hospital Clínico San Carlos

Helsingin ja uudenmaan sairaanhoitopiiri (Helsinki University Central Hospital ethics committee)

Protocol Toetsingscommissie van het Nederlands Kanker Instituut/Antoni van Leeuwenhoek Ziekenhuis

Institutional Review Board of the Hungarian National Institute of Oncology

The Hospital Universitario Vall d'Hebron Clinical Research Ethics Committee

Catalan Institute of Oncology Institutional Review Board

Komisji Bioetycznej Pomorskiej Akademii Medycznej (Pomeranian Medical University Bioethics Committee)

Vísindasiđanefnd National Boethics Committee

Comité d'éthique de la recherche du Centre Hospitalier Universitaire de Québec

Centro Oncologico Regionale Azienda Ospedale Di Padova Comitato Etico

Peter MacCallum Cancer Centre Ethics Committee

Queensland Institute of Medical Research - Human Research Ethics Committee

Institutional Review Board of NorthShore University HealthSystem

Mayo Clinic Institutional Review Boards

McGill Faculty of Medicine Institutional Review Board

Memorial Sloan-Kettering Cancer Center Institutional Review Board

Human Biospecimen Utilization Committee

Ethikkommission der Medizinischen Universität Wien

NIH Ethics Office

Carmel Medical Center Institutional Review Board (Helsinki Committee)

N.N. Petrov Institional Ethical Committee

University Health Network Research Ethics Board

The Ohio State University Cancer Institutional Review Board

Den Videnskabsetiske Komité for Region Syddanmark

Azienda Ospedaliera Pisana Comitato Etico per lo studio del farmaco sull'uomo

Regionala Etikprövningsnämnden Stockholm

The University of Chicago Biological Sciences Division Institutional Review Board

UCLA Institutional Review Board (UCLA IRB)

Human Research Protection Program Institutional Review Board

Cambridge Local Research Ethics Committee

Roswell Park Cancer Institute Institutional Review Board

University of Pennsylvania Institutional Review Board

University of Texas MD Anderson Cancer Center Office of Protocol Research Institutional Review Board

Cedars-Sinai Medical Center (CSMC) Institutional Review Board

University of Toronto Health Sciences Review Ethics Board

Chaim Sheba Medical Center Institutional Review Board

Comissão de Ética para a Saúde do Instituto Português de Oncologia do Porto

| **Supplementary Table 1.** List of 586 height-associated genetic variants and their associations with height in prior published GWAS and in CIMBA, along with effect on ovarian cancer risk in CIMBA | | | | | | | | | | | | | | | | | | | | | | | | |
| --- | --- | --- | --- | --- | --- | --- | --- | --- | --- | --- | --- | --- | --- | --- | --- | --- | --- | --- | --- | --- | --- | --- | --- | --- |
| Rsid # | Chromosome | Position | Nearest gene | Published reference allele | Published effect allele | Effect allele frequency in published GWAS | Effect allele frequency in CIMBA | Imputation quality^1^ | Published association with height | | | | Association with height in CIMBA | | | Association with ovarian cancer in CIMBA | | | Association with ovarian cancer in *BRCA1*carriers | | | Association with ovarian cancer in *BRCA2*carriers | | |
|  |  |  |  |  |  |  |  |  | Beta^2^ | | SE | *P*-value^3^ | Beta (cm) | SE | *P*-value^3^ | Log Hazard Ratio^4^ | SE | *P*-value^5^ | Log Hazard Ratio^4^ | SE | *P*-value^5^ | Log Hazard Ratio^4^ | SE | *P*-value^5^ |
| rs425277 | 1 | 2069172 | *PRKCZ* | C | T | 0.28 | 0.29 | 0.99 | 0.028 | | 0.003 | 1E-17 | 0.072 | 0.117 | 0.5397 | -0.018 | 0.036 | 0.614 | -0.029 | 0.039 | 0.459 | 0.062 | 0.079 | 0.437 |
| rs9434723 | 1 | 9292282 | *H6PD* | G | A | 0.16 | 0.15 | 0.72 | 0.029 | | 0.004 | 9E-13 | -0.182 | 0.170 | 0.2854 | -0.019 | 0.053 | 0.722 | -0.023 | 0.058 | 0.696 | 0.006 | 0.113 | 0.961 |
| rs10779751 | 1 | 11284336 | *FRAP1* | A | G | 0.72 | 0.72 | 1.00 | -0.021 | | 0.003 | 2E-10 | -0.127 | 0.117 | 0.2766 | -0.014 | 0.036 | 0.687 | -0.033 | 0.039 | 0.395 | 0.118 | 0.082 | 0.149 |
| rs2284746 | 1 | 17306675 | *MFAP2* | C | G | 0.52 | 0.50 | 0.84 | 0.04 | | 0.003 | 1E-40 | 0.422 | 0.113 | 0.0002 | -0.014 | 0.035 | 0.680 | -0.016 | 0.039 | 0.684 | -0.006 | 0.077 | 0.936 |
| rs12137162 | 1 | 19763396 | *CAPZB* | C | A | 0.28 | 0.26 | 0.88 | 0.019 | | 0.003 | 4E-09 | 0.246 | 0.126 | 0.052 | -0.014 | 0.039 | 0.720 | -0.028 | 0.043 | 0.522 | 0.077 | 0.088 | 0.379 |
| rs212524 | 1 | 21583311 | *ECE1* | T | C | 0.60 | 0.59 | 0.93 | 0.021 | | 0.003 | 5E-12 | -0.004 | 0.109 | 0.9699 | -0.029 | 0.034 | 0.384 | -0.045 | 0.037 | 0.229 | 0.072 | 0.075 | 0.334 |
| rs1014987 | 1 | 22498824 | *WNT4* | C | G | 0.25 | 0.23 | 0.94 | 0.02 | | 0.004 | 2E-08 | -0.084 | 0.124 | 0.4964 | 0.028 | 0.038 | 0.467 | 0.030 | 0.042 | 0.476 | 0.012 | 0.079 | 0.883 |
| rs2806561 | 1 | 23504795 | *LUZP1* | A | G | 0.43 | 0.43 | 0.73 | -0.027 | | 0.003 | 2E-20 | -0.014 | 0.124 | 0.9128 | -0.005 | 0.039 | 0.890 | -0.017 | 0.043 | 0.702 | 0.067 | 0.083 | 0.418 |
| rs4601530 | 1 | 25044111 | *CLIC4* | C | T | 0.26 | 0.28 | 0.55 | -0.025 | | 0.003 | 3E-14 | -0.075 | 0.157 | 0.6336 | 0.057 | 0.047 | 0.229 | 0.041 | 0.052 | 0.433 | 0.158 | 0.102 | 0.122 |
| rs17163588 | 1 | 26450009 | *PDIK1L* | C | T | 0.18 | 0.18 | 1 | 0.027 | | 0.004 | 3E-12 | 0.404 | 0.136 | 0.003 | 0.041 | 0.041 | 0.314 | 0.032 | 0.045 | 0.475 | 0.102 | 0.094 | 0.276 |
| rs2219320 | 1 | 26803430 | *HMGN2* | T | C | 0.25 | 0.27 | 1 | -0.022 | | 0.004 | 2E-10 | -0.512 | 0.117 | <.0001 | -0.009 | 0.036 | 0.794 | -0.008 | 0.040 | 0.842 | -0.016 | 0.081 | 0.843 |
| rs12119525 | 1 | 27503662 | *SLC9A1* | T | A | 0.36 | 0.34 | 0.82 | 0.018 | | 0.003 | 5E-09 | -0.108 | 0.121 | 0.3737 | 0.037 | 0.037 | 0.313 | 0.037 | 0.041 | 0.357 | 0.032 | 0.083 | 0.704 |
| rs16834765 | 1 | 32371442 | *PTP4A2* | C | T | 0.06 | 0.05 | 0.82 | 0.045 | | 0.006 | 2E-12 | 0.394 | 0.263 | 0.1336 | 0.077 | 0.080 | 0.337 | 0.087 | 0.088 | 0.318 | 0.009 | 0.188 | 0.961 |
| rs7544462 | 1 | 37962756 | *C1orf149* | A | C | 0.09 | 0.07 | 0.96 | -0.032 | | 0.005 | 2E-09 | -0.282 | 0.207 | 0.174 | -0.087 | 0.067 | 0.194 | -0.050 | 0.073 | 0.491 | -0.363 | 0.162 | 0.025 |
| rs6600365 | 1 | 41556253 | *SCMH1* | C | T | 0.57 | 0.56 | 1.00 | -0.027 | | 0.003 | 2E-20 | -0.179 | 0.106 | 0.0912 | 0.022 | 0.033 | 0.498 | 0.032 | 0.036 | 0.377 | -0.044 | 0.065 | 0.495 |
| rs564914 | 1 | 47915233 | *FOXD2* | A | T | 0.39 | 0.39 | 0.96 | 0.024 | | 0.003 | 2E-15 | 0.157 | 0.108 | 0.1479 | -0.029 | 0.033 | 0.387 | -0.018 | 0.037 | 0.619 | -0.108 | 0.075 | 0.148 |
| rs12855 | 1 | 51440093 | *CDKN2C* | C | T | 0.09 | 0.10 | 0.96 | 0.038 | | 0.005 | 7E-14 | -0.018 | 0.181 | 0.9222 | 0.102 | 0.054 | 0.058 | 0.116 | 0.059 | 0.049 | -0.005 | 0.121 | 0.970 |
| rs17387330 | 1 | 54119578 | *GLIS1* | G | A | 0.32 | 0.27 | 0.67 | 0.017 | | 0.003 | 4E-08 | 0.049 | 0.141 | 0.7302 | -0.003 | 0.044 | 0.946 | -0.028 | 0.049 | 0.565 | 0.159 | 0.092 | 0.083 |
| rs6691924 | 1 | 54954245 | *ACOT11* | C | T | 0.90 | 0.88 | 0.50 | 0.032 | | 0.005 | 2E-10 | -0.486 | 0.228 | 0.0328 | 0.018 | 0.070 | 0.795 | 0.053 | 0.076 | 0.489 | -0.222 | 0.163 | 0.174 |
| rs2815379 | 1 | 67510474 | *SLC35D1* | A | G | 0.71 | 0.71 | 0.76 | 0.018 | | 0.003 | 2E-08 | 0.076 | 0.131 | 0.5616 | 0.044 | 0.041 | 0.277 | 0.053 | 0.045 | 0.237 | -0.015 | 0.091 | 0.865 |
| rs17391694 | 1 | 78623626 | *GIPC2* | C | T | 0.12 | 0.10 | 0.66 | 0.043 | | 0.005 | 4E-16 | 0.699 | 0.206 | 0.0007 | 0.017 | 0.068 | 0.801 | 0.012 | 0.074 | 0.869 | 0.050 | 0.153 | 0.744 |
| rs567401 | 1 | 85988158 | *DDAH1* | C | T | 0.17 | 0.17 | 0.63 | 0.025 | | 0.004 | 2E-09 | 0.198 | 0.171 | 0.2477 | -0.069 | 0.055 | 0.210 | -0.092 | 0.061 | 0.132 | 0.075 | 0.117 | 0.520 |
| rs7551732 | 1 | 89139041 | *PKN2* | T | A | 0.61 | 0.60 | 0.95 | 0.027 | | 0.003 | 6E-20 | 0.084 | 0.108 | 0.4404 | 0.033 | 0.034 | 0.332 | 0.036 | 0.037 | 0.332 | 0.003 | 0.073 | 0.962 |
| rs2811594 | 1 | 93343282 | *FAM69A* | A | G | 0.63 | 0.65 | 0.93 | 0.024 | | 0.003 | 4E-14 | 0.516 | 0.112 | <.0001 | 0.036 | 0.035 | 0.305 | 0.030 | 0.039 | 0.440 | 0.084 | 0.079 | 0.289 |
| rs17113369 | 1 | 95787223 | *RWDD3* | T | C | 0.03 | 0.04 | 0.81 | -0.073 | | 0.013 | 6E-09 | -0.247 | 0.299 | 0.4097 | -0.027 | 0.087 | 0.757 | -0.011 | 0.095 | 0.909 | -0.153 | 0.222 | 0.489 |
| rs7517682 | 1 | 103519589 | *COL11A1* | G | A | 0.56 | 0.56 | 0.98 | -0.023 | | 0.003 | 4E-14 | -0.153 | 0.107 | 0.1517 | 0.085 | 0.033 | 0.009 | 0.091 | 0.036 | 0.012 | 0.048 | 0.073 | 0.506 |
| rs12120956 | 1 | 113202571 | *CAPZA1* | G | A | 0.23 | 0.20 | 0.95 | -0.025 | | 0.004 | 2E-12 | -0.163 | 0.132 | 0.2172 | 0.014 | 0.039 | 0.718 | 0.009 | 0.042 | 0.841 | 0.053 | 0.087 | 0.546 |
| rs1321666 | 1 | 118492052 | *WDR3* | T | C | 0.48 | 0.47 | 0.99 | 0.017 | | 0.003 | 3E-08 | -0.035 | 0.105 | 0.7379 | 0.049 | 0.033 | 0.137 | 0.045 | 0.036 | 0.208 | 0.071 | 0.076 | 0.354 |
| rs9428104 | 1 | 118855587 | *SPAG17* | A | G | 0.75 | 0.75 | 1 | 0.043 | | 0.003 | 3E-36 | 0.454 | 0.119 | 0.0001 | 0.036 | 0.036 | 0.319 | 0.027 | 0.039 | 0.489 | 0.099 | 0.082 | 0.229 |
| rs12144094 | 1 | 120264823 | *PHGDH* | C | G | 0.16 | 0.16 | 0.96 | -0.025 | | 0.004 | 2E-09 | -0.126 | 0.149 | 0.396 | 0.023 | 0.044 | 0.600 | 0.018 | 0.048 | 0.712 | 0.052 | 0.095 | 0.586 |
| rs6658763 | 1 | 146692373 | *FMO5* | C | T | 0.08 | 0.08 | 0.76 | -0.036 | | 0.005 | 4E-11 | 0.345 | 0.218 | 0.1131 | 0.002 | 0.070 | 0.975 | 0.001 | 0.077 | 0.993 | 0.022 | 0.153 | 0.887 |
| rs3767627 | 1 | 149938898 | *OTUD7B* | T | C | 0.16 | 0.14 | 1.00 | -0.036 | | 0.004 | 4E-19 | -0.438 | 0.150 | 0.0035 | 0.080 | 0.045 | 0.074 | 0.085 | 0.049 | 0.081 | 0.038 | 0.104 | 0.717 |
| rs2298265 | 1 | 151259043 | *ZNF687* | C | T | 0.12 | 0.13 | 0.88 | -0.03 | | 0.005 | 8E-11 | -0.190 | 0.171 | 0.2649 | 0.076 | 0.050 | 0.126 | 0.089 | 0.055 | 0.107 | -0.010 | 0.101 | 0.923 |
| rs6688100 | 1 | 160399586 | *VANGL2* | T | C | 0.52 | 0.50 | 0.72 | -0.016 | | 0.003 | 2E-08 | -0.151 | 0.127 | 0.2342 | -0.040 | 0.039 | 0.304 | -0.058 | 0.043 | 0.179 | 0.076 | 0.083 | 0.359 |
| rs4656220 | 1 | 170649277 | *PRRX1* | C | T | 0.39 | 0.37 | 0.76 | 0.021 | | 0.003 | 2E-10 | -0.078 | 0.124 | 0.5269 | -0.005 | 0.038 | 0.894 | -0.021 | 0.043 | 0.617 | 0.098 | 0.081 | 0.225 |
| rs6694089 | 1 | 172083881 | *DNM3* | G | A | 0.28 | 0.27 | 1.00 | 0.039 | | 0.003 | 4E-33 | 0.283 | 0.117 | 0.0154 | -0.019 | 0.034 | 0.589 | -0.025 | 0.038 | 0.510 | 0.025 | 0.078 | 0.746 |
| rs12125882 | 1 | 172141403 | *DNM3* | A | T | 0.43 | 0.42 | 0.90 | 0.027 | | 0.003 | 1E-19 | 0.050 | 0.111 | 0.6515 | -0.059 | 0.034 | 0.087 | -0.064 | 0.038 | 0.088 | -0.018 | 0.075 | 0.815 |
| rs2421992 | 1 | 172241251 | *DNM3* | C | T | 0.70 | 0.70 | 0.67 | 0.024 | | 0.003 | 2E-12 | 0.224 | 0.135 | 0.0981 | -0.002 | 0.041 | 0.965 | -0.022 | 0.045 | 0.628 | 0.130 | 0.095 | 0.168 |
| rs17369123 | 1 | 172355841 | *DNM3* | C | T | 0.19 | 0.16 | 0.98 | 0.031 | | 0.004 | 1E-16 | 0.162 | 0.141 | 0.2505 | -0.008 | 0.046 | 0.856 | 0.015 | 0.051 | 0.760 | -0.167 | 0.097 | 0.084 |
| rs1325596 | 1 | 176794066 | *PAPPA2* | G | A | 0.57 | 0.55 | 0.79 | 0.025 | | 0.003 | 1E-17 | 0.224 | 0.118 | 0.0579 | -0.071 | 0.038 | 0.058 | -0.077 | 0.041 | 0.062 | -0.037 | 0.082 | 0.652 |
| rs4652773 | 1 | 183054827 | *LAMC1* | A | G | 0.54 | 0.53 | 0.96 | -0.024 | | 0.003 | 1E-15 | -0.172 | 0.106 | 0.1055 | 0.030 | 0.034 | 0.381 | 0.032 | 0.038 | 0.394 | 0.004 | 0.073 | 0.953 |
| rs3814333 | 1 | 184007119 | *GLT25D2* | C | T | 0.32 | 0.29 | 1.00 | 0.049 | | 0.003 | 5E-51 | 0.310 | 0.113 | 0.0061 | 0.021 | 0.036 | 0.565 | 0.019 | 0.039 | 0.619 | 0.022 | 0.083 | 0.793 |
| rs2275325 | 1 | 203800735 | *ZC3H11A* | G | C | 0.28 | 0.26 | 0.98 | 0.019 | | 0.003 | 2E-09 | -0.118 | 0.118 | 0.3178 | -0.050 | 0.037 | 0.177 | -0.063 | 0.041 | 0.124 | 0.028 | 0.080 | 0.723 |
| rs10863936 | 1 | 212237798 | *DTL* | G | A | 0.53 | 0.53 | 0.91 | -0.02 | | 0.003 | 1E-11 | 0.032 | 0.109 | 0.7665 | -0.020 | 0.034 | 0.561 | -0.030 | 0.037 | 0.411 | 0.068 | 0.071 | 0.343 |
| rs6540834 | 1 | 214627419 | *PTPN14* | C | T | 0.34 | 0.38 | 0.63 | -0.027 | | 0.003 | 3E-16 | -0.300 | 0.133 | 0.0247 | 0.045 | 0.040 | 0.256 | 0.042 | 0.044 | 0.334 | 0.065 | 0.092 | 0.480 |
| rs10495098 | 1 | 218516310 | *TGFB2* | G | T | 0.41 | 0.41 | 0.84 | 0.019 | | 0.003 | 7E-10 | 0.097 | 0.115 | 0.3999 | 0.014 | 0.036 | 0.695 | -0.001 | 0.039 | 0.979 | 0.125 | 0.077 | 0.107 |
| rs991967 | 1 | 218615451 | *TGFB2* | A | C | 0.28 | 0.30 | 1.00 | 0.034 | | 0.003 | 2E-26 | 0.156 | 0.114 | 0.1734 | -0.030 | 0.035 | 0.397 | -0.035 | 0.039 | 0.366 | -0.001 | 0.075 | 0.992 |
| rs12411277 | 1 | 218975475 | *TGFB2* | G | A | 0.37 | 0.36 | 0.66 | -0.022 | | 0.003 | 2E-13 | -0.181 | 0.134 | 0.1774 | -0.074 | 0.042 | 0.076 | -0.092 | 0.046 | 0.046 | 0.039 | 0.091 | 0.666 |
| rs4428898 | 1 | 219739966 | *SLC30A10* | G | A | 0.46 | 0.46 | 0.89 | -0.023 | | 0.003 | 3E-15 | -0.017 | 0.109 | 0.8778 | 0.024 | 0.033 | 0.458 | 0.027 | 0.036 | 0.460 | 0.010 | 0.075 | 0.893 |
| rs1935157 | 1 | 221317258 | *HLX* | C | G | 0.30 | 0.29 | 0.62 | 0.025 | | 0.003 | 1E-14 | 0.173 | 0.145 | 0.2314 | -0.034 | 0.045 | 0.446 | -0.029 | 0.050 | 0.564 | -0.084 | 0.099 | 0.399 |
| rs6696239 | 1 | 227750068 | *ZNF678* | G | A | 0.19 | 0.17 | 1.00 | -0.038 | | 0.004 | 7E-24 | -0.320 | 0.137 | 0.0193 | -0.011 | 0.043 | 0.800 | -0.009 | 0.047 | 0.847 | -0.018 | 0.093 | 0.845 |
| rs11799609 | 1 | 243618317 | *SDCCAG8* | G | T | 0.16 | 0.16 | 1.00 | 0.026 | | 0.004 | 1E-09 | 0.120 | 0.142 | 0.3969 | -0.059 | 0.046 | 0.200 | -0.058 | 0.051 | 0.257 | -0.073 | 0.098 | 0.457 |
| rs17038954 | 2 | 1645673 | *PXDN* | C | T | 0.06 | 0.07 | 0.52 | 0.044 | | 0.006 | 1E-12 | 0.256 | 0.293 | 0.3816 | 0.003 | 0.100 | 0.975 | -0.022 | 0.111 | 0.842 | 0.184 | 0.192 | 0.338 |
| rs10048625 | 2 | 1775648 | *MYT1L* | C | T | 0.18 | 0.16 | 0.70 | 0.027 | | 0.004 | 6E-11 | 0.244 | 0.173 | 0.1581 | 0.010 | 0.054 | 0.857 | 0.012 | 0.060 | 0.840 | -0.001 | 0.114 | 0.991 |
| rs3885668 | 2 | 10178479 | *KLF11* | C | T | 0.57 | 0.59 | 0.58 | -0.022 | | 0.003 | 8E-13 | -0.032 | 0.139 | 0.8166 | 0.006 | 0.043 | 0.887 | 0.033 | 0.048 | 0.487 | -0.165 | 0.092 | 0.072 |
| rs2345835 | 2 | 18574952 | *RDH14* | T | C | 0.54 | 0.53 | 0.75 | 0.018 | | 0.003 | 2E-09 | 0.121 | 0.120 | 0.3142 | 0.048 | 0.037 | 0.196 | 0.067 | 0.041 | 0.100 | -0.080 | 0.077 | 0.301 |
| rs13006748 | 2 | 20151819 | *WDR35* | G | C | 0.30 | 0.28 | 0.87 | 0.023 | | 0.003 | 6E-11 | -0.093 | 0.123 | 0.4527 | 0.049 | 0.037 | 0.186 | 0.062 | 0.041 | 0.127 | -0.044 | 0.086 | 0.613 |
| rs7561273 | 2 | 24247514 | *LOC38893* | A | G | 0.54 | 0.55 | 1.00 | 0.025 | | 0.003 | 8E-18 | 0.257 | 0.105 | 0.0144 | -0.024 | 0.033 | 0.467 | -0.019 | 0.036 | 0.590 | -0.054 | 0.075 | 0.468 |
| rs2278483 | 2 | 25040082 | *CENPO* | C | T | 0.23 | 0.23 | 0.87 | 0.041 | | 0.003 | 8E-33 | 0.027 | 0.133 | 0.8421 | 0.022 | 0.041 | 0.586 | 0.009 | 0.045 | 0.850 | 0.106 | 0.090 | 0.236 |
| rs2289195 | 2 | 25463483 | *DNMT3A* | G | A | 0.43 | 0.41 | 0.65 | 0.038 | | 0.003 | 2E-37 | 0.422 | 0.133 | 0.0015 | 0.056 | 0.041 | 0.165 | 0.046 | 0.045 | 0.306 | 0.130 | 0.088 | 0.141 |
| rs780094 | 2 | 27741237 | *GCKR* | T | C | 0.61 | 0.57 | 1.00 | 0.021 | | 0.003 | 6E-12 | 0.201 | 0.106 | 0.0574 | 0.022 | 0.032 | 0.488 | 0.018 | 0.035 | 0.608 | 0.050 | 0.072 | 0.486 |
| rs7605699 | 2 | 33315750 | *LTBP1* | G | C | 0.19 | 0.23 | 1.00 | -0.028 | | 0.004 | 2E-12 | -0.226 | 0.125 | 0.0701 | -0.009 | 0.038 | 0.811 | -0.006 | 0.041 | 0.880 | -0.031 | 0.084 | 0.715 |
| rs6714546 | 2 | 33361425 | *LTBP1* | A | G | 0.72 | 0.71 | 0.99 | 0.03 | | 0.003 | 2E-18 | 0.198 | 0.117 | 0.0885 | 0.021 | 0.037 | 0.579 | 0.034 | 0.041 | 0.409 | -0.058 | 0.081 | 0.473 |
| rs6751657 | 2 | 33405151 | *LTBP1* | T | C | 0.53 | 0.51 | 1.00 | 0.024 | | 0.003 | 7E-16 | 0.241 | 0.104 | 0.0205 | -0.019 | 0.031 | 0.541 | -0.040 | 0.034 | 0.250 | 0.123 | 0.068 | 0.072 |
| rs711245 | 2 | 36768875 | *CRIM1* | G | A | 0.33 | 0.32 | 0.89 | -0.024 | | 0.003 | 4E-14 | -0.177 | 0.117 | 0.1305 | 0.055 | 0.037 | 0.134 | 0.039 | 0.041 | 0.345 | 0.179 | 0.081 | 0.028 |
| rs6544089 | 2 | 37758745 | *CDC42EP3* | C | T | 0.39 | 0.36 | 0.79 | 0.02 | | 0.003 | 8E-11 | 0.125 | 0.123 | 0.3086 | 0.059 | 0.037 | 0.114 | 0.066 | 0.041 | 0.107 | 0.014 | 0.084 | 0.869 |
| rs17511102 | 2 | 37960613 | *CDC42EP3* | A | T | 0.09 | 0.06 | 0.55 | 0.053 | | 0.006 | 9E-21 | 0.139 | 0.286 | 0.6264 | 0.048 | 0.088 | 0.585 | 0.071 | 0.096 | 0.457 | -0.129 | 0.204 | 0.525 |
| rs13416119 | 2 | 42462930 | *EML4* | A | G | 0.10 | 0.08 | 0.87 | -0.029 | | 0.005 | 2E-08 | 0.088 | 0.202 | 0.6635 | -0.033 | 0.064 | 0.602 | -0.047 | 0.071 | 0.509 | 0.058 | 0.135 | 0.669 |
| rs9309101 | 2 | 43629612 | *THADA* | A | G | 0.33 | 0.35 | 1.00 | 0.021 | | 0.003 | 6E-11 | 0.065 | 0.108 | 0.5481 | 0.076 | 0.033 | 0.021 | 0.085 | 0.036 | 0.019 | 0.008 | 0.074 | 0.910 |
| rs897080 | 2 | 44774202 | *C2orf34* | C | T | 0.74 | 0.79 | 0.90 | -0.028 | | 0.003 | 2E-16 | -0.204 | 0.135 | 0.1297 | 0.064 | 0.043 | 0.135 | 0.053 | 0.047 | 0.259 | 0.150 | 0.096 | 0.117 |
| rs17032525 | 2 | 44907331 | *C2orf34* | G | A | 0.13 | 0.14 | 0.93 | 0.025 | | 0.005 | 5E-08 | 0.247 | 0.154 | 0.1095 | -0.010 | 0.048 | 0.835 | -0.027 | 0.053 | 0.617 | 0.119 | 0.107 | 0.265 |
| rs12474201 | 2 | 46921285 | *SOCS5* | G | A | 0.36 | 0.36 | 0.97 | 0.028 | | 0.003 | 2E-19 | 0.274 | 0.110 | 0.0124 | 0.002 | 0.034 | 0.950 | 0.013 | 0.037 | 0.719 | -0.079 | 0.075 | 0.295 |
| rs354196 | 2 | 54966407 | *SPTBN1* | A | G | 0.53 | 0.54 | 0.94 | 0.021 | | 0.003 | 6E-12 | 0.009 | 0.108 | 0.9333 | -0.037 | 0.032 | 0.251 | -0.046 | 0.036 | 0.193 | 0.029 | 0.069 | 0.677 |
| rs3791679 | 2 | 56096892 | *EFEMP1* | A | G | 0.23 | 0.24 | 1.00 | -0.06 | | 0.004 | 2E-67 | -0.592 | 0.121 | <.0001 | -0.025 | 0.038 | 0.513 | -0.027 | 0.042 | 0.522 | -0.014 | 0.078 | 0.858 |
| rs2120335 | 2 | 68495002 | *PPP3R1* | G | A | 0.41 | 0.43 | 0.80 | -0.019 | | 0.003 | 8E-10 | -0.184 | 0.118 | 0.1177 | 0.043 | 0.037 | 0.245 | 0.050 | 0.041 | 0.220 | -0.011 | 0.080 | 0.891 |
| rs7568069 | 2 | 71584485 | *ZNF638* | G | A | 0.58 | 0.58 | 1.00 | -0.022 | | 0.003 | 3E-13 | -0.327 | 0.107 | 0.0022 | 0.003 | 0.033 | 0.938 | 0.006 | 0.037 | 0.880 | -0.018 | 0.067 | 0.794 |
| rs11684404 | 2 | 88924622 | *EIF2AK3* | T | C | 0.34 | 0.32 | 0.99 | 0.032 | | 0.003 | 9E-25 | 0.212 | 0.110 | 0.0543 | -0.002 | 0.035 | 0.947 | 0.021 | 0.038 | 0.587 | -0.162 | 0.078 | 0.037 |
| rs11683207 | 2 | 98333290 | *ZAP70* | T | C | 0.20 | 0.14 | 0.82 | -0.023 | | 0.004 | 5E-08 | 0.199 | 0.163 | 0.2213 | -0.022 | 0.053 | 0.682 | -0.044 | 0.059 | 0.452 | 0.125 | 0.111 | 0.260 |
| rs13388725 | 2 | 109047190 | *GCC2* | A | G | 0.41 | 0.39 | 0.67 | 0.018 | | 0.003 | 2E-09 | 0.189 | 0.130 | 0.145 | -0.013 | 0.041 | 0.747 | -0.010 | 0.045 | 0.826 | -0.031 | 0.088 | 0.723 |
| rs2166898 | 2 | 121612659 | *GLI2* | G | A | 0.16 | 0.17 | 0.55 | -0.027 | | 0.004 | 9E-11 | -0.010 | 0.188 | 0.9596 | -0.037 | 0.059 | 0.527 | -0.013 | 0.064 | 0.843 | -0.207 | 0.134 | 0.124 |
| rs7567288 | 2 | 134434824 | *NAP5* | T | C | 0.20 | 0.18 | 0.81 | 0.029 | | 0.004 | 3E-14 | 0.210 | 0.146 | 0.1499 | -0.023 | 0.047 | 0.620 | -0.050 | 0.052 | 0.338 | 0.143 | 0.103 | 0.165 |
| rs4953951 | 2 | 136187345 | *ZRANB3* | C | T | 0.10 | 0.13 | 0.98 | -0.036 | | 0.005 | 1E-11 | -0.183 | 0.167 | 0.272 | -0.002 | 0.048 | 0.961 | 0.020 | 0.052 | 0.699 | -0.179 | 0.116 | 0.124 |
| rs749234 | 2 | 145231349 | *ZEB2* | A | G | 0.68 | 0.69 | 0.74 | -0.017 | | 0.003 | 2E-08 | -0.142 | 0.132 | 0.2814 | 0.062 | 0.041 | 0.129 | 0.050 | 0.045 | 0.262 | 0.138 | 0.088 | 0.117 |
| rs540652 | 2 | 169707428 | *NOSTRIN* | C | T | 0.46 | 0.46 | 0.86 | 0.021 | | 0.003 | 3E-12 | 0.060 | 0.112 | 0.5936 | 0.044 | 0.034 | 0.193 | 0.042 | 0.037 | 0.255 | 0.060 | 0.075 | 0.419 |
| rs12987566 | 2 | 172152646 | *METTL8* | C | T | 0.26 | 0.26 | 0.88 | 0.024 | | 0.003 | 1E-12 | 0.202 | 0.126 | 0.1077 | -0.009 | 0.040 | 0.816 | -0.002 | 0.044 | 0.967 | -0.062 | 0.089 | 0.487 |
| rs6746356 | 2 | 174815898 | *SP3* | A | C | 0.25 | 0.24 | 0.79 | -0.019 | | 0.003 | 1E-08 | 0.070 | 0.137 | 0.6115 | -0.032 | 0.044 | 0.460 | -0.017 | 0.048 | 0.714 | -0.144 | 0.099 | 0.144 |
| rs7567851 | 2 | 178684720 | *PDE11A* | G | C | 0.08 | 0.09 | 0.98 | 0.039 | | 0.006 | 1E-12 | 0.388 | 0.187 | 0.0376 | 0.011 | 0.057 | 0.849 | 0.023 | 0.063 | 0.715 | -0.060 | 0.121 | 0.618 |
| rs833152 | 2 | 183219101 | *PDE1A* | C | A | 0.58 | 0.58 | 0.55 | -0.017 | | 0.003 | 2E-08 | -0.037 | 0.143 | 0.7937 | -0.049 | 0.042 | 0.247 | -0.037 | 0.047 | 0.429 | -0.131 | 0.096 | 0.172 |
| rs12693589 | 2 | 191832662 | *STAT1* | T | C | 0.25 | 0.25 | 1.00 | 0.022 | | 0.003 | 9E-11 | 0.037 | 0.119 | 0.758 | -0.041 | 0.038 | 0.276 | -0.052 | 0.042 | 0.217 | 0.036 | 0.079 | 0.652 |
| rs6435143 | 2 | 203194256 | *NOP5/NOP* | A | C | 0.56 | 0.56 | 0.84 | -0.019 | | 0.003 | 2E-10 | 0.057 | 0.115 | 0.6206 | -0.029 | 0.036 | 0.413 | -0.019 | 0.039 | 0.621 | -0.101 | 0.081 | 0.210 |
| rs4425077 | 2 | 216410516 | *FN1* | G | C | 0.60 | 0.60 | 0.86 | -0.02 | | 0.003 | 1E-10 | -0.160 | 0.115 | 0.166 | 0.008 | 0.036 | 0.814 | 0.011 | 0.039 | 0.773 | -0.004 | 0.076 | 0.963 |
| rs17181956 | 2 | 218146080 | *TNP1* | C | T | 0.13 | 0.10 | 0.86 | -0.028 | | 0.005 | 4E-09 | -0.356 | 0.184 | 0.0535 | -0.106 | 0.058 | 0.069 | -0.132 | 0.065 | 0.042 | 0.056 | 0.116 | 0.627 |
| rs994533 | 2 | 218284278 | *TNS1* | G | C | 0.33 | 0.32 | 1.00 | -0.027 | | 0.003 | 1E-17 | -0.075 | 0.111 | 0.4968 | -0.023 | 0.034 | 0.504 | -0.034 | 0.038 | 0.364 | 0.058 | 0.078 | 0.462 |
| rs1864439 | 2 | 218616633 | *TNS1* | C | T | 0.89 | 0.90 | 0.88 | 0.027 | | 0.005 | 3E-08 | 0.249 | 0.184 | 0.1762 | 0.055 | 0.057 | 0.337 | 0.038 | 0.062 | 0.544 | 0.178 | 0.128 | 0.164 |
| rs992157 | 2 | 219154781 | *PNKD/TMB* | G | A | 0.57 | 0.55 | 1.00 | 0.022 | | 0.003 | 6E-13 | 0.041 | 0.104 | 0.6921 | -0.019 | 0.033 | 0.561 | -0.024 | 0.036 | 0.503 | 0.019 | 0.071 | 0.792 |
| rs2305833 | 2 | 219305404 | *VIL1* | G | C | 0.58 | 0.58 | 0.87 | 0.028 | | 0.003 | 4E-21 | 0.223 | 0.115 | 0.0515 | -0.002 | 0.035 | 0.945 | 0.001 | 0.039 | 0.982 | -0.032 | 0.082 | 0.692 |
| rs12470505 | 2 | 219908369 | *CCDC108* | T | G | 0.10 | 0.10 | 0.97 | -0.048 | | 0.005 | 6E-22 | -0.522 | 0.175 | 0.0029 | -0.143 | 0.055 | 0.009 | -0.137 | 0.060 | 0.023 | -0.189 | 0.129 | 0.143 |
| rs16859517 | 2 | 219949184 | *NHEJ1* | C | T | 0.04 | 0.04 | 0.79 | 0.067 | | 0.008 | 5E-17 | 0.665 | 0.294 | 0.0239 | -0.056 | 0.081 | 0.493 | -0.140 | 0.091 | 0.126 | 0.456 | 0.174 | 0.009 |
| rs6761041 | 2 | 225030129 | *SERPINE2* | T | C | 0.45 | 0.43 | 0.99 | -0.023 | | 0.003 | 3E-15 | 0.091 | 0.106 | 0.3888 | -0.046 | 0.032 | 0.158 | -0.039 | 0.036 | 0.272 | -0.087 | 0.072 | 0.225 |
| rs6733349 | 2 | 232268312 | *B3GNT7* | T | C | 0.34 | 0.37 | 0.52 | -0.022 | | 0.003 | 1E-12 | -0.024 | 0.150 | 0.8714 | -0.016 | 0.045 | 0.725 | -0.011 | 0.050 | 0.822 | -0.043 | 0.102 | 0.671 |
| rs6754426 | 2 | 232322779 | *NCL* | G | A | 0.45 | 0.43 | 0.99 | -0.027 | | 0.003 | 2E-17 | -0.217 | 0.107 | 0.0422 | 0.013 | 0.033 | 0.689 | 0.016 | 0.036 | 0.652 | -0.011 | 0.073 | 0.882 |
| rs4973429 | 2 | 232377818 | *C2orf52* | G | T | 0.34 | 0.36 | 0.99 | -0.029 | | 0.003 | 1E-19 | -0.171 | 0.110 | 0.1219 | -0.015 | 0.034 | 0.656 | 0.000 | 0.037 | 0.993 | -0.120 | 0.075 | 0.109 |
| rs2679184 | 2 | 232779223 | *NPPC* | T | C | 0.23 | 0.26 | 0.53 | -0.031 | | 0.004 | 2E-14 | -0.227 | 0.165 | 0.1676 | -0.038 | 0.050 | 0.453 | -0.046 | 0.055 | 0.401 | 0.021 | 0.112 | 0.850 |
| rs749052 | 2 | 232796610 | *NPPC* | T | C | 0.06 | 0.07 | 0.65 | -0.067 | | 0.006 | 2E-26 | -0.373 | 0.255 | 0.1444 | -0.061 | 0.076 | 0.417 | -0.057 | 0.083 | 0.487 | -0.087 | 0.186 | 0.640 |
| rs11677466 | 2 | 232982257 | *DIS3L2* | A | T | 0.09 | 0.07 | 0.70 | 0.064 | | 0.007 | 3E-23 | 0.799 | 0.233 | 0.0006 | -0.057 | 0.077 | 0.457 | -0.062 | 0.086 | 0.471 | -0.026 | 0.162 | 0.875 |
| rs3116168 | 2 | 232989831 | *DIS3L2* | T | C | 0.73 | 0.68 | 0.65 | 0.039 | | 0.003 | 1E-31 | 0.610 | 0.140 | <.0001 | 0.045 | 0.044 | 0.301 | 0.054 | 0.048 | 0.259 | -0.022 | 0.099 | 0.827 |
| rs2343240 | 2 | 233087483 | *DIS3L2* | T | C | 0.03 | 0.02 | 0.91 | -0.078 | | 0.01 | 3E-15 | 0.340 | 0.351 | 0.3326 | 0.014 | 0.111 | 0.901 | 0.006 | 0.122 | 0.963 | 0.074 | 0.259 | 0.775 |
| rs13393800 | 2 | 233442091 | *EIF4E2* | G | A | 0.29 | 0.26 | 0.55 | 0.027 | | 0.003 | 1E-17 | -0.041 | 0.159 | 0.796 | -0.026 | 0.049 | 0.604 | -0.025 | 0.054 | 0.639 | -0.030 | 0.113 | 0.791 |
| rs4344931 | 2 | 241818527 | *AGXT* | A | C | 0.71 | 0.70 | 0.66 | 0.02 | | 0.003 | 8E-10 | 0.020 | 0.143 | 0.8865 | 0.072 | 0.044 | 0.103 | 0.091 | 0.049 | 0.061 | -0.048 | 0.098 | 0.625 |
| rs11687941 | 2 | 242191410 | *HDLBP* | C | G | 0.25 | 0.26 | 0.96 | -0.025 | | 0.003 | 4E-13 | -0.170 | 0.123 | 0.1673 | -0.079 | 0.037 | 0.031 | -0.105 | 0.040 | 0.010 | 0.106 | 0.082 | 0.196 |
| rs2633761 | 3 | 4728104 | *ITPR1* | G | A | 0.50 | 0.46 | 0.99 | 0.016 | | 0.003 | 3E-08 | 0.189 | 0.104 | 0.0687 | -0.028 | 0.032 | 0.389 | -0.050 | 0.035 | 0.158 | 0.113 | 0.069 | 0.101 |
| rs13078528 | 3 | 11646954 | *VGLL4* | G | A | 0.94 | 0.95 | 0.83 | 0.045 | | 0.006 | 1E-12 | 0.212 | 0.257 | 0.4102 | -0.009 | 0.081 | 0.912 | -0.017 | 0.089 | 0.844 | 0.044 | 0.174 | 0.800 |
| rs2596831 | 3 | 12632652 | *RAF1* | G | C | 0.43 | 0.43 | 0.99 | -0.017 | | 0.003 | 7E-09 | -0.257 | 0.106 | 0.016 | -0.008 | 0.032 | 0.796 | -0.014 | 0.035 | 0.693 | 0.031 | 0.072 | 0.673 |
| rs2597513 | 3 | 13555836 | *HDAC11* | C | T | 0.89 | 0.89 | 0.72 | -0.039 | | 0.005 | 3E-16 | -0.377 | 0.201 | 0.06 | -0.071 | 0.058 | 0.222 | -0.085 | 0.064 | 0.184 | 0.020 | 0.131 | 0.879 |
| rs9816693 | 3 | 38047954 | *VILL* | G | C | 0.17 | 0.15 | 0.84 | 0.031 | | 0.004 | 3E-15 | 0.145 | 0.158 | 0.3595 | 0.029 | 0.050 | 0.567 | 0.045 | 0.055 | 0.405 | -0.082 | 0.110 | 0.455 |
| rs3915129 | 3 | 41243742 | *CTNNB1* | T | G | 0.47 | 0.45 | 1.00 | 0.016 | | 0.003 | 4E-08 | 0.191 | 0.104 | 0.0672 | -0.050 | 0.033 | 0.126 | -0.031 | 0.036 | 0.389 | -0.174 | 0.072 | 0.015 |
| rs13088462 | 3 | 51071713 | *DOCK3* | T | C | 0.06 | 0.05 | 1.00 | 0.059 | | 0.007 | 8E-18 | 0.577 | 0.231 | 0.0125 | 0.082 | 0.075 | 0.277 | 0.120 | 0.081 | 0.138 | -0.226 | 0.179 | 0.208 |
| rs4256170 | 3 | 51192126 | *DOCK3* | G | A | 0.01 | 0.03 | 0.75 | 0.191 | | 0.028 | 1E-11 | 0.630 | 0.335 | 0.0603 | 0.108 | 0.109 | 0.323 | 0.143 | 0.118 | 0.225 | -0.159 | 0.260 | 0.541 |
| rs2240919 | 3 | 52831701 | *ITIH3* | C | G | 0.34 | 0.34 | 1.00 | -0.027 | | 0.003 | 5E-17 | -0.171 | 0.110 | 0.1218 | 0.048 | 0.034 | 0.158 | 0.040 | 0.037 | 0.281 | 0.106 | 0.074 | 0.154 |
| rs2581830 | 3 | 53134098 | *RFT1* | T | C | 0.60 | 0.59 | 1.00 | -0.031 | | 0.003 | 4E-25 | -0.082 | 0.107 | 0.4412 | 0.002 | 0.033 | 0.955 | 0.000 | 0.036 | 0.991 | 0.008 | 0.071 | 0.915 |
| rs2034172 | 3 | 55411763 | *WNT5A* | A | G | 0.68 | 0.66 | 0.75 | 0.018 | | 0.003 | 4E-08 | -0.298 | 0.128 | 0.0199 | -0.045 | 0.039 | 0.255 | -0.028 | 0.043 | 0.509 | -0.165 | 0.090 | 0.065 |
| rs9835332 | 3 | 56667682 | *C3orf63* | G | C | 0.46 | 0.46 | 0.93 | -0.028 | | 0.003 | 4E-22 | -0.396 | 0.108 | 0.0003 | -0.027 | 0.033 | 0.416 | -0.022 | 0.036 | 0.545 | -0.058 | 0.075 | 0.440 |
| rs1658351 | 3 | 58013573 | *FLNB* | C | T | 0.65 | 0.69 | 0.95 | -0.023 | | 0.003 | 1E-12 | -0.298 | 0.114 | 0.0089 | 0.050 | 0.036 | 0.167 | 0.049 | 0.039 | 0.213 | 0.050 | 0.082 | 0.540 |
| rs6794009 | 3 | 61513495 | *PTPRG* | A | G | 0.44 | 0.44 | 0.88 | 0.016 | | 0.003 | 4E-08 | 0.019 | 0.113 | 0.8671 | 0.027 | 0.036 | 0.443 | 0.019 | 0.039 | 0.625 | 0.084 | 0.076 | 0.268 |
| rs17806888 | 3 | 67416322 | *SUCLG2* | T | C | 0.12 | 0.13 | 0.93 | -0.034 | | 0.005 | 3E-12 | -0.344 | 0.163 | 0.0351 | -0.025 | 0.051 | 0.620 | -0.046 | 0.056 | 0.405 | 0.114 | 0.112 | 0.310 |
| rs2175513 | 3 | 68622366 | *FAM19A1* | G | A | 0.57 | 0.55 | 0.86 | -0.017 | | 0.003 | 3E-08 | -0.120 | 0.114 | 0.2929 | 0.012 | 0.035 | 0.731 | 0.004 | 0.038 | 0.919 | 0.067 | 0.075 | 0.366 |
| rs12330322 | 3 | 72455355 | *RYBP* | C | T | 0.22 | 0.22 | 0.83 | -0.034 | | 0.004 | 3E-22 | -0.193 | 0.140 | 0.1686 | 0.071 | 0.042 | 0.089 | 0.108 | 0.046 | 0.020 | -0.176 | 0.092 | 0.055 |
| rs7633464 | 3 | 98715823 | *DCBLD2* | G | A | 0.48 | 0.47 | 1.00 | 0.019 | | 0.003 | 1E-10 | 0.214 | 0.105 | 0.0417 | 0.020 | 0.032 | 0.543 | 0.021 | 0.036 | 0.559 | 0.008 | 0.070 | 0.912 |
| rs9825951 | 3 | 99269921 | *COL8A1* | T | A | 0.65 | 0.63 | 0.84 | -0.022 | | 0.003 | 3E-12 | -0.159 | 0.116 | 0.17 | 0.036 | 0.035 | 0.309 | 0.046 | 0.039 | 0.237 | -0.031 | 0.078 | 0.691 |
| rs1797625 | 3 | 112826415 | *C3orf17* | A | T | 0.36 | 0.36 | 0.91 | 0.019 | | 0.003 | 5E-10 | -0.029 | 0.114 | 0.7957 | -0.011 | 0.036 | 0.751 | -0.011 | 0.039 | 0.784 | -0.014 | 0.083 | 0.863 |
| rs1533269 | 3 | 114214611 | *ZBTB20* | C | A | 0.30 | 0.33 | 0.83 | -0.02 | | 0.003 | 1E-09 | -0.234 | 0.124 | 0.0583 | 0.020 | 0.037 | 0.582 | 0.020 | 0.041 | 0.623 | 0.022 | 0.087 | 0.800 |
| rs1546391 | 3 | 114697457 | *ZBTB20* | C | G | 0.07 | 0.09 | 0.97 | 0.039 | | 0.006 | 7E-11 | 0.349 | 0.193 | 0.0707 | 0.021 | 0.057 | 0.706 | 0.026 | 0.063 | 0.677 | -0.014 | 0.123 | 0.909 |
| rs4974480 | 3 | 134178562 | *ANAPC13* | A | T | 0.68 | 0.68 | 0.95 | 0.026 | | 0.003 | 2E-16 | 0.260 | 0.115 | 0.0241 | 0.007 | 0.036 | 0.856 | 0.011 | 0.040 | 0.791 | -0.017 | 0.078 | 0.824 |
| rs9880211 | 3 | 136107549 | *STAG1* | G | A | 0.25 | 0.25 | 1.00 | -0.03 | | 0.003 | 2E-18 | -0.107 | 0.121 | 0.3757 | 0.019 | 0.037 | 0.613 | 0.022 | 0.041 | 0.601 | -0.001 | 0.082 | 0.988 |
| rs724016 | 3 | 141105570 | *ZBTB38* | A | G | 0.44 | 0.42 | 0.99 | 0.078 | | 0.003 | 3E-158 | 0.357 | 0.106 | 0.0007 | 0.045 | 0.033 | 0.170 | 0.041 | 0.036 | 0.264 | 0.076 | 0.069 | 0.274 |
| rs11714558 | 3 | 141163045 | *ZBTB38* | T | C | 0.03 | 0.02 | 0.52 | -0.134 | | 0.019 | 2E-12 | 0.352 | 0.492 | 0.4741 | 0.014 | 0.146 | 0.926 | 0.054 | 0.159 | 0.733 | -0.276 | 0.359 | 0.442 |
| rs936339 | 3 | 142535505 | *PCOLCE2* | C | T | 0.19 | 0.18 | 0.93 | 0.022 | | 0.004 | 2E-08 | 0.124 | 0.138 | 0.3696 | 0.000 | 0.044 | 0.999 | -0.003 | 0.048 | 0.953 | 0.008 | 0.101 | 0.934 |
| rs4325879 | 3 | 156851984 | *CCNL1* | C | T | 0.27 | 0.27 | 0.97 | -0.021 | | 0.004 | 3E-09 | -0.184 | 0.121 | 0.1271 | -0.021 | 0.035 | 0.550 | -0.025 | 0.038 | 0.523 | 0.008 | 0.080 | 0.921 |
| rs6441170 | 3 | 157806960 | *SHOX2* | T | C | 0.38 | 0.41 | 0.76 | 0.022 | | 0.003 | 1E-12 | 0.194 | 0.120 | 0.1078 | -0.065 | 0.038 | 0.083 | -0.062 | 0.041 | 0.133 | -0.092 | 0.083 | 0.271 |
| rs7652177 | 3 | 171969077 | *FNDC3B* | C | G | 0.51 | 0.52 | 0.73 | 0.038 | | 0.003 | 3E-39 | 0.259 | 0.122 | 0.0334 | 0.057 | 0.038 | 0.133 | 0.069 | 0.042 | 0.099 | -0.031 | 0.081 | 0.707 |
| rs509035 | 3 | 172163449 | *GHSR* | G | A | 0.32 | 0.29 | 0.97 | 0.031 | | 0.003 | 3E-23 | 0.374 | 0.115 | 0.0012 | 0.018 | 0.036 | 0.614 | 0.021 | 0.040 | 0.592 | 0.002 | 0.080 | 0.980 |
| rs9858528 | 3 | 183355405 | *KLHL24* | A | G | 0.26 | 0.27 | 0.98 | -0.022 | | 0.003 | 4E-11 | -0.209 | 0.120 | 0.0821 | -0.013 | 0.036 | 0.716 | -0.013 | 0.040 | 0.736 | -0.014 | 0.083 | 0.862 |
| rs720390 | 3 | 185548683 | *IGF2BP2* | G | A | 0.38 | 0.36 | 0.69 | 0.035 | | 0.003 | 1E-29 | 0.283 | 0.131 | 0.0305 | -0.041 | 0.041 | 0.315 | -0.049 | 0.045 | 0.275 | 0.011 | 0.089 | 0.900 |
| rs2300921 | 3 | 185651001 | *SFRS10* | T | C | 0.42 | 0.41 | 0.98 | 0.018 | | 0.003 | 9E-09 | 0.299 | 0.107 | 0.0052 | -0.013 | 0.033 | 0.688 | -0.012 | 0.036 | 0.743 | -0.016 | 0.073 | 0.827 |
| rs4686904 | 3 | 187438522 | *BCL6* | C | T | 0.65 | 0.66 | 0.99 | -0.021 | | 0.003 | 3E-11 | -0.377 | 0.110 | 0.0006 | 0.007 | 0.034 | 0.846 | -0.013 | 0.037 | 0.716 | 0.151 | 0.079 | 0.056 |
| rs7646824 | 3 | 190815978 | *OSTN* | A | G | 0.11 | 0.11 | 0.96 | -0.026 | | 0.005 | 5E-08 | -0.008 | 0.169 | 0.9611 | -0.021 | 0.050 | 0.679 | -0.043 | 0.057 | 0.446 | 0.094 | 0.103 | 0.358 |
| rs9841435 | 3 | 191111160 | *CCDC50* | A | G | 0.32 | 0.32 | 1.00 | 0.02 | | 0.003 | 2E-10 | -0.142 | 0.112 | 0.2065 | 0.010 | 0.034 | 0.775 | -0.007 | 0.038 | 0.857 | 0.117 | 0.074 | 0.115 |
| rs3958122 | 4 | 1693931 | *SLBP* | C | T | 0.35 | 0.34 | 0.98 | 0.027 | | 0.003 | 3E-18 | 0.253 | 0.111 | 0.0226 | 0.037 | 0.035 | 0.285 | 0.031 | 0.038 | 0.414 | 0.071 | 0.076 | 0.350 |
| rs867245 | 4 | 2218888 | *POLN* | C | G | 0.06 | 0.07 | 1.00 | -0.041 | | 0.006 | 8E-11 | 0.095 | 0.215 | 0.6598 | 0.122 | 0.060 | 0.043 | 0.121 | 0.067 | 0.069 | 0.122 | 0.134 | 0.360 |
| rs6829680 | 4 | 7912333 | *AFAP1* | G | A | 0.44 | 0.46 | 0.91 | 0.017 | | 0.003 | 4E-09 | 0.075 | 0.111 | 0.4978 | 0.029 | 0.035 | 0.404 | 0.030 | 0.039 | 0.438 | 0.030 | 0.073 | 0.685 |
| rs2302580 | 4 | 8608634 | *CPZ* | C | T | 0.42 | 0.44 | 1.00 | -0.029 | | 0.004 | 4E-15 | -0.354 | 0.106 | 0.0008 | 0.044 | 0.032 | 0.173 | 0.053 | 0.036 | 0.140 | -0.009 | 0.073 | 0.904 |
| rs763318 | 4 | 12963574 | *RAB28* | G | A | 0.47 | 0.47 | 0.63 | -0.021 | | 0.003 | 8E-13 | -0.081 | 0.133 | 0.5388 | -0.020 | 0.041 | 0.621 | 0.008 | 0.045 | 0.853 | -0.214 | 0.087 | 0.014 |
| rs4834927 | 4 | 13194091 | *RAB28* | A | G | 0.34 | 0.34 | 0.69 | 0.019 | | 0.003 | 7E-10 | 0.079 | 0.133 | 0.5513 | 0.061 | 0.042 | 0.144 | 0.098 | 0.046 | 0.035 | -0.186 | 0.091 | 0.041 |
| rs7692995 | 4 | 17936634 | *LCORL* | T | C | 0.15 | 0.18 | 0.99 | -0.074 | | 0.004 | 1E-71 | -0.546 | 0.140 | <.0001 | -0.022 | 0.041 | 0.587 | -0.039 | 0.045 | 0.395 | 0.084 | 0.090 | 0.351 |
| rs16994718 | 4 | 38688362 | *KLF3* | C | T | 0.15 | 0.15 | 0.69 | -0.025 | | 0.004 | 2E-09 | -0.178 | 0.179 | 0.3198 | -0.008 | 0.053 | 0.879 | 0.007 | 0.059 | 0.910 | -0.117 | 0.118 | 0.324 |
| rs2306596 | 4 | 39343940 | *RFC1* | C | A | 0.52 | 0.53 | 0.97 | 0.019 | | 0.003 | 8E-11 | 0.229 | 0.106 | 0.0308 | -0.008 | 0.033 | 0.811 | -0.018 | 0.036 | 0.611 | 0.064 | 0.075 | 0.392 |
| rs1996422 | 4 | 48687351 | *FRYL* | A | G | 0.28 | 0.26 | 1.00 | 0.022 | | 0.003 | 3E-11 | 0.023 | 0.117 | 0.8419 | 0.034 | 0.038 | 0.362 | 0.048 | 0.042 | 0.244 | -0.065 | 0.081 | 0.427 |
| rs13113518 | 4 | 56399648 | *CLOCK* | T | C | 0.36 | 0.37 | 0.99 | 0.018 | | 0.003 | 8E-09 | 0.081 | 0.108 | 0.4559 | 0.081 | 0.033 | 0.014 | 0.075 | 0.036 | 0.038 | 0.125 | 0.072 | 0.080 |
| rs17081935 | 4 | 57823476 | *C4orf14* | C | T | 0.19 | 0.19 | 0.94 | 0.031 | | 0.004 | 7E-17 | 0.457 | 0.137 | 0.0009 | 0.063 | 0.043 | 0.142 | 0.046 | 0.048 | 0.340 | 0.180 | 0.092 | 0.050 |
| rs9993613 | 4 | 73476014 | *ADAMTS3* | T | G | 0.53 | 0.55 | 0.72 | -0.03 | | 0.003 | 5E-24 | -0.087 | 0.125 | 0.4855 | 0.011 | 0.039 | 0.780 | 0.007 | 0.043 | 0.864 | 0.039 | 0.081 | 0.632 |
| rs17556750 | 4 | 82155568 | *PRKG2* | C | A | 0.31 | 0.28 | 0.92 | 0.046 | | 0.003 | 8E-48 | 0.371 | 0.121 | 0.0021 | 0.005 | 0.038 | 0.888 | -0.019 | 0.042 | 0.651 | 0.163 | 0.082 | 0.046 |
| rs2167645 | 4 | 82184049 | *PRKG2* | C | T | 0.04 | 0.03 | 0.52 | 0.139 | | 0.021 | 8E-11 | 0.607 | 0.453 | 0.1796 | -0.076 | 0.136 | 0.576 | -0.139 | 0.150 | 0.354 | 0.334 | 0.306 | 0.276 |
| rs17499117 | 4 | 82204091 | *PRKG2* | A | G | 0.04 | 0.05 | 0.64 | 0.14 | | 0.021 | 2E-11 | 0.681 | 0.297 | 0.0218 | -0.029 | 0.090 | 0.747 | -0.057 | 0.099 | 0.568 | 0.148 | 0.199 | 0.458 |
| rs6813055 | 4 | 88630031 | *DMP1* | A | T | 0.50 | 0.46 | 0.99 | -0.017 | | 0.003 | 2E-08 | 0.210 | 0.106 | 0.0475 | -0.004 | 0.033 | 0.896 | 0.007 | 0.037 | 0.857 | -0.081 | 0.073 | 0.271 |
| rs12639764 | 4 | 106216205 | *TET2* | T | C | 0.38 | 0.37 | 1.00 | -0.027 | | 0.003 | 2E-19 | 0.025 | 0.107 | 0.8123 | -0.010 | 0.034 | 0.763 | -0.004 | 0.038 | 0.926 | -0.057 | 0.074 | 0.439 |
| rs1562975 | 4 | 109408608 | *RPL34* | G | A | 0.30 | 0.26 | 0.76 | 0.025 | | 0.003 | 6E-15 | 0.068 | 0.138 | 0.621 | 0.008 | 0.041 | 0.840 | 0.026 | 0.045 | 0.559 | -0.112 | 0.095 | 0.239 |
| rs7659107 | 4 | 114742249 | *CAMK2D* | A | G | 0.23 | 0.21 | 0.66 | 0.024 | | 0.004 | 9E-12 | 0.388 | 0.155 | 0.0126 | -0.006 | 0.049 | 0.902 | -0.002 | 0.055 | 0.970 | -0.039 | 0.104 | 0.706 |
| rs6838153 | 4 | 122720999 | *EXOSC9* | A | G | 0.34 | 0.33 | 0.99 | 0.022 | | 0.003 | 3E-12 | 0.090 | 0.112 | 0.4201 | -0.072 | 0.034 | 0.033 | -0.061 | 0.037 | 0.100 | -0.154 | 0.077 | 0.044 |
| rs12513181 | 4 | 123835656 | *NUDT6* | C | A | 0.74 | 0.73 | 1.00 | -0.02 | | 0.003 | 3E-09 | -0.028 | 0.118 | 0.8098 | -0.045 | 0.037 | 0.219 | -0.060 | 0.040 | 0.138 | 0.056 | 0.083 | 0.496 |
| rs11100790 | 4 | 144442611 | *SMARCA5* | T | C | 0.20 | 0.25 | 0.59 | 0.025 | | 0.004 | 8E-12 | 0.033 | 0.156 | 0.8344 | -0.036 | 0.048 | 0.446 | -0.038 | 0.052 | 0.462 | -0.019 | 0.107 | 0.857 |
| rs7654571 | 4 | 145321006 | *HHIP* | A | G | 0.77 | 0.75 | 0.80 | -0.022 | | 0.004 | 4E-09 | 0.237 | 0.135 | 0.0785 | -0.044 | 0.040 | 0.276 | -0.044 | 0.044 | 0.320 | -0.051 | 0.091 | 0.573 |
| rs6845999 | 4 | 145565826 | *HHIP* | C | T | 0.44 | 0.43 | 0.82 | 0.051 | | 0.003 | 5E-67 | 0.314 | 0.118 | 0.0078 | -0.024 | 0.036 | 0.512 | -0.029 | 0.040 | 0.459 | 0.012 | 0.082 | 0.882 |
| rs1812175 | 4 | 145574844 | *HHIP* | A | G | 0.84 | 0.83 | 1.00 | 0.079 | | 0.004 | 2E-86 | 0.484 | 0.141 | 0.0006 | 0.013 | 0.044 | 0.766 | 0.000 | 0.048 | 0.993 | 0.107 | 0.097 | 0.269 |
| rs17777628 | 4 | 145592686 | *HHIP* | G | A | 0.04 | 0.04 | 0.97 | -0.053 | | 0.008 | 8E-12 | -0.928 | 0.275 | 0.0007 | -0.007 | 0.080 | 0.933 | 0.004 | 0.087 | 0.966 | -0.080 | 0.193 | 0.680 |
| rs4240326 | 4 | 145839264 | *ANAPC10* | A | G | 0.54 | 0.55 | 0.89 | -0.04 | | 0.003 | 3E-43 | -0.345 | 0.111 | 0.0019 | -0.018 | 0.035 | 0.606 | 0.001 | 0.039 | 0.974 | -0.149 | 0.077 | 0.053 |
| rs996743 | 4 | 146128884 | *OTUD4* | A | G | 0.08 | 0.09 | 0.83 | -0.063 | | 0.007 | 2E-22 | -0.240 | 0.201 | 0.2329 | 0.036 | 0.061 | 0.550 | 0.044 | 0.065 | 0.496 | -0.023 | 0.153 | 0.882 |
| rs13133465 | 4 | 146192618 | *OTUD4* | C | G | 0.06 | 0.05 | 0.65 | -0.045 | | 0.007 | 4E-12 | -0.390 | 0.282 | 0.1666 | -0.047 | 0.090 | 0.601 | -0.032 | 0.098 | 0.745 | -0.183 | 0.209 | 0.382 |
| rs13150868 | 4 | 152180671 | *ESSPL* | G | T | 0.44 | 0.46 | 0.55 | 0.017 | | 0.003 | 2E-08 | 0.054 | 0.142 | 0.7009 | -0.032 | 0.043 | 0.461 | -0.031 | 0.047 | 0.520 | -0.032 | 0.094 | 0.731 |
| rs955748 | 4 | 184215675 | *WWC2* | A | G | 0.75 | 0.76 | 1.00 | 0.028 | | 0.003 | 3E-16 | 0.050 | 0.122 | 0.6828 | 0.041 | 0.037 | 0.265 | 0.034 | 0.041 | 0.398 | 0.082 | 0.080 | 0.304 |
| rs17410035 | 5 | 31541142 | *C5orf22* | G | T | 0.33 | 0.33 | 1.00 | 0.019 | | 0.003 | 9E-10 | 0.097 | 0.111 | 0.3813 | -0.042 | 0.034 | 0.217 | -0.045 | 0.037 | 0.221 | -0.017 | 0.078 | 0.830 |
| rs7731703 | 5 | 32694942 | *NPR3* | C | T | 0.32 | 0.33 | 1.00 | -0.03 | | 0.003 | 4E-18 | -0.227 | 0.112 | 0.0426 | -0.028 | 0.034 | 0.399 | -0.026 | 0.037 | 0.485 | -0.042 | 0.072 | 0.563 |
| rs3811958 | 5 | 32772043 | *NPR3* | A | G | 0.26 | 0.25 | 0.56 | 0.027 | | 0.003 | 3E-16 | 0.021 | 0.160 | 0.8977 | 0.028 | 0.048 | 0.557 | 0.026 | 0.053 | 0.623 | 0.043 | 0.106 | 0.686 |
| rs9292468 | 5 | 32819073 | *C5orf23* | T | C | 0.60 | 0.59 | 0.75 | -0.036 | | 0.003 | 2E-33 | -0.198 | 0.124 | 0.11 | -0.005 | 0.038 | 0.893 | -0.008 | 0.041 | 0.846 | 0.017 | 0.086 | 0.844 |
| rs11745439 | 5 | 33230034 | *TARS* | A | G | 0.72 | 0.71 | 0.84 | 0.026 | | 0.003 | 8E-16 | -0.001 | 0.127 | 0.9935 | 0.005 | 0.040 | 0.899 | 0.028 | 0.044 | 0.534 | -0.139 | 0.084 | 0.098 |
| rs301901 | 5 | 37046626 | *NIPBL* | A | G | 0.44 | 0.45 | 0.93 | -0.024 | | 0.003 | 4E-16 | -0.178 | 0.109 | 0.1008 | -0.019 | 0.034 | 0.590 | -0.027 | 0.038 | 0.472 | 0.035 | 0.073 | 0.629 |
| rs3812040 | 5 | 39426020 | *DAB2* | T | C | 0.28 | 0.27 | 0.90 | -0.024 | | 0.003 | 2E-13 | 0.043 | 0.122 | 0.7228 | 0.037 | 0.038 | 0.331 | 0.035 | 0.042 | 0.411 | 0.059 | 0.086 | 0.491 |
| rs17574650 | 5 | 42436916 | *GHR* | A | C | 0.11 | 0.11 | 0.74 | 0.038 | | 0.005 | 2E-12 | 0.124 | 0.192 | 0.5172 | -0.005 | 0.061 | 0.935 | -0.006 | 0.066 | 0.926 | 0.010 | 0.138 | 0.945 |
| rs2961830 | 5 | 50454732 | *ISL1* | A | T | 0.65 | 0.63 | 0.84 | -0.02 | | 0.003 | 2E-10 | -0.015 | 0.119 | 0.8968 | -0.021 | 0.036 | 0.557 | -0.039 | 0.040 | 0.322 | 0.104 | 0.081 | 0.199 |
| rs7716219 | 5 | 54955071 | *SLC38A9* | T | C | 0.69 | 0.70 | 0.97 | -0.03 | | 0.003 | 7E-22 | -0.304 | 0.115 | 0.0084 | -0.008 | 0.035 | 0.828 | 0.002 | 0.039 | 0.967 | -0.065 | 0.077 | 0.401 |
| rs2662027 | 5 | 56254485 | *MIER3* | G | T | 0.10 | 0.10 | 1.00 | -0.033 | | 0.005 | 6E-12 | -0.229 | 0.170 | 0.1782 | 0.078 | 0.051 | 0.124 | 0.083 | 0.056 | 0.137 | 0.048 | 0.113 | 0.672 |
| rs7727731 | 5 | 64674446 | *ADAMTS6* | C | T | 0.11 | 0.12 | 0.67 | 0.033 | | 0.005 | 1E-11 | 0.066 | 0.189 | 0.7277 | 0.005 | 0.061 | 0.938 | -0.018 | 0.067 | 0.793 | 0.150 | 0.123 | 0.222 |
| rs9291926 | 5 | 67599656 | *PIK3R1* | T | G | 0.51 | 0.51 | 0.99 | -0.019 | | 0.003 | 3E-10 | -0.164 | 0.104 | 0.1146 | -0.008 | 0.033 | 0.798 | -0.007 | 0.036 | 0.850 | -0.017 | 0.070 | 0.814 |
| rs34651 | 5 | 72144005 | *TNPO1* | C | T | 0.92 | 0.93 | 0.86 | -0.041 | | 0.006 | 2E-12 | -0.595 | 0.215 | 0.0057 | 0.000 | 0.070 | 1.000 | -0.020 | 0.076 | 0.790 | 0.156 | 0.164 | 0.342 |
| rs820848 | 5 | 73964660 | *HEXB* | A | G | 0.29 | 0.29 | 1.00 | 0.021 | | 0.004 | 3E-09 | 0.098 | 0.115 | 0.3948 | 0.033 | 0.035 | 0.346 | 0.025 | 0.039 | 0.515 | 0.080 | 0.076 | 0.295 |
| rs7712162 | 5 | 78945171 | *PAPD4* | C | T | 0.19 | 0.17 | 0.94 | 0.023 | | 0.004 | 5E-10 | 0.255 | 0.143 | 0.0737 | 0.017 | 0.044 | 0.701 | 0.001 | 0.049 | 0.984 | 0.118 | 0.094 | 0.207 |
| rs32855 | 5 | 79836192 | *FAM151B* | G | A | 0.78 | 0.79 | 0.96 | 0.024 | | 0.004 | 6E-11 | 0.047 | 0.132 | 0.723 | 0.020 | 0.039 | 0.610 | 0.005 | 0.043 | 0.916 | 0.132 | 0.095 | 0.164 |
| rs6894139 | 5 | 88327782 | *MEF2C* | T | G | 0.44 | 0.48 | 0.93 | -0.03 | | 0.003 | 6E-24 | -0.249 | 0.107 | 0.0201 | -0.008 | 0.033 | 0.813 | -0.003 | 0.037 | 0.943 | -0.047 | 0.076 | 0.534 |
| rs12186664 | 5 | 95630225 | *PCSK1* | A | T | 0.32 | 0.31 | 0.70 | 0.021 | | 0.003 | 3E-11 | 0.256 | 0.134 | 0.0571 | -0.009 | 0.041 | 0.830 | -0.016 | 0.045 | 0.721 | 0.043 | 0.092 | 0.645 |
| rs6594336 | 5 | 108073085 | *FER* | T | C | 0.56 | 0.55 | 0.97 | 0.017 | | 0.003 | 1E-08 | 0.050 | 0.107 | 0.6431 | 0.037 | 0.033 | 0.264 | 0.049 | 0.036 | 0.174 | -0.046 | 0.076 | 0.548 |
| rs13177718 | 5 | 108113344 | *FER* | C | T | 0.08 | 0.08 | 0.97 | -0.043 | | 0.006 | 3E-13 | -0.409 | 0.203 | 0.0436 | 0.111 | 0.060 | 0.065 | 0.145 | 0.066 | 0.028 | -0.100 | 0.132 | 0.449 |
| rs1582931 | 5 | 122657199 | *CCDC100* | G | A | 0.47 | 0.47 | 0.91 | -0.028 | | 0.003 | 3E-20 | -0.153 | 0.110 | 0.1637 | -0.027 | 0.034 | 0.433 | -0.021 | 0.038 | 0.569 | -0.062 | 0.078 | 0.431 |
| rs6887276 | 5 | 127378294 | *SLC12A2* | C | G | 0.45 | 0.47 | 0.82 | 0.018 | | 0.003 | 1E-09 | 0.118 | 0.114 | 0.3009 | 0.067 | 0.035 | 0.057 | 0.061 | 0.039 | 0.115 | 0.106 | 0.079 | 0.177 |
| rs26024 | 5 | 127696022 | *FBN2* | A | C | 0.34 | 0.34 | 1.00 | 0.023 | | 0.003 | 3E-14 | 0.226 | 0.109 | 0.0377 | -0.087 | 0.034 | 0.011 | -0.116 | 0.038 | 0.002 | 0.088 | 0.071 | 0.212 |
| rs39623 | 5 | 129054621 | *ADAMTS19* | A | T | 0.92 | 0.93 | 0.94 | -0.034 | | 0.005 | 3E-10 | -0.232 | 0.210 | 0.2702 | 0.030 | 0.064 | 0.640 | 0.028 | 0.070 | 0.687 | 0.056 | 0.146 | 0.700 |
| rs7701414 | 5 | 131585958 | *PDLIM4* | A | G | 0.44 | 0.46 | 1.00 | 0.037 | | 0.003 | 1E-34 | 0.221 | 0.105 | 0.0355 | 0.001 | 0.033 | 0.985 | -0.004 | 0.036 | 0.917 | 0.026 | 0.071 | 0.711 |
| rs526896 | 5 | 134356705 | *PITX1* | T | G | 0.28 | 0.28 | 0.84 | -0.036 | | 0.003 | 2E-25 | -0.114 | 0.128 | 0.3746 | -0.054 | 0.039 | 0.161 | -0.050 | 0.043 | 0.243 | -0.082 | 0.084 | 0.330 |
| rs9327705 | 5 | 134510303 | *PITX1* | G | A | 0.23 | 0.23 | 1.00 | 0.02 | | 0.004 | 2E-08 | -0.020 | 0.125 | 0.8706 | 0.019 | 0.038 | 0.615 | 0.013 | 0.042 | 0.758 | 0.057 | 0.081 | 0.478 |
| rs165189 | 5 | 139145747 | *PSD2* | A | G | 0.15 | 0.14 | 0.64 | 0.029 | | 0.005 | 2E-10 | 0.193 | 0.190 | 0.3094 | -0.025 | 0.058 | 0.666 | -0.030 | 0.064 | 0.637 | 0.004 | 0.124 | 0.974 |
| rs4624820 | 5 | 141681788 | *SPRY4* | G | A | 0.52 | 0.55 | 1.00 | 0.018 | | 0.003 | 1E-09 | -0.037 | 0.103 | 0.7202 | -0.031 | 0.032 | 0.339 | -0.046 | 0.036 | 0.194 | 0.068 | 0.072 | 0.342 |
| rs2974438 | 5 | 168250903 | *SLIT3* | G | A | 0.20 | 0.22 | 0.77 | -0.037 | | 0.004 | 4E-24 | -0.214 | 0.144 | 0.1362 | 0.003 | 0.047 | 0.942 | -0.012 | 0.052 | 0.811 | 0.102 | 0.093 | 0.269 |
| rs4620037 | 5 | 170875097 | *FGF18* | A | C | 0.20 | 0.23 | 0.66 | -0.032 | | 0.004 | 1E-18 | -0.139 | 0.156 | 0.3701 | -0.034 | 0.048 | 0.472 | -0.028 | 0.053 | 0.599 | -0.082 | 0.107 | 0.444 |
| rs1529701 | 5 | 171000977 | *FGF18* | T | C | 0.70 | 0.71 | 0.95 | 0.021 | | 0.003 | 5E-10 | 0.187 | 0.117 | 0.1093 | 0.012 | 0.037 | 0.740 | 0.018 | 0.040 | 0.654 | -0.027 | 0.084 | 0.751 |
| rs33852 | 5 | 171189571 | *FBXW11* | A | G | 0.33 | 0.32 | 0.93 | 0.03 | | 0.003 | 2E-21 | 0.244 | 0.115 | 0.0347 | 0.016 | 0.035 | 0.640 | 0.016 | 0.038 | 0.681 | 0.030 | 0.080 | 0.707 |
| rs12153391 | 5 | 171203438 | *FBXW11* | C | A | 0.26 | 0.25 | 0.92 | -0.033 | | 0.004 | 2E-21 | -0.059 | 0.124 | 0.6375 | 0.018 | 0.039 | 0.645 | -0.001 | 0.043 | 0.979 | 0.141 | 0.082 | 0.086 |
| rs7733195 | 5 | 172994624 | *FAM44B* | G | A | 0.36 | 0.37 | 1.00 | -0.029 | | 0.003 | 3E-21 | -0.259 | 0.109 | 0.0175 | 0.026 | 0.034 | 0.439 | 0.053 | 0.038 | 0.157 | -0.158 | 0.077 | 0.041 |
| rs422421 | 5 | 176517326 | *FGFR4* | T | C | 0.78 | 0.77 | 1.00 | 0.034 | | 0.004 | 2E-20 | 0.264 | 0.126 | 0.0354 | 0.013 | 0.038 | 0.730 | 0.016 | 0.042 | 0.702 | -0.005 | 0.087 | 0.958 |
| rs11950938 | 5 | 176576293 | *NSD1* | C | G | 0.03 | 0.02 | 0.90 | 0.061 | | 0.009 | 5E-11 | 0.162 | 0.346 | 0.6395 | 0.011 | 0.108 | 0.919 | 0.071 | 0.116 | 0.538 | -0.483 | 0.274 | 0.077 |
| rs12055154 | 5 | 176675423 | *NSD1* | A | G | 0.03 | 0.02 | 0.83 | -0.059 | | 0.01 | 2E-09 | -0.918 | 0.391 | 0.0188 | 0.022 | 0.120 | 0.852 | 0.048 | 0.133 | 0.720 | -0.118 | 0.270 | 0.662 |
| rs11750568 | 5 | 178535713 | *ADAMTS2* | A | G | 0.67 | 0.70 | 0.79 | -0.02 | | 0.003 | 6E-11 | -0.030 | 0.127 | 0.8139 | -0.066 | 0.040 | 0.096 | -0.080 | 0.044 | 0.065 | 0.035 | 0.086 | 0.686 |
| rs6879260 | 5 | 179731014 | *GFPT2* | T | C | 0.61 | 0.63 | 0.57 | 0.027 | | 0.003 | 2E-18 | 0.124 | 0.144 | 0.3906 | -0.062 | 0.044 | 0.159 | -0.048 | 0.049 | 0.322 | -0.153 | 0.097 | 0.115 |
| rs932445 | 6 | 2167225 | *GMDS* | T | C | 0.41 | 0.36 | 0.69 | -0.018 | | 0.003 | 6E-09 | -0.348 | 0.133 | 0.0087 | -0.036 | 0.041 | 0.370 | -0.033 | 0.045 | 0.461 | -0.066 | 0.089 | 0.462 |
| rs17603945 | 6 | 7213016 | *RREB1* | G | A | 0.20 | 0.17 | 0.99 | 0.03 | | 0.004 | 1E-15 | 0.374 | 0.138 | 0.0068 | 0.006 | 0.044 | 0.888 | 0.009 | 0.048 | 0.851 | -0.020 | 0.096 | 0.838 |
| rs9392918 | 6 | 7708631 | *BMP6* | T | C | 0.47 | 0.46 | 0.99 | 0.038 | | 0.003 | 4E-38 | 0.123 | 0.105 | 0.2392 | 0.010 | 0.033 | 0.763 | 0.016 | 0.036 | 0.652 | -0.040 | 0.072 | 0.575 |
| rs17330192 | 6 | 17589375 | *FAM8A1* | T | C | 0.28 | 0.21 | 0.78 | 0.019 | | 0.003 | 2E-08 | 0.032 | 0.143 | 0.824 | -0.059 | 0.045 | 0.186 | -0.062 | 0.049 | 0.211 | -0.047 | 0.100 | 0.641 |
| rs4141885 | 6 | 26157481 | *HIST1H1E* | A | T | 0.08 | 0.12 | 0.70 | -0.073 | | 0.008 | 8E-22 | -0.638 | 0.194 | 0.001 | 0.006 | 0.055 | 0.910 | 0.025 | 0.060 | 0.676 | -0.129 | 0.126 | 0.306 |
| rs806794 | 6 | 26200677 | *HIST1H2B* | A | G | 0.29 | 0.31 | 0.95 | -0.06 | | 0.003 | 5E-74 | -0.309 | 0.116 | 0.0079 | 0.035 | 0.035 | 0.307 | 0.039 | 0.038 | 0.304 | 0.009 | 0.077 | 0.906 |
| rs1265097 | 6 | 31106459 | *PSORS1C1* | C | A | 0.12 | 0.13 | 1.00 | -0.059 | | 0.005 | 2E-32 | -0.351 | 0.162 | 0.0307 | -0.020 | 0.047 | 0.671 | -0.018 | 0.051 | 0.725 | -0.026 | 0.106 | 0.810 |
| rs2857693 | 6 | 31588384 | *BAT2* | G | T | 0.38 | 0.44 | 1.00 | -0.034 | | 0.003 | 3E-29 | -0.505 | 0.105 | <.0001 | -0.013 | 0.033 | 0.702 | -0.023 | 0.037 | 0.525 | 0.066 | 0.075 | 0.381 |
| rs12204421 | 6 | 33628863 | *ITPR3* | A | G | 0.26 | 0.27 | 0.95 | -0.027 | | 0.003 | 1E-14 | -0.072 | 0.121 | 0.5552 | 0.062 | 0.037 | 0.097 | 0.040 | 0.041 | 0.328 | 0.213 | 0.082 | 0.010 |
| rs3957165 | 6 | 33784864 | *MLN* | C | T | 0.81 | 0.80 | 0.91 | 0.022 | | 0.004 | 2E-08 | -0.093 | 0.135 | 0.4937 | 0.014 | 0.042 | 0.745 | 0.002 | 0.046 | 0.962 | 0.103 | 0.097 | 0.290 |
| rs12214804 | 6 | 34188866 | *HMGA1* | C | T | 0.92 | 0.90 | 0.98 | -0.084 | | 0.006 | 2E-49 | -0.462 | 0.181 | 0.0107 | -0.017 | 0.055 | 0.755 | -0.022 | 0.062 | 0.719 | 0.015 | 0.116 | 0.896 |
| rs3800461 | 6 | 34616322 | *C6orf106* | G | C | 0.13 | 0.11 | 1.00 | 0.053 | | 0.005 | 9E-33 | 0.647 | 0.163 | <.0001 | 0.004 | 0.052 | 0.945 | -0.003 | 0.057 | 0.964 | 0.054 | 0.116 | 0.645 |
| rs6919534 | 6 | 35246903 | *ZNF76* | G | A | 0.87 | 0.85 | 1.00 | 0.05 | | 0.004 | 8E-31 | 0.140 | 0.144 | 0.3307 | -0.007 | 0.045 | 0.873 | -0.014 | 0.049 | 0.777 | 0.039 | 0.100 | 0.701 |
| rs6899744 | 6 | 35286295 | *DEF6* | G | T | 0.02 | 0.02 | 0.97 | -0.132 | | 0.014 | 2E-22 | 0.068 | 0.420 | 0.8712 | -0.119 | 0.129 | 0.353 | -0.111 | 0.142 | 0.432 | -0.167 | 0.292 | 0.566 |
| rs4713902 | 6 | 35614026 | *FKBP5* | T | C | 0.27 | 0.29 | 1.00 | -0.027 | | 0.004 | 9E-15 | 0.121 | 0.116 | 0.2969 | 0.061 | 0.034 | 0.077 | 0.056 | 0.038 | 0.140 | 0.093 | 0.074 | 0.206 |
| rs16895130 | 6 | 41924931 | *CCND3* | A | G | 0.28 | 0.29 | 0.88 | 0.023 | | 0.003 | 4E-12 | -0.204 | 0.125 | 0.1023 | 0.018 | 0.037 | 0.631 | 0.027 | 0.041 | 0.513 | -0.039 | 0.085 | 0.650 |
| rs10948222 | 6 | 45244415 | *SUPT3H* | T | C | 0.58 | 0.42 | 0.98 | 0.031 | | 0.003 | 1E-20 | 0.131 | 0.106 | 0.219 | -0.034 | 0.033 | 0.295 | -0.031 | 0.036 | 0.381 | -0.056 | 0.072 | 0.441 |
| rs9395264 | 6 | 47475022 | *CD2AP* | T | G | 0.68 | 0.70 | 0.93 | 0.02 | | 0.003 | 2E-10 | 0.012 | 0.119 | 0.9225 | -0.044 | 0.037 | 0.231 | -0.040 | 0.040 | 0.325 | -0.075 | 0.078 | 0.336 |
| rs12190423 | 6 | 72202711 | *OGFRL1* | G | C | 0.38 | 0.38 | 0.86 | -0.017 | | 0.003 | 3E-08 | -0.139 | 0.115 | 0.2279 | 0.023 | 0.035 | 0.514 | 0.027 | 0.039 | 0.489 | -0.008 | 0.076 | 0.915 |
| rs12209223 | 6 | 76164589 | *FILIP1* | C | A | 0.12 | 0.09 | 0.75 | 0.051 | | 0.005 | 5E-25 | 0.278 | 0.204 | 0.1725 | -0.006 | 0.063 | 0.927 | -0.044 | 0.070 | 0.530 | 0.237 | 0.129 | 0.065 |
| rs6903448 | 6 | 76173832 | *FILIP1* | C | T | 0.16 | 0.16 | 0.80 | -0.035 | | 0.004 | 9E-18 | -0.197 | 0.159 | 0.215 | 0.010 | 0.051 | 0.843 | 0.029 | 0.056 | 0.613 | -0.117 | 0.109 | 0.283 |
| rs648831 | 6 | 80956208 | *BCKDHB* | C | T | 0.50 | 0.51 | 0.94 | 0.031 | | 0.003 | 3E-26 | 0.297 | 0.106 | 0.0053 | -0.049 | 0.034 | 0.149 | -0.061 | 0.038 | 0.105 | 0.028 | 0.073 | 0.700 |
| rs1341278 | 6 | 81038921 | *BCKDHB* | T | G | 0.06 | 0.05 | 1.00 | 0.056 | | 0.006 | 6E-18 | 0.247 | 0.226 | 0.2762 | -0.112 | 0.073 | 0.126 | -0.143 | 0.081 | 0.079 | 0.081 | 0.152 | 0.592 |
| rs9443804 | 6 | 81315597 | *BCKDHB* | A | G | 0.44 | 0.46 | 0.81 | 0.024 | | 0.003 | 4E-15 | 0.151 | 0.115 | 0.1889 | 0.052 | 0.035 | 0.141 | 0.063 | 0.039 | 0.104 | -0.026 | 0.078 | 0.740 |
| rs310421 | 6 | 81792063 | *FAM46A* | G | T | 0.54 | 0.55 | 0.83 | 0.032 | | 0.003 | 3E-27 | 0.092 | 0.115 | 0.4243 | 0.066 | 0.036 | 0.064 | 0.057 | 0.039 | 0.142 | 0.126 | 0.082 | 0.123 |
| rs3828760 | 6 | 82456984 | *FAM46A* | C | T | 0.12 | 0.12 | 0.69 | 0.03 | | 0.005 | 1E-10 | 0.279 | 0.191 | 0.144 | 0.027 | 0.059 | 0.646 | 0.012 | 0.065 | 0.854 | 0.140 | 0.133 | 0.293 |
| rs761391 | 6 | 85448103 | *TBX18* | C | T | 0.54 | 0.54 | 0.82 | -0.019 | | 0.003 | 8E-09 | -0.126 | 0.116 | 0.2768 | -0.052 | 0.035 | 0.130 | -0.056 | 0.038 | 0.141 | -0.026 | 0.081 | 0.750 |
| rs314263 | 6 | 105392745 | *LIN28B* | C | T | 0.68 | 0.69 | 1.00 | -0.043 | | 0.003 | 1E-42 | -0.468 | 0.113 | <.0001 | -0.008 | 0.034 | 0.811 | -0.013 | 0.037 | 0.716 | 0.033 | 0.076 | 0.664 |
| rs479744 | 6 | 109020032 | *FOXO3* | G | T | 0.21 | 0.23 | 0.79 | -0.025 | | 0.004 | 8E-12 | -0.165 | 0.141 | 0.2426 | -0.032 | 0.044 | 0.468 | -0.048 | 0.048 | 0.321 | 0.068 | 0.096 | 0.477 |
| rs6920372 | 6 | 109723939 | *PPIL6* | G | A | 0.41 | 0.43 | 0.99 | -0.025 | | 0.003 | 2E-17 | -0.393 | 0.105 | 0.0002 | -0.054 | 0.032 | 0.099 | -0.050 | 0.036 | 0.156 | -0.075 | 0.072 | 0.296 |
| rs2145357 | 6 | 116451442 | *NT5DC1* | A | G | 0.27 | 0.27 | 0.96 | 0.021 | | 0.003 | 4E-10 | 0.242 | 0.119 | 0.0423 | -0.004 | 0.037 | 0.923 | -0.003 | 0.041 | 0.948 | -0.017 | 0.078 | 0.831 |
| rs1405212 | 6 | 117490664 | *VGLL2* | T | C | 0.59 | 0.65 | 0.88 | 0.023 | | 0.003 | 2E-14 | 0.092 | 0.115 | 0.423 | -0.007 | 0.035 | 0.852 | 0.004 | 0.039 | 0.914 | -0.076 | 0.077 | 0.324 |
| rs389663 | 6 | 117868051 | *DCBLD1* | T | C | 0.68 | 0.67 | 0.75 | -0.022 | | 0.003 | 3E-12 | -0.313 | 0.129 | 0.0153 | 0.046 | 0.039 | 0.241 | 0.066 | 0.044 | 0.128 | -0.090 | 0.083 | 0.279 |
| rs4895801 | 6 | 126216403 | *NCOA7* | C | G | 0.47 | 0.44 | 0.89 | 0.017 | | 0.003 | 6E-09 | 0.224 | 0.112 | 0.0461 | 0.033 | 0.034 | 0.332 | 0.011 | 0.038 | 0.782 | 0.182 | 0.075 | 0.016 |
| rs1155939 | 6 | 126866133 | *C6orf173* | C | A | 0.50 | 0.51 | 0.99 | 0.042 | | 0.003 | 1E-45 | 0.166 | 0.105 | 0.1132 | 0.064 | 0.033 | 0.049 | 0.066 | 0.036 | 0.067 | 0.054 | 0.070 | 0.442 |
| rs1415701 | 6 | 130345835 | *L3MBTL3* | G | A | 0.27 | 0.26 | 1.00 | -0.044 | | 0.004 | 2E-34 | -0.219 | 0.120 | 0.0685 | -0.032 | 0.036 | 0.376 | -0.052 | 0.039 | 0.180 | 0.116 | 0.082 | 0.157 |
| rs7740107 | 6 | 130374461 | *L3MBTL3* | T | A | 0.74 | 0.75 | 1.00 | -0.042 | | 0.003 | 2E-36 | -0.220 | 0.120 | 0.0676 | -0.043 | 0.036 | 0.231 | -0.062 | 0.040 | 0.117 | 0.092 | 0.083 | 0.268 |
| rs6921207 | 6 | 131327956 | *EPB41L2* | G | A | 0.37 | 0.37 | 0.95 | 0.023 | | 0.003 | 3E-14 | 0.347 | 0.111 | 0.0017 | -0.020 | 0.034 | 0.566 | -0.023 | 0.038 | 0.543 | 0.001 | 0.078 | 0.990 |
| rs7745166 | 6 | 142617680 | *GPR126* | C | A | 0.43 | 0.46 | 0.69 | -0.026 | | 0.003 | 4E-16 | -0.015 | 0.127 | 0.9092 | -0.016 | 0.038 | 0.679 | -0.018 | 0.041 | 0.665 | -0.001 | 0.088 | 0.987 |
| rs4896582 | 6 | 142703877 | *GPR126* | G | A | 0.30 | 0.32 | 0.99 | -0.051 | | 0.003 | 3E-55 | -0.293 | 0.113 | 0.0098 | -0.018 | 0.035 | 0.601 | -0.015 | 0.039 | 0.702 | -0.042 | 0.072 | 0.564 |
| rs2748483 | 6 | 146335560 | *GRM1* | A | T | 0.45 | 0.43 | 0.94 | -0.019 | | 0.003 | 4E-10 | -0.067 | 0.108 | 0.535 | -0.025 | 0.034 | 0.452 | -0.032 | 0.037 | 0.399 | 0.015 | 0.073 | 0.835 |
| rs6902771 | 6 | 152157881 | *ESR1* | C | T | 0.46 | 0.46 | 0.98 | 0.031 | | 0.003 | 7E-25 | 0.259 | 0.107 | 0.0151 | 0.091 | 0.032 | 0.005 | 0.084 | 0.036 | 0.018 | 0.140 | 0.072 | 0.051 |
| rs3020418 | 6 | 152345162 | *ESR1* | G | A | 0.30 | 0.28 | 1.00 | 0.032 | | 0.003 | 8E-24 | 0.081 | 0.116 | 0.4846 | 0.004 | 0.036 | 0.904 | 0.022 | 0.039 | 0.573 | -0.129 | 0.084 | 0.124 |
| rs11156098 | 6 | 156587831 | *ARID1B* | T | C | 0.88 | 0.90 | 0.90 | -0.027 | | 0.005 | 2E-08 | -0.635 | 0.180 | 0.0004 | -0.059 | 0.056 | 0.298 | -0.052 | 0.062 | 0.398 | -0.098 | 0.128 | 0.444 |
| rs1832871 | 6 | 158722034 | *TULP4* | A | G | 0.66 | 0.68 | 0.82 | -0.025 | | 0.003 | 2E-15 | -0.182 | 0.123 | 0.1391 | -0.023 | 0.037 | 0.526 | -0.037 | 0.040 | 0.360 | 0.072 | 0.082 | 0.380 |
| rs9456307 | 6 | 158929442 | *TULP4* | T | A | 0.06 | 0.05 | 0.49 | -0.053 | | 0.007 | 2E-15 | -0.747 | 0.352 | 0.0339 | 0.128 | 0.099 | 0.194 | 0.156 | 0.108 | 0.148 | -0.049 | 0.236 | 0.835 |
| rs991946 | 6 | 166329862 | *T* | C | T | 0.48 | 0.45 | 0.82 | -0.021 | | 0.003 | 8E-13 | -0.017 | 0.117 | 0.8871 | -0.032 | 0.038 | 0.401 | -0.034 | 0.042 | 0.419 | -0.008 | 0.075 | 0.914 |
| rs2763273 | 6 | 168834623 | *SMOC2* | C | T | 0.24 | 0.23 | 0.92 | -0.022 | | 0.003 | 3E-10 | -0.299 | 0.131 | 0.0221 | 0.043 | 0.040 | 0.286 | 0.046 | 0.045 | 0.309 | 0.018 | 0.084 | 0.832 |
| rs7774834 | 6 | 169349731 | *THBS2* | C | A | 0.51 | 0.50 | 0.99 | 0.018 | | 0.003 | 5E-10 | 0.001 | 0.104 | 0.9945 | -0.029 | 0.032 | 0.366 | -0.024 | 0.035 | 0.492 | -0.062 | 0.071 | 0.385 |
| rs798497 | 7 | 2795957 | *GNA12* | A | G | 0.30 | 0.29 | 0.97 | -0.057 | | 0.003 | 2E-71 | -0.291 | 0.117 | 0.0131 | 0.032 | 0.036 | 0.367 | 0.042 | 0.039 | 0.280 | -0.040 | 0.080 | 0.613 |
| rs4725061 | 7 | 8086639 | *GLCCI1* | A | G | 0.44 | 0.43 | 0.71 | 0.02 | | 0.003 | 1E-10 | 0.163 | 0.126 | 0.1942 | -0.031 | 0.038 | 0.420 | -0.011 | 0.042 | 0.787 | -0.169 | 0.088 | 0.055 |
| rs929637 | 7 | 12276522 | *TMEM106B* | G | T | 0.22 | 0.23 | 1.00 | -0.021 | | 0.004 | 2E-09 | -0.020 | 0.126 | 0.8755 | -0.012 | 0.038 | 0.761 | -0.002 | 0.042 | 0.970 | -0.083 | 0.086 | 0.336 |
| rs17140875 | 7 | 19248278 | *FERD3L* | G | T | 0.05 | 0.05 | 0.58 | -0.043 | | 0.007 | 7E-09 | -0.008 | 0.300 | 0.9788 | 0.111 | 0.092 | 0.228 | 0.118 | 0.101 | 0.245 | 0.072 | 0.205 | 0.724 |
| rs2390151 | 7 | 19642100 | *TWISTNB* | G | T | 0.18 | 0.19 | 0.92 | 0.033 | | 0.004 | 3E-17 | 0.155 | 0.140 | 0.2665 | -0.043 | 0.045 | 0.338 | -0.058 | 0.050 | 0.243 | 0.051 | 0.095 | 0.587 |
| rs3807931 | 7 | 20381674 | *ITGB8* | G | A | 0.45 | 0.46 | 0.95 | 0.027 | | 0.003 | 1E-19 | -0.045 | 0.108 | 0.6769 | -0.026 | 0.033 | 0.439 | -0.019 | 0.037 | 0.607 | -0.071 | 0.072 | 0.324 |
| rs12538407 | 7 | 23521316 | *IGF2BP3* | A | G | 0.40 | 0.39 | 0.99 | -0.032 | | 0.003 | 4E-26 | -0.122 | 0.107 | 0.2564 | -0.019 | 0.032 | 0.551 | -0.019 | 0.035 | 0.582 | -0.019 | 0.073 | 0.797 |
| rs1055144 | 7 | 25871109 | *NFE2L3* | C | T | 0.19 | 0.18 | 1.00 | 0.021 | | 0.004 | 8E-09 | 0.175 | 0.135 | 0.193 | 0.019 | 0.041 | 0.640 | 0.031 | 0.045 | 0.493 | -0.060 | 0.099 | 0.544 |
| rs552707 | 7 | 28205303 | *JAZF1* | T | C | 0.69 | 0.72 | 0.99 | -0.046 | | 0.003 | 9E-46 | -0.196 | 0.117 | 0.0948 | -0.035 | 0.036 | 0.336 | -0.054 | 0.039 | 0.171 | 0.104 | 0.081 | 0.200 |
| rs12533079 | 7 | 28751137 | *CREB5* | T | G | 0.21 | 0.21 | 1.00 | -0.022 | | 0.004 | 2E-09 | -0.272 | 0.129 | 0.0345 | -0.022 | 0.040 | 0.575 | -0.028 | 0.044 | 0.527 | 0.014 | 0.087 | 0.875 |
| rs6462432 | 7 | 32935524 | *KBTBD2* | G | A | 0.39 | 0.40 | 0.65 | 0.017 | | 0.003 | 3E-08 | 0.197 | 0.128 | 0.1248 | 0.035 | 0.040 | 0.376 | 0.052 | 0.044 | 0.233 | -0.084 | 0.091 | 0.357 |
| rs6974574 | 7 | 38110073 | *STARD3NL* | A | T | 0.69 | 0.66 | 0.93 | 0.03 | | 0.003 | 1E-18 | 0.281 | 0.114 | 0.0134 | -0.046 | 0.034 | 0.184 | -0.048 | 0.038 | 0.203 | -0.023 | 0.076 | 0.765 |
| rs1007358 | 7 | 46201355 | *IGFBP3* | A | G | 0.23 | 0.23 | 0.60 | 0.021 | | 0.004 | 9E-10 | 0.114 | 0.162 | 0.4817 | 0.018 | 0.049 | 0.713 | 0.035 | 0.054 | 0.516 | -0.103 | 0.106 | 0.329 |
| rs6949739 | 7 | 46417403 | *IGFBP3* | T | A | 0.09 | 0.08 | 0.97 | -0.038 | | 0.005 | 7E-13 | -0.338 | 0.196 | 0.0858 | 0.077 | 0.062 | 0.214 | 0.073 | 0.069 | 0.290 | 0.096 | 0.129 | 0.458 |
| rs2715094 | 7 | 50730452 | *GRB10* | G | A | 0.75 | 0.79 | 0.79 | -0.021 | | 0.003 | 9E-10 | -0.070 | 0.144 | 0.6253 | -0.006 | 0.043 | 0.894 | -0.009 | 0.047 | 0.842 | 0.013 | 0.105 | 0.904 |
| rs1113765 | 7 | 55889334 | *14-Sep* | G | A | 0.19 | 0.21 | 0.71 | -0.024 | | 0.004 | 2E-10 | -0.437 | 0.153 | 0.0041 | -0.014 | 0.046 | 0.761 | -0.043 | 0.051 | 0.397 | 0.171 | 0.097 | 0.076 |
| rs12669267 | 7 | 73304636 | *WBSCR28* | C | T | 0.13 | 0.14 | 0.52 | -0.029 | | 0.005 | 3E-08 | -0.401 | 0.200 | 0.0453 | -0.022 | 0.062 | 0.720 | -0.024 | 0.068 | 0.721 | -0.011 | 0.142 | 0.938 |
| rs17807185 | 7 | 77308295 | *RSBN1L* | A | G | 0.38 | 0.39 | 0.62 | 0.022 | | 0.003 | 4E-13 | 0.266 | 0.136 | 0.0505 | 0.022 | 0.041 | 0.589 | 0.037 | 0.045 | 0.404 | -0.087 | 0.095 | 0.364 |
| rs2888877 | 7 | 92228400 | *CDK6* | T | C | 0.78 | 0.79 | 0.96 | -0.066 | | 0.004 | 4E-69 | -0.361 | 0.133 | 0.0065 | 0.024 | 0.041 | 0.552 | 0.007 | 0.044 | 0.871 | 0.154 | 0.096 | 0.108 |
| rs42039 | 7 | 92244422 | *CDK6* | C | T | 0.27 | 0.26 | 1.00 | 0.068 | | 0.003 | 4E-88 | 0.241 | 0.121 | 0.046 | -0.003 | 0.037 | 0.928 | 0.018 | 0.041 | 0.665 | -0.151 | 0.079 | 0.054 |
| rs6971575 | 7 | 96039648 | *SLC25A13* | C | G | 0.71 | 0.73 | 0.80 | -0.021 | | 0.004 | 3E-09 | -0.132 | 0.128 | 0.3036 | -0.043 | 0.040 | 0.284 | -0.069 | 0.044 | 0.114 | 0.142 | 0.088 | 0.108 |
| rs17250196 | 7 | 99817196 | *GATS/PVR* | G | T | 0.06 | 0.05 | 0.88 | 0.045 | | 0.007 | 4E-10 | 0.886 | 0.259 | 0.0006 | -0.088 | 0.084 | 0.296 | -0.102 | 0.093 | 0.273 | 0.032 | 0.170 | 0.850 |
| rs6952113 | 7 | 120777619 | *C7orf58* | G | A | 0.38 | 0.40 | 0.93 | -0.018 | | 0.003 | 1E-09 | -0.195 | 0.111 | 0.0805 | 0.028 | 0.034 | 0.396 | 0.025 | 0.037 | 0.497 | 0.049 | 0.073 | 0.502 |
| rs6962887 | 7 | 135045786 | *CNOT4* | T | G | 0.32 | 0.31 | 0.61 | -0.023 | | 0.003 | 6E-11 | -0.375 | 0.144 | 0.0091 | 0.019 | 0.045 | 0.681 | 0.014 | 0.050 | 0.776 | 0.043 | 0.096 | 0.653 |
| rs273945 | 7 | 137611566 | *CREB3L2* | A | C | 0.58 | 0.58 | 1.00 | 0.019 | | 0.003 | 1E-09 | 0.284 | 0.105 | 0.007 | 0.058 | 0.032 | 0.070 | 0.081 | 0.035 | 0.021 | -0.096 | 0.070 | 0.167 |
| rs822531 | 7 | 148629759 | *EZH2* | C | T | 0.78 | 0.77 | 0.67 | 0.036 | | 0.004 | 2E-18 | -0.027 | 0.155 | 0.8606 | -0.015 | 0.047 | 0.754 | -0.034 | 0.051 | 0.513 | 0.127 | 0.113 | 0.262 |
| rs6955948 | 7 | 150508720 | *TMEM176A* | C | T | 0.28 | 0.29 | 0.99 | 0.031 | | 0.003 | 5E-20 | 0.015 | 0.114 | 0.8962 | 0.011 | 0.035 | 0.753 | -0.007 | 0.039 | 0.849 | 0.152 | 0.079 | 0.054 |
| rs4875421 | 8 | 4827332 | *CSMD1* | T | A | 0.55 | 0.53 | 0.95 | -0.019 | | 0.003 | 1E-10 | 0.090 | 0.108 | 0.4047 | 0.012 | 0.034 | 0.715 | -0.004 | 0.037 | 0.920 | 0.122 | 0.070 | 0.083 |
| rs429433 | 8 | 8747894 | *MFHAS1* | A | G | 0.95 | 0.96 | 0.60 | -0.046 | | 0.007 | 1E-10 | 0.128 | 0.343 | 0.7091 | -0.009 | 0.102 | 0.928 | -0.025 | 0.112 | 0.821 | 0.103 | 0.237 | 0.664 |
| rs7834383 | 8 | 13273477 | *DLC1* | G | T | 0.35 | 0.32 | 0.87 | 0.022 | | 0.003 | 2E-11 | 0.269 | 0.118 | 0.0228 | 0.019 | 0.037 | 0.605 | 0.021 | 0.041 | 0.604 | 0.001 | 0.079 | 0.987 |
| rs7823327 | 8 | 22562352 | *PEBP4* | G | T | 0.49 | 0.50 | 0.67 | 0.019 | | 0.003 | 6E-11 | 0.193 | 0.126 | 0.1253 | 0.008 | 0.040 | 0.840 | 0.016 | 0.044 | 0.727 | -0.046 | 0.085 | 0.593 |
| rs4273857 | 8 | 23173053 | *LOXL2* | A | G | 0.76 | 0.77 | 0.99 | -0.027 | | 0.003 | 9E-15 | -0.175 | 0.123 | 0.1546 | 0.009 | 0.039 | 0.816 | 0.029 | 0.043 | 0.490 | -0.134 | 0.084 | 0.112 |
| rs17088184 | 8 | 23375235 | *SLC25A37* | C | G | 0.17 | 0.15 | 1.00 | 0.025 | | 0.004 | 5E-10 | 0.111 | 0.146 | 0.4455 | -0.047 | 0.046 | 0.303 | -0.035 | 0.050 | 0.488 | -0.135 | 0.102 | 0.184 |
| rs2013265 | 8 | 24092500 | *ADAM28* | C | T | 0.25 | 0.22 | 0.62 | -0.028 | | 0.003 | 2E-16 | -0.468 | 0.160 | 0.0034 | 0.104 | 0.047 | 0.029 | 0.119 | 0.052 | 0.022 | 0.005 | 0.106 | 0.965 |
| rs3812423 | 8 | 25298710 | *KCTD9* | G | C | 0.36 | 0.36 | 0.99 | -0.021 | | 0.003 | 2E-12 | -0.209 | 0.110 | 0.0568 | -0.035 | 0.034 | 0.305 | -0.038 | 0.037 | 0.306 | -0.025 | 0.076 | 0.747 |
| rs568610 | 8 | 27527995 | *SCARA3* | C | T | 0.24 | 0.26 | 0.83 | 0.022 | | 0.003 | 1E-10 | -0.210 | 0.130 | 0.1074 | -0.039 | 0.039 | 0.319 | -0.032 | 0.043 | 0.450 | -0.090 | 0.091 | 0.320 |
| rs6988484 | 8 | 49413780 | *EFCAB1* | T | C | 0.25 | 0.27 | 0.96 | 0.022 | | 0.003 | 5E-11 | 0.027 | 0.123 | 0.8257 | -0.025 | 0.036 | 0.493 | -0.047 | 0.040 | 0.239 | 0.121 | 0.080 | 0.134 |
| rs10958476 | 8 | 57095808 | *PLAG1* | T | C | 0.21 | 0.20 | 1.00 | 0.051 | | 0.004 | 2E-40 | 0.254 | 0.130 | 0.0512 | 0.027 | 0.040 | 0.503 | 0.018 | 0.044 | 0.673 | 0.088 | 0.088 | 0.319 |
| rs9650315 | 8 | 57155598 | *CHCHD7* | G | T | 0.13 | 0.16 | 0.81 | -0.061 | | 0.005 | 2E-41 | -0.467 | 0.163 | 0.0042 | 0.042 | 0.049 | 0.392 | 0.060 | 0.054 | 0.268 | -0.084 | 0.103 | 0.414 |
| rs2956605 | 8 | 75883054 | *CRISPLD1* | A | C | 0.62 | 0.61 | 1.00 | -0.024 | | 0.003 | 5E-15 | -0.191 | 0.108 | 0.0773 | -0.024 | 0.033 | 0.464 | -0.033 | 0.036 | 0.364 | 0.041 | 0.072 | 0.567 |
| rs4735677 | 8 | 78148191 | *PXMP3* | A | T | 0.28 | 0.29 | 1.00 | 0.037 | | 0.003 | 6E-30 | 0.234 | 0.116 | 0.0442 | 0.010 | 0.036 | 0.777 | 0.033 | 0.039 | 0.397 | -0.156 | 0.080 | 0.052 |
| rs2737220 | 8 | 116637685 | *TRPS1* | T | C | 0.62 | 0.62 | 1.00 | -0.017 | | 0.003 | 1E-08 | -0.233 | 0.107 | 0.029 | -0.046 | 0.032 | 0.150 | -0.043 | 0.035 | 0.226 | -0.071 | 0.072 | 0.324 |
| rs1550162 | 8 | 117563532 | *EIF3H* | G | A | 0.71 | 0.73 | 1.00 | -0.024 | | 0.003 | 1E-13 | -0.177 | 0.118 | 0.1313 | 0.047 | 0.036 | 0.190 | 0.030 | 0.040 | 0.451 | 0.165 | 0.079 | 0.037 |
| rs1599473 | 8 | 120475358 | *NOV* | G | T | 0.25 | 0.24 | 0.91 | -0.027 | | 0.003 | 1E-14 | -0.114 | 0.130 | 0.3782 | 0.041 | 0.041 | 0.310 | 0.047 | 0.045 | 0.289 | 0.007 | 0.089 | 0.941 |
| rs8180991 | 8 | 126500350 | *TRIB1* | C | G | 0.23 | 0.22 | 0.79 | -0.028 | | 0.004 | 3E-15 | -0.229 | 0.143 | 0.1101 | 0.043 | 0.044 | 0.329 | 0.065 | 0.048 | 0.174 | -0.122 | 0.103 | 0.239 |
| rs4733724 | 8 | 130723728 | *MLZE* | A | G | 0.20 | 0.21 | 0.95 | -0.05 | | 0.004 | 1E-41 | -0.192 | 0.132 | 0.1445 | -0.067 | 0.040 | 0.095 | -0.088 | 0.045 | 0.051 | 0.063 | 0.084 | 0.454 |
| rs1036821 | 8 | 135650483 | *ZFAT* | G | A | 0.30 | 0.30 | 1.00 | -0.037 | | 0.003 | 1E-30 | -0.321 | 0.114 | 0.0049 | 0.011 | 0.035 | 0.745 | -0.009 | 0.039 | 0.821 | 0.150 | 0.081 | 0.062 |
| rs11783655 | 8 | 145037573 | *PLEC1* | T | A | 0.40 | 0.40 | 0.95 | -0.018 | | 0.003 | 1E-09 | -0.090 | 0.110 | 0.4124 | 0.012 | 0.034 | 0.719 | 0.031 | 0.037 | 0.395 | -0.129 | 0.075 | 0.087 |
| rs7033940 | 9 | 6440419 | *UHRF2* | G | C | 0.13 | 0.12 | 0.98 | -0.024 | | 0.004 | 5E-08 | 0.099 | 0.163 | 0.5441 | -0.036 | 0.051 | 0.475 | -0.037 | 0.056 | 0.513 | -0.034 | 0.105 | 0.743 |
| rs2149163 | 9 | 16455833 | *BNC2* | G | C | 0.40 | 0.38 | 0.95 | 0.02 | | 0.003 | 2E-11 | 0.331 | 0.111 | 0.0028 | -0.014 | 0.035 | 0.689 | -0.012 | 0.039 | 0.762 | -0.029 | 0.076 | 0.703 |
| rs3927536 | 9 | 16787670 | *BNC2* | C | T | 0.78 | 0.75 | 0.90 | 0.021 | | 0.004 | 2E-09 | 0.151 | 0.128 | 0.2403 | -0.044 | 0.038 | 0.243 | -0.059 | 0.042 | 0.159 | 0.053 | 0.086 | 0.540 |
| rs10962832 | 9 | 17048990 | *CNTLN* | A | T | 0.16 | 0.15 | 0.99 | -0.024 | | 0.004 | 4E-08 | 0.043 | 0.144 | 0.7644 | -0.006 | 0.044 | 0.886 | 0.005 | 0.049 | 0.915 | -0.087 | 0.099 | 0.379 |
| rs1576900 | 9 | 18629792 | *ADAMTSL1* | G | A | 0.30 | 0.30 | 0.91 | -0.019 | | 0.003 | 7E-09 | -0.243 | 0.119 | 0.0414 | 0.003 | 0.037 | 0.924 | 0.018 | 0.040 | 0.652 | -0.101 | 0.086 | 0.240 |
| rs3763631 | 9 | 35808334 | *NPR2/SPA* | C | G | 0.31 | 0.30 | 1.00 | -0.019 | | 0.003 | 1E-09 | -0.167 | 0.114 | 0.1429 | 0.002 | 0.035 | 0.952 | -0.019 | 0.039 | 0.626 | 0.144 | 0.076 | 0.057 |
| rs958225 | 9 | 78759705 | *PCSK5* | T | A | 0.05 | 0.07 | 0.50 | 0.046 | | 0.008 | 7E-09 | -0.058 | 0.288 | 0.8402 | -0.085 | 0.088 | 0.337 | -0.071 | 0.096 | 0.462 | -0.180 | 0.200 | 0.367 |
| rs7853235 | 9 | 86660782 | *RMI1* | T | C | 0.80 | 0.80 | 0.94 | -0.029 | | 0.004 | 8E-15 | -0.409 | 0.134 | 0.0023 | -0.015 | 0.042 | 0.710 | -0.010 | 0.046 | 0.835 | -0.058 | 0.092 | 0.533 |
| rs181338 | 9 | 89108161 | *ZCCHC6* | C | T | 0.51 | 0.51 | 0.96 | 0.028 | | 0.003 | 3E-22 | 0.194 | 0.106 | 0.0675 | 0.001 | 0.032 | 0.971 | 0.008 | 0.035 | 0.828 | -0.044 | 0.071 | 0.533 |
| rs7043114 | 9 | 95387983 | *IPPK* | C | T | 0.56 | 0.60 | 0.70 | -0.029 | | 0.003 | 2E-22 | -0.130 | 0.127 | 0.3034 | 0.005 | 0.040 | 0.890 | 0.018 | 0.044 | 0.673 | -0.087 | 0.090 | 0.335 |
| rs1257763 | 9 | 96893945 | *PTPDC1* | A | G | 0.96 | 0.96 | 0.72 | -0.078 | | 0.01 | 2E-16 | 0.135 | 0.325 | 0.6792 | -0.046 | 0.099 | 0.646 | 0.000 | 0.113 | 0.998 | -0.296 | 0.180 | 0.101 |
| rs12347744 | 9 | 97575273 | *C9orf3* | C | T | 0.07 | 0.06 | 0.77 | -0.039 | | 0.006 | 5E-11 | -0.725 | 0.251 | 0.0039 | 0.101 | 0.079 | 0.204 | 0.146 | 0.087 | 0.093 | -0.207 | 0.184 | 0.262 |
| rs4448343 | 9 | 98266370 | *PTCH1* | A | G | 0.35 | 0.34 | 1.00 | 0.035 | | 0.003 | 5E-30 | 0.166 | 0.111 | 0.1337 | -0.013 | 0.034 | 0.693 | -0.008 | 0.037 | 0.823 | -0.050 | 0.079 | 0.523 |
| rs1329393 | 9 | 98318926 | *PTCH1* | C | T | 0.16 | 0.17 | 0.71 | 0.034 | | 0.005 | 2E-13 | -0.127 | 0.164 | 0.438 | 0.039 | 0.050 | 0.441 | 0.025 | 0.055 | 0.654 | 0.135 | 0.112 | 0.230 |
| rs817300 | 9 | 98380222 | *PTCH1* | G | A | 0.07 | 0.06 | 0.62 | -0.085 | | 0.007 | 4E-34 | -0.911 | 0.269 | 0.0007 | -0.050 | 0.088 | 0.567 | -0.063 | 0.097 | 0.519 | 0.040 | 0.178 | 0.822 |
| rs10990303 | 9 | 98410405 | *PTCH1* | C | T | 0.23 | 0.22 | 0.91 | 0.036 | | 0.004 | 4E-24 | 0.163 | 0.130 | 0.2102 | 0.019 | 0.041 | 0.640 | 0.025 | 0.045 | 0.584 | -0.020 | 0.088 | 0.819 |
| rs7870753 | 9 | 99201585 | *HABP4* | A | G | 0.22 | 0.23 | 0.98 | 0.043 | | 0.004 | 4E-33 | 0.504 | 0.128 | <.0001 | -0.066 | 0.040 | 0.093 | -0.075 | 0.044 | 0.085 | -0.007 | 0.088 | 0.941 |
| rs989393 | 9 | 101743336 | *COL15A1* | T | C | 0.29 | 0.29 | 0.76 | -0.022 | | 0.003 | 3E-11 | -0.066 | 0.132 | 0.6185 | 0.003 | 0.040 | 0.948 | 0.008 | 0.045 | 0.853 | -0.039 | 0.083 | 0.643 |
| rs9409082 | 9 | 108901049 | *TMEM38B* | C | T | 0.24 | 0.25 | 0.97 | -0.028 | | 0.004 | 9E-15 | 0.093 | 0.123 | 0.4489 | 0.048 | 0.036 | 0.186 | 0.057 | 0.040 | 0.154 | -0.011 | 0.082 | 0.888 |
| rs902143 | 9 | 109181911 | *ZNF462* | C | T | 0.45 | 0.42 | 0.94 | 0.02 | | 0.003 | 1E-11 | 0.052 | 0.109 | 0.6322 | -0.059 | 0.033 | 0.075 | -0.068 | 0.037 | 0.063 | 0.002 | 0.072 | 0.975 |
| rs7027110 | 9 | 109599046 | *ZNF462* | G | A | 0.23 | 0.22 | 0.88 | 0.032 | | 0.003 | 2E-20 | 0.045 | 0.133 | 0.7369 | -0.017 | 0.042 | 0.688 | -0.002 | 0.047 | 0.957 | -0.113 | 0.091 | 0.216 |
| rs3739707 | 9 | 113792706 | *LPAR1* | C | A | 0.25 | 0.29 | 0.69 | -0.024 | | 0.004 | 4E-12 | -0.158 | 0.140 | 0.2587 | 0.016 | 0.042 | 0.700 | 0.045 | 0.046 | 0.327 | -0.202 | 0.101 | 0.044 |
| rs10119624 | 9 | 118305438 | *1-Dec* | G | A | 0.67 | 0.66 | 0.97 | 0.024 | | 0.003 | 4E-14 | 0.163 | 0.111 | 0.1421 | 0.017 | 0.033 | 0.602 | 0.029 | 0.037 | 0.433 | -0.053 | 0.074 | 0.473 |
| rs12344396 | 9 | 118921327 | *PAPPA* | G | C | 0.62 | 0.56 | 0.71 | 0.019 | | 0.003 | 8E-10 | -0.016 | 0.128 | 0.9031 | 0.022 | 0.038 | 0.568 | 0.011 | 0.042 | 0.797 | 0.099 | 0.087 | 0.257 |
| rs7033487 | 9 | 119129257 | *PAPPA* | T | C | 0.21 | 0.19 | 1.00 | -0.037 | | 0.004 | 1E-24 | -0.288 | 0.131 | 0.028 | 0.077 | 0.041 | 0.061 | 0.083 | 0.045 | 0.068 | 0.040 | 0.090 | 0.657 |
| rs1742829 | 9 | 119422807 | *ASTN2* | T | A | 0.92 | 0.92 | 0.99 | -0.037 | | 0.006 | 2E-11 | -0.190 | 0.200 | 0.3411 | -0.018 | 0.060 | 0.759 | -0.018 | 0.066 | 0.781 | -0.020 | 0.129 | 0.876 |
| rs7466269 | 9 | 133464084 | *FUBP3* | A | G | 0.36 | 0.38 | 0.66 | -0.033 | | 0.003 | 1E-27 | -0.126 | 0.132 | 0.3401 | -0.024 | 0.042 | 0.568 | 0.006 | 0.046 | 0.892 | -0.224 | 0.093 | 0.016 |
| rs3132297 | 9 | 137301866 | *RXRA* | A | G | 0.83 | 0.82 | 0.90 | 0.023 | | 0.004 | 4E-08 | 0.092 | 0.146 | 0.5276 | -0.008 | 0.044 | 0.859 | 0.003 | 0.048 | 0.954 | -0.081 | 0.098 | 0.407 |
| rs7849585 | 9 | 139111870 | *QSOX2* | G | T | 0.33 | 0.32 | 0.93 | 0.036 | | 0.003 | 1E-29 | 0.112 | 0.116 | 0.3348 | 0.023 | 0.036 | 0.521 | 0.023 | 0.039 | 0.556 | 0.019 | 0.080 | 0.810 |
| rs3812591 | 9 | 139341612 | *SEC16A* | T | C | 0.27 | 0.27 | 0.99 | 0.024 | | 0.003 | 7E-13 | 0.198 | 0.119 | 0.0972 | -0.052 | 0.037 | 0.162 | -0.043 | 0.040 | 0.286 | -0.113 | 0.085 | 0.182 |
| rs4332428 | 10 | 4965434 | *AKR1C1* | A | G | 0.12 | 0.13 | 0.72 | -0.036 | | 0.005 | 2E-15 | -0.187 | 0.182 | 0.3037 | -0.016 | 0.055 | 0.774 | -0.033 | 0.060 | 0.577 | 0.105 | 0.122 | 0.390 |
| rs12779328 | 10 | 12943973 | *CCDC3* | C | T | 0.28 | 0.30 | 0.94 | -0.028 | | 0.003 | 2E-17 | -0.184 | 0.118 | 0.1175 | -0.080 | 0.036 | 0.026 | -0.089 | 0.040 | 0.026 | -0.020 | 0.076 | 0.789 |
| rs4350272 | 10 | 25056118 | *ARHGAP21* | A | G | 0.72 | 0.74 | 0.85 | -0.02 | | 0.003 | 3E-09 | -0.152 | 0.128 | 0.2337 | 0.076 | 0.039 | 0.052 | 0.063 | 0.042 | 0.140 | 0.172 | 0.092 | 0.063 |
| rs7069985 | 10 | 27890831 | *RAB18* | A | G | 0.25 | 0.22 | 0.94 | 0.023 | | 0.003 | 2E-11 | -0.010 | 0.130 | 0.9395 | 0.061 | 0.039 | 0.117 | 0.070 | 0.043 | 0.105 | 0.007 | 0.085 | 0.938 |
| rs10995319 | 10 | 52762887 | *PRKG1* | T | C | 0.24 | 0.23 | 0.96 | -0.019 | | 0.003 | 3E-08 | -0.128 | 0.126 | 0.3091 | -0.038 | 0.039 | 0.329 | -0.053 | 0.043 | 0.221 | 0.063 | 0.080 | 0.430 |
| rs1171615 | 10 | 61469090 | *SLC16A9* | C | T | 0.78 | 0.81 | 0.67 | -0.022 | | 0.004 | 6E-09 | -0.208 | 0.163 | 0.1996 | 0.022 | 0.049 | 0.655 | 0.013 | 0.054 | 0.805 | 0.080 | 0.113 | 0.480 |
| rs10997979 | 10 | 69937192 | *MYPN* | A | G | 0.50 | 0.47 | 0.99 | 0.021 | | 0.003 | 4E-13 | 0.317 | 0.104 | 0.0024 | -0.027 | 0.033 | 0.418 | -0.022 | 0.037 | 0.557 | -0.053 | 0.070 | 0.454 |
| rs4746769 | 10 | 70196580 | *DNA2* | T | C | 0.14 | 0.16 | 0.80 | -0.029 | | 0.004 | 9E-12 | 0.019 | 0.162 | 0.9066 | 0.034 | 0.047 | 0.476 | 0.006 | 0.052 | 0.905 | 0.205 | 0.098 | 0.037 |
| rs1815314 | 10 | 80928793 | *ZMIZ1* | G | A | 0.42 | 0.41 | 1.00 | -0.022 | | 0.003 | 5E-14 | -0.122 | 0.105 | 0.242 | -0.011 | 0.032 | 0.724 | -0.010 | 0.035 | 0.780 | -0.023 | 0.071 | 0.747 |
| rs1923367 | 10 | 81132829 | *ZCCHC24* | G | C | 0.48 | 0.50 | 0.92 | -0.03 | | 0.003 | 5E-24 | -0.031 | 0.110 | 0.7747 | 0.007 | 0.034 | 0.826 | -0.016 | 0.037 | 0.663 | 0.176 | 0.076 | 0.021 |
| rs2631676 | 10 | 93037409 | *PCGF5* | A | G | 0.19 | 0.17 | 0.99 | 0.028 | | 0.004 | 5E-13 | 0.227 | 0.137 | 0.0995 | 0.033 | 0.043 | 0.445 | 0.043 | 0.047 | 0.365 | -0.034 | 0.095 | 0.723 |
| rs915506 | 10 | 97805074 | *CCNJ* | G | A | 0.35 | 0.35 | 0.61 | -0.021 | | 0.003 | 1E-11 | -0.006 | 0.139 | 0.9663 | -0.060 | 0.043 | 0.163 | -0.058 | 0.047 | 0.218 | -0.070 | 0.095 | 0.458 |
| rs11599750 | 10 | 101805442 | *CPN1* | C | T | 0.38 | 0.39 | 1.00 | -0.023 | | 0.003 | 3E-14 | -0.188 | 0.106 | 0.0762 | 0.001 | 0.032 | 0.978 | 0.018 | 0.035 | 0.601 | -0.123 | 0.075 | 0.102 |
| rs10883563 | 10 | 102684380 | *FAM178A* | C | A | 0.55 | 0.55 | 0.88 | 0.023 | | 0.003 | 6E-15 | 0.421 | 0.112 | 0.0002 | 0.001 | 0.034 | 0.984 | -0.002 | 0.038 | 0.955 | 0.023 | 0.076 | 0.765 |
| rs7899004 | 10 | 104341435 | *SUFU* | T | C | 0.44 | 0.42 | 0.95 | -0.025 | | 0.003 | 7E-17 | -0.065 | 0.109 | 0.549 | -0.029 | 0.034 | 0.387 | -0.052 | 0.038 | 0.165 | 0.124 | 0.074 | 0.092 |
| rs6584575 | 10 | 105577409 | *SH3PXD2A* | G | A | 0.10 | 0.11 | 0.98 | 0.034 | | 0.005 | 1E-10 | 0.145 | 0.178 | 0.4166 | -0.019 | 0.051 | 0.705 | -0.026 | 0.056 | 0.645 | 0.019 | 0.120 | 0.872 |
| rs291979 | 10 | 121129797 | *GRK5* | G | A | 0.23 | 0.23 | 0.92 | 0.029 | | 0.004 | 2E-16 | 0.325 | 0.130 | 0.0122 | -0.038 | 0.040 | 0.340 | -0.036 | 0.044 | 0.412 | -0.058 | 0.090 | 0.519 |
| rs1614303 | 10 | 123396806 | *FGFR2* | G | T | 0.83 | 0.82 | 1.00 | 0.022 | | 0.004 | 6E-09 | 0.107 | 0.136 | 0.4322 | 0.046 | 0.042 | 0.273 | 0.048 | 0.046 | 0.296 | 0.033 | 0.093 | 0.719 |
| rs10794175 | 10 | 126358073 | *FAM53B* | G | T | 0.43 | 0.44 | 1.00 | 0.02 | | 0.003 | 6E-12 | -0.011 | 0.106 | 0.9166 | 0.045 | 0.032 | 0.159 | 0.031 | 0.035 | 0.385 | 0.148 | 0.075 | 0.047 |
| rs11245515 | 10 | 126824068 | *CTBP2* | T | C | 0.44 | 0.44 | 1.00 | -0.017 | | 0.003 | 9E-09 | -0.005 | 0.105 | 0.9641 | 0.012 | 0.032 | 0.696 | 0.012 | 0.035 | 0.725 | 0.012 | 0.072 | 0.870 |
| rs11244750 | 10 | 127673877 | *FANK1* | C | T | 0.32 | 0.35 | 0.83 | 0.018 | | 0.003 | 2E-08 | 0.188 | 0.121 | 0.1199 | -0.034 | 0.038 | 0.370 | -0.038 | 0.042 | 0.357 | -0.004 | 0.082 | 0.958 |
| rs2272566 | 11 | 244552 | *PSMD13* | A | G | 0.52 | 0.55 | 0.85 | -0.016 | | 0.003 | 3E-08 | -0.049 | 0.113 | 0.6626 | 0.052 | 0.035 | 0.137 | 0.044 | 0.038 | 0.255 | 0.114 | 0.078 | 0.147 |
| rs2735469 | 11 | 2022804 | *MRPL23* | A | G | 0.84 | 0.84 | 1.00 | -0.029 | | 0.005 | 3E-10 | -0.332 | 0.144 | 0.0214 | 0.045 | 0.044 | 0.305 | 0.059 | 0.048 | 0.226 | -0.050 | 0.102 | 0.621 |
| rs4320932 | 11 | 2171601 | *INS-IGF2* | T | C | 0.19 | 0.21 | 1.00 | -0.029 | | 0.004 | 2E-12 | -0.487 | 0.129 | 0.0002 | -0.021 | 0.041 | 0.598 | -0.016 | 0.045 | 0.718 | -0.055 | 0.085 | 0.515 |
| rs17659078 | 11 | 2284590 | *ASCL2* | C | A | 0.27 | 0.25 | 1.00 | 0.021 | | 0.003 | 6E-10 | 0.074 | 0.119 | 0.5356 | 0.044 | 0.037 | 0.231 | 0.032 | 0.040 | 0.433 | 0.130 | 0.087 | 0.134 |
| rs2237886 | 11 | 2810731 | *KCNQ1* | C | T | 0.11 | 0.09 | 0.86 | 0.043 | | 0.005 | 5E-18 | 0.478 | 0.189 | 0.0116 | 0.055 | 0.059 | 0.354 | 0.066 | 0.065 | 0.305 | -0.032 | 0.135 | 0.815 |
| rs6485978 | 11 | 12678415 | *TEAD1* | T | C | 0.46 | 0.42 | 0.62 | 0.023 | | 0.003 | 1E-15 | 0.251 | 0.134 | 0.0609 | 0.050 | 0.041 | 0.221 | 0.056 | 0.045 | 0.216 | 0.010 | 0.094 | 0.919 |
| rs2099745 | 11 | 12924265 | *TEAD1* | G | A | 0.08 | 0.06 | 0.72 | -0.036 | | 0.006 | 2E-09 | 0.182 | 0.250 | 0.4677 | -0.135 | 0.080 | 0.091 | -0.166 | 0.089 | 0.061 | 0.036 | 0.174 | 0.838 |
| rs10766065 | 11 | 13277961 | *ARNTL* | C | T | 0.73 | 0.71 | 0.97 | -0.019 | | 0.003 | 3E-08 | 0.027 | 0.116 | 0.8188 | 0.027 | 0.036 | 0.440 | 0.019 | 0.039 | 0.637 | 0.095 | 0.077 | 0.219 |
| rs7126398 | 11 | 14268729 | *SPON1* | G | C | 0.08 | 0.06 | 0.96 | 0.041 | | 0.006 | 3E-12 | 0.591 | 0.227 | 0.0091 | -0.074 | 0.078 | 0.344 | -0.087 | 0.087 | 0.318 | 0.012 | 0.148 | 0.934 |
| rs757081 | 11 | 17351683 | *NUCB2* | C | G | 0.34 | 0.33 | 0.83 | 0.024 | | 0.003 | 8E-15 | 0.222 | 0.121 | 0.0677 | 0.013 | 0.038 | 0.727 | 0.030 | 0.041 | 0.471 | -0.097 | 0.083 | 0.245 |
| rs10767838 | 11 | 30347927 | *C11orf46* | A | G | 0.28 | 0.28 | 0.91 | -0.025 | | 0.003 | 3E-14 | -0.275 | 0.124 | 0.0263 | 0.026 | 0.038 | 0.494 | 0.013 | 0.042 | 0.748 | 0.110 | 0.081 | 0.172 |
| rs3802758 | 11 | 45936035 | *PEX16* | G | A | 0.94 | 0.92 | 0.73 | 0.039 | | 0.007 | 2E-09 | 0.772 | 0.227 | 0.0007 | -0.011 | 0.069 | 0.876 | -0.032 | 0.075 | 0.667 | 0.158 | 0.157 | 0.314 |
| rs1681630 | 11 | 47969152 | *PTPRJ* | T | C | 0.66 | 0.69 | 0.76 | -0.029 | | 0.003 | 2E-20 | -0.344 | 0.129 | 0.0078 | -0.019 | 0.040 | 0.642 | -0.025 | 0.044 | 0.566 | 0.024 | 0.085 | 0.774 |
| rs3782089 | 11 | 65336819 | *SSSCA1* | C | T | 0.06 | 0.08 | 0.93 | -0.053 | | 0.007 | 5E-16 | -0.283 | 0.202 | 0.1606 | 0.045 | 0.059 | 0.445 | 0.073 | 0.064 | 0.256 | -0.140 | 0.142 | 0.324 |
| rs7112925 | 11 | 66826160 | *RHOD* | C | T | 0.36 | 0.36 | 0.95 | -0.024 | | 0.003 | 6E-15 | -0.075 | 0.112 | 0.5066 | -0.071 | 0.034 | 0.037 | -0.075 | 0.038 | 0.045 | -0.042 | 0.076 | 0.582 |
| rs2510396 | 11 | 68417652 | *GAL* | G | C | 0.86 | 0.86 | 0.87 | 0.029 | | 0.004 | 9E-12 | 0.369 | 0.159 | 0.02 | -0.009 | 0.049 | 0.847 | -0.018 | 0.054 | 0.737 | 0.055 | 0.107 | 0.607 |
| rs3750972 | 11 | 68830628 | *TPCN2* | T | G | 0.48 | 0.49 | 0.99 | 0.019 | | 0.003 | 1E-09 | 0.234 | 0.105 | 0.0259 | 0.040 | 0.032 | 0.220 | 0.028 | 0.036 | 0.435 | 0.125 | 0.070 | 0.077 |
| rs4357716 | 11 | 69163161 | *MYEOV* | C | T | 0.14 | 0.14 | 1.00 | 0.031 | | 0.005 | 7E-11 | 0.284 | 0.150 | 0.0579 | 0.060 | 0.047 | 0.206 | 0.070 | 0.052 | 0.180 | -0.016 | 0.099 | 0.875 |
| rs11236294 | 11 | 74739934 | *NEU3* | G | T | 0.29 | 0.31 | 0.62 | 0.019 | | 0.003 | 5E-09 | -0.012 | 0.142 | 0.9328 | 0.006 | 0.043 | 0.884 | -0.014 | 0.048 | 0.765 | 0.143 | 0.096 | 0.138 |
| rs606452 | 11 | 75276178 | *SERPINH1* | A | C | 0.86 | 0.82 | 0.54 | -0.043 | | 0.004 | 2E-23 | -0.209 | 0.185 | 0.2582 | 0.061 | 0.057 | 0.282 | 0.096 | 0.062 | 0.123 | -0.177 | 0.127 | 0.163 |
| rs632124 | 11 | 118613235 | *DDX6* | T | A | 0.42 | 0.41 | 0.73 | 0.023 | | 0.003 | 2E-14 | 0.055 | 0.124 | 0.6578 | 0.000 | 0.038 | 0.999 | 0.031 | 0.042 | 0.457 | -0.227 | 0.085 | 0.008 |
| rs10790381 | 11 | 120257495 | *ARHGEF12* | A | G | 0.18 | 0.16 | 0.57 | -0.027 | | 0.004 | 2E-12 | -0.322 | 0.192 | 0.0934 | 0.053 | 0.059 | 0.367 | 0.039 | 0.065 | 0.544 | 0.148 | 0.132 | 0.259 |
| rs1461503 | 11 | 122845075 | *BSX* | A | C | 0.57 | 0.56 | 1.00 | 0.018 | | 0.003 | 5E-10 | 0.064 | 0.106 | 0.5442 | 0.002 | 0.033 | 0.958 | 0.000 | 0.036 | 0.995 | 0.015 | 0.071 | 0.833 |
| rs11221442 | 11 | 128577624 | *FLI1* | G | C | 0.25 | 0.23 | 0.53 | -0.027 | | 0.004 | 3E-14 | 0.003 | 0.172 | 0.9872 | 0.052 | 0.053 | 0.330 | 0.097 | 0.058 | 0.097 | -0.270 | 0.118 | 0.022 |
| rs11612228 | 12 | 576984 | *B4GALNT3* | C | T | 0.38 | 0.36 | 1.00 | 0.02 | | 0.003 | 7E-10 | 0.027 | 0.109 | 0.8057 | 0.019 | 0.034 | 0.580 | 0.019 | 0.038 | 0.613 | 0.020 | 0.073 | 0.780 |
| rs7299326 | 12 | 1573005 | *ERC1* | C | T | 0.05 | 0.06 | 0.86 | -0.041 | | 0.007 | 1E-08 | -0.364 | 0.224 | 0.1049 | 0.009 | 0.069 | 0.891 | -0.004 | 0.077 | 0.963 | 0.094 | 0.145 | 0.516 |
| rs2856321 | 12 | 11855773 | *ETV6* | G | A | 0.64 | 0.66 | 0.97 | -0.031 | | 0.003 | 8E-24 | -0.372 | 0.111 | 0.0008 | -0.036 | 0.034 | 0.283 | -0.025 | 0.037 | 0.501 | -0.114 | 0.075 | 0.126 |
| rs1420023 | 12 | 12876111 | *CDKN1B* | C | G | 0.12 | 0.12 | 0.94 | -0.028 | | 0.005 | 2E-08 | -0.146 | 0.167 | 0.3809 | 0.099 | 0.052 | 0.057 | 0.098 | 0.057 | 0.087 | 0.102 | 0.111 | 0.358 |
| rs4326884 | 12 | 20536371 | *PDE3A* | G | A | 0.51 | 0.51 | 0.87 | 0.019 | | 0.003 | 2E-10 | 0.014 | 0.115 | 0.905 | -0.007 | 0.035 | 0.832 | -0.004 | 0.039 | 0.927 | -0.039 | 0.079 | 0.620 |
| rs11047239 | 12 | 24207780 | *SOX5* | C | G | 0.30 | 0.28 | 0.91 | 0.023 | | 0.003 | 3E-13 | 0.153 | 0.120 | 0.2052 | 0.020 | 0.038 | 0.601 | 0.030 | 0.042 | 0.475 | -0.049 | 0.079 | 0.534 |
| rs1861908 | 12 | 27997409 | *KLHDC5* | C | G | 0.80 | 0.77 | 1.00 | -0.023 | | 0.004 | 1E-08 | -0.069 | 0.125 | 0.5795 | 0.000 | 0.039 | 0.991 | -0.005 | 0.043 | 0.912 | 0.029 | 0.089 | 0.745 |
| rs11049611 | 12 | 28600244 | *CCDC91* | C | T | 0.30 | 0.28 | 1.00 | -0.038 | | 0.003 | 3E-32 | -0.312 | 0.116 | 0.0074 | 0.127 | 0.036 | 0.000 | 0.138 | 0.040 | 0.001 | 0.051 | 0.079 | 0.522 |
| rs12820411 | 12 | 28952342 | *CCDC91* | G | C | 0.32 | 0.33 | 0.93 | -0.021 | | 0.003 | 3E-11 | -0.189 | 0.117 | 0.1052 | 0.031 | 0.036 | 0.386 | 0.047 | 0.040 | 0.241 | -0.068 | 0.077 | 0.373 |
| rs10843390 | 12 | 29496991 | *ERGIC2* | C | T | 0.29 | 0.29 | 0.99 | 0.021 | | 0.003 | 5E-11 | 0.015 | 0.115 | 0.8957 | -0.028 | 0.036 | 0.437 | -0.010 | 0.039 | 0.794 | -0.146 | 0.083 | 0.077 |
| rs10880969 | 12 | 46827023 | *SLC38A2* | T | C | 0.70 | 0.70 | 0.98 | 0.024 | | 0.003 | 6E-13 | 0.095 | 0.116 | 0.4154 | 0.038 | 0.035 | 0.286 | 0.034 | 0.039 | 0.380 | 0.074 | 0.082 | 0.364 |
| rs2306694 | 12 | 56680636 | *CS* | A | G | 0.07 | 0.06 | 1.00 | 0.046 | | 0.006 | 2E-15 | 0.183 | 0.212 | 0.3884 | 0.014 | 0.070 | 0.844 | 0.049 | 0.075 | 0.516 | -0.260 | 0.177 | 0.143 |
| rs10877030 | 12 | 58256714 | *CTDSP2* | T | G | 0.32 | 0.30 | 0.93 | -0.023 | | 0.003 | 4E-13 | -0.254 | 0.117 | 0.0299 | -0.035 | 0.036 | 0.321 | -0.026 | 0.039 | 0.500 | -0.097 | 0.080 | 0.225 |
| rs17122659 | 12 | 59956923 | *SLC16A7* | A | G | 0.12 | 0.10 | 0.81 | 0.031 | | 0.005 | 5E-10 | 0.312 | 0.192 | 0.1036 | 0.101 | 0.057 | 0.076 | 0.122 | 0.062 | 0.050 | -0.062 | 0.128 | 0.626 |
| rs2164968 | 12 | 65677086 | *MSRB3* | T | C | 0.38 | 0.36 | 1.00 | 0.018 | | 0.003 | 1E-08 | 0.000 | 0.109 | 0.9995 | 0.021 | 0.033 | 0.513 | 0.007 | 0.036 | 0.848 | 0.113 | 0.071 | 0.114 |
| rs8756 | 12 | 66359752 | *HMGA2* | C | A | 0.51 | 0.55 | 1.00 | -0.059 | | 0.003 | 5E-90 | -0.524 | 0.104 | <.0001 | 0.043 | 0.032 | 0.187 | 0.038 | 0.035 | 0.284 | 0.074 | 0.073 | 0.309 |
| rs11175992 | 12 | 66391396 | *HMGA2* | T | A | 0.28 | 0.28 | 0.64 | -0.048 | | 0.004 | 1E-33 | -0.318 | 0.148 | 0.0314 | 0.014 | 0.045 | 0.758 | 0.015 | 0.049 | 0.756 | 0.010 | 0.103 | 0.926 |
| rs10748128 | 12 | 69827658 | *FRS2* | G | T | 0.35 | 0.35 | 0.64 | 0.038 | | 0.003 | 4E-29 | 0.354 | 0.136 | 0.009 | -0.051 | 0.041 | 0.222 | -0.055 | 0.046 | 0.229 | -0.015 | 0.093 | 0.875 |
| rs17783015 | 12 | 90231386 | *ATP2B1* | C | T | 0.16 | 0.14 | 0.82 | -0.023 | | 0.004 | 2E-08 | -0.053 | 0.163 | 0.7453 | -0.007 | 0.050 | 0.890 | -0.029 | 0.055 | 0.596 | 0.135 | 0.110 | 0.219 |
| rs11107062 | 12 | 93919840 | *MRPL42* | C | T | 0.21 | 0.17 | 0.74 | 0.028 | | 0.004 | 1E-11 | 0.261 | 0.157 | 0.0971 | 0.006 | 0.049 | 0.896 | 0.021 | 0.054 | 0.690 | -0.105 | 0.115 | 0.362 |
| rs3825199 | 12 | 93976954 | *SOCS2* | A | G | 0.23 | 0.21 | 1.00 | 0.051 | | 0.004 | 4E-49 | 0.264 | 0.128 | 0.0387 | -0.006 | 0.039 | 0.874 | 0.003 | 0.043 | 0.950 | -0.067 | 0.089 | 0.454 |
| rs10859567 | 12 | 94126925 | *CRADD* | T | G | 0.44 | 0.42 | 0.96 | -0.035 | | 0.003 | 5E-33 | -0.160 | 0.108 | 0.1377 | -0.029 | 0.033 | 0.372 | -0.025 | 0.036 | 0.488 | -0.062 | 0.075 | 0.404 |
| rs7971536 | 12 | 102373788 | *CCDC53* | T | A | 0.46 | 0.48 | 0.63 | -0.029 | | 0.003 | 2E-19 | -0.100 | 0.129 | 0.437 | 0.034 | 0.040 | 0.390 | 0.041 | 0.044 | 0.351 | -0.015 | 0.092 | 0.869 |
| rs833706 | 12 | 103062597 | *PAH* | A | G | 0.25 | 0.25 | 0.97 | -0.021 | | 0.004 | 3E-09 | 0.036 | 0.123 | 0.7706 | -0.016 | 0.038 | 0.666 | 0.003 | 0.041 | 0.936 | -0.145 | 0.084 | 0.083 |
| rs2164747 | 12 | 104344836 | *HSP90B1* | A | G | 0.10 | 0.12 | 0.99 | 0.029 | | 0.005 | 5E-09 | -0.016 | 0.164 | 0.9213 | -0.062 | 0.050 | 0.219 | -0.056 | 0.055 | 0.310 | -0.103 | 0.113 | 0.364 |
| rs2888893 | 12 | 107338631 | *C12orf23* | C | T | 0.49 | 0.54 | 0.99 | -0.017 | | 0.003 | 9E-09 | -0.080 | 0.106 | 0.4499 | 0.014 | 0.032 | 0.664 | 0.009 | 0.036 | 0.799 | 0.044 | 0.072 | 0.541 |
| rs11616067 | 12 | 116393174 | *MED13L* | A | G | 0.24 | 0.23 | 0.96 | -0.021 | | 0.004 | 8E-09 | 0.008 | 0.125 | 0.9478 | 0.021 | 0.037 | 0.566 | -0.005 | 0.041 | 0.910 | 0.197 | 0.079 | 0.013 |
| rs4767473 | 12 | 117365506 | *FBXW8* | G | A | 0.86 | 0.88 | 0.75 | 0.025 | | 0.004 | 3E-08 | -0.139 | 0.186 | 0.4563 | 0.006 | 0.056 | 0.908 | -0.014 | 0.061 | 0.817 | 0.146 | 0.129 | 0.256 |
| rs497273 | 12 | 121204682 | *SPPL3* | C | G | 0.62 | 0.66 | 0.99 | -0.018 | | 0.003 | 7E-09 | -0.123 | 0.110 | 0.2621 | 0.035 | 0.035 | 0.319 | 0.025 | 0.038 | 0.522 | 0.095 | 0.076 | 0.215 |
| rs11835818 | 12 | 122494809 | *BCL7A* | T | C | 0.49 | 0.47 | 0.62 | 0.022 | | 0.003 | 2E-13 | 0.183 | 0.133 | 0.1677 | 0.004 | 0.041 | 0.930 | 0.015 | 0.045 | 0.744 | -0.070 | 0.090 | 0.437 |
| rs7980687 | 12 | 123822711 | *SBNO1* | G | A | 0.20 | 0.21 | 0.97 | 0.039 | | 0.004 | 1E-26 | 0.200 | 0.129 | 0.1203 | 0.003 | 0.040 | 0.934 | -0.001 | 0.045 | 0.989 | 0.030 | 0.087 | 0.731 |
| rs11057552 | 12 | 124750895 | *FAM101A* | T | A | 0.18 | 0.18 | 0.99 | 0.028 | | 0.004 | 4E-13 | 0.386 | 0.136 | 0.0047 | 0.071 | 0.042 | 0.090 | 0.068 | 0.046 | 0.136 | 0.093 | 0.097 | 0.336 |
| rs1809889 | 12 | 124801226 | *FAM101A* | T | C | 0.71 | 0.73 | 0.94 | -0.032 | | 0.003 | 4E-21 | -0.367 | 0.120 | 0.0023 | -0.013 | 0.038 | 0.732 | 0.006 | 0.042 | 0.878 | -0.136 | 0.083 | 0.103 |
| rs1199734 | 13 | 21570246 | *LATS2* | T | G | 0.81 | 0.80 | 0.58 | 0.022 | | 0.004 | 2E-08 | -0.134 | 0.175 | 0.4439 | 0.024 | 0.052 | 0.639 | 0.041 | 0.057 | 0.467 | -0.110 | 0.114 | 0.337 |
| rs11618507 | 13 | 30172751 | *SLC7A1* | G | T | 0.25 | 0.20 | 1.00 | 0.023 | | 0.004 | 3E-10 | 0.226 | 0.128 | 0.0788 | 0.065 | 0.040 | 0.107 | 0.072 | 0.045 | 0.104 | 0.023 | 0.088 | 0.796 |
| rs12323101 | 13 | 33143406 | *PDS5B* | G | A | 0.37 | 0.35 | 1.00 | 0.021 | | 0.003 | 1E-11 | 0.343 | 0.109 | 0.0017 | 0.065 | 0.034 | 0.058 | 0.076 | 0.037 | 0.043 | -0.012 | 0.082 | 0.880 |
| rs12863103 | 13 | 33723244 | *STARD13* | C | T | 0.29 | 0.30 | 1.00 | -0.019 | | 0.003 | 2E-09 | -0.246 | 0.115 | 0.0322 | 0.056 | 0.036 | 0.120 | 0.040 | 0.040 | 0.310 | 0.163 | 0.080 | 0.042 |
| rs7334755 | 13 | 50469913 | *C13orf1* | C | T | 0.19 | 0.20 | 1.00 | -0.032 | | 0.004 | 9E-15 | -0.092 | 0.132 | 0.4868 | -0.008 | 0.040 | 0.845 | -0.005 | 0.044 | 0.912 | -0.029 | 0.089 | 0.740 |
| rs2687950 | 13 | 50718468 | *KCNRG* | C | T | 0.25 | 0.25 | 0.87 | 0.036 | | 0.003 | 9E-27 | 0.285 | 0.128 | 0.0265 | -0.008 | 0.040 | 0.837 | -0.015 | 0.045 | 0.736 | 0.031 | 0.084 | 0.713 |
| rs1753637 | 13 | 51084173 | *DLEU7* | T | G | 0.69 | 0.71 | 0.99 | -0.036 | | 0.003 | 1E-29 | -0.160 | 0.116 | 0.1673 | 0.004 | 0.035 | 0.918 | 0.016 | 0.038 | 0.681 | -0.082 | 0.077 | 0.285 |
| rs3118905 | 13 | 51105334 | *DLEU7* | G | A | 0.28 | 0.29 | 0.97 | -0.058 | | 0.003 | 1E-69 | -0.344 | 0.118 | 0.0036 | 0.046 | 0.036 | 0.204 | 0.037 | 0.040 | 0.351 | 0.102 | 0.076 | 0.179 |
| rs4883972 | 13 | 75058481 | *KLF12* | C | G | 0.45 | 0.50 | 0.73 | -0.019 | | 0.003 | 3E-10 | 0.031 | 0.121 | 0.7951 | -0.009 | 0.036 | 0.801 | -0.024 | 0.040 | 0.539 | 0.097 | 0.084 | 0.248 |
| rs3818416 | 13 | 78474468 | *EDNRB* | A | C | 0.77 | 0.75 | 0.94 | 0.021 | | 0.004 | 2E-09 | 0.174 | 0.125 | 0.1657 | 0.046 | 0.039 | 0.241 | 0.044 | 0.043 | 0.308 | 0.050 | 0.087 | 0.567 |
| rs11616380 | 13 | 80705315 | *SPRY2* | G | T | 0.28 | 0.29 | 0.85 | 0.019 | | 0.003 | 1E-08 | 0.202 | 0.123 | 0.1006 | -0.053 | 0.039 | 0.173 | -0.034 | 0.043 | 0.423 | -0.173 | 0.081 | 0.032 |
| rs6563199 | 13 | 81550449 | *SPRY2* | T | C | 0.65 | 0.65 | 0.92 | -0.018 | | 0.003 | 1E-08 | -0.330 | 0.113 | 0.0034 | 0.019 | 0.035 | 0.595 | 0.014 | 0.039 | 0.717 | 0.049 | 0.078 | 0.531 |
| rs7319045 | 13 | 92024574 | *GPC5* | A | G | 0.61 | 0.61 | 0.92 | -0.024 | | 0.003 | 8E-15 | 0.042 | 0.112 | 0.7107 | 0.084 | 0.035 | 0.017 | 0.086 | 0.039 | 0.027 | 0.068 | 0.076 | 0.372 |
| rs7985356 | 13 | 115027462 | *CDC16* | T | A | 0.23 | 0.23 | 1.00 | -0.023 | | 0.003 | 5E-11 | -0.081 | 0.124 | 0.5128 | -0.070 | 0.038 | 0.065 | -0.085 | 0.042 | 0.040 | 0.030 | 0.085 | 0.726 |
| rs8017130 | 14 | 23759156 | *HOMEZ* | A | G | 0.69 | 0.67 | 0.80 | 0.023 | | 0.003 | 1E-11 | 0.100 | 0.125 | 0.4208 | -0.035 | 0.037 | 0.350 | -0.032 | 0.042 | 0.440 | -0.053 | 0.079 | 0.498 |
| rs1950500 | 14 | 24830850 | *NFATC4* | T | C | 0.70 | 0.70 | 0.65 | -0.031 | | 0.003 | 3E-22 | -0.250 | 0.140 | 0.0738 | -0.020 | 0.044 | 0.647 | -0.012 | 0.049 | 0.801 | -0.074 | 0.093 | 0.424 |
| rs12435366 | 14 | 35838389 | *NFKBIA* | C | T | 0.27 | 0.23 | 0.94 | -0.023 | | 0.004 | 5E-11 | -0.104 | 0.129 | 0.4213 | 0.056 | 0.038 | 0.143 | 0.072 | 0.041 | 0.083 | -0.060 | 0.095 | 0.528 |
| rs10131337 | 14 | 37144516 | *PAX9* | C | T | 0.24 | 0.23 | 0.51 | 0.027 | | 0.004 | 3E-12 | 0.587 | 0.173 | 0.0007 | 0.047 | 0.052 | 0.362 | 0.037 | 0.057 | 0.513 | 0.116 | 0.115 | 0.317 |
| rs4901537 | 14 | 55203126 | *SAMD4A* | G | C | 0.74 | 0.81 | 0.65 | -0.022 | | 0.004 | 2E-09 | 0.082 | 0.164 | 0.6173 | -0.067 | 0.050 | 0.180 | -0.069 | 0.055 | 0.214 | -0.061 | 0.112 | 0.587 |
| rs11624136 | 14 | 59688820 | *DAAM1* | G | A | 0.50 | 0.51 | 0.97 | 0.018 | | 0.003 | 2E-09 | 0.060 | 0.106 | 0.5738 | -0.005 | 0.034 | 0.887 | -0.002 | 0.037 | 0.958 | -0.019 | 0.074 | 0.800 |
| rs2093210 | 14 | 60957279 | *C14orf39* | C | T | 0.58 | 0.58 | 0.98 | -0.039 | | 0.003 | 3E-35 | -0.139 | 0.107 | 0.1938 | 0.024 | 0.034 | 0.469 | 0.025 | 0.037 | 0.502 | 0.029 | 0.072 | 0.684 |
| rs2781373 | 14 | 65568215 | *MAX* | G | A | 0.38 | 0.35 | 0.63 | -0.021 | | 0.003 | 4E-12 | -0.006 | 0.138 | 0.9652 | 0.046 | 0.043 | 0.283 | 0.060 | 0.047 | 0.204 | -0.045 | 0.094 | 0.636 |
| rs1980850 | 14 | 68647188 | *RAD51L1* | A | G | 0.83 | 0.80 | 1.00 | 0.03 | | 0.004 | 4E-14 | 0.440 | 0.133 | 0.001 | -0.024 | 0.039 | 0.541 | -0.023 | 0.043 | 0.587 | -0.019 | 0.085 | 0.820 |
| rs2058092 | 14 | 73932966 | *NUMB* | T | C | 0.44 | 0.47 | 0.71 | -0.017 | | 0.003 | 1E-08 | -0.249 | 0.126 | 0.0474 | 0.061 | 0.039 | 0.116 | 0.060 | 0.043 | 0.161 | 0.078 | 0.086 | 0.364 |
| rs862034 | 14 | 74990746 | *LTBP2* | A | G | 0.64 | 0.64 | 0.78 | 0.028 | | 0.003 | 6E-20 | 0.212 | 0.123 | 0.0835 | -0.028 | 0.039 | 0.460 | -0.035 | 0.042 | 0.414 | 0.012 | 0.086 | 0.889 |
| rs7154721 | 14 | 92427348 | *TRIP11* | T | C | 0.43 | 0.43 | 1.00 | -0.027 | | 0.003 | 5E-20 | -0.177 | 0.105 | 0.0937 | 0.005 | 0.033 | 0.878 | 0.017 | 0.036 | 0.641 | -0.068 | 0.071 | 0.344 |
| rs1190545 | 14 | 102904179 | *KIAA0329* | G | C | 0.74 | 0.74 | 0.98 | 0.025 | | 0.003 | 4E-14 | -0.031 | 0.120 | 0.7978 | -0.039 | 0.037 | 0.297 | -0.037 | 0.041 | 0.370 | -0.056 | 0.086 | 0.513 |
| rs12882130 | 14 | 103878774 | *MARK3* | C | G | 0.37 | 0.36 | 0.63 | -0.025 | | 0.003 | 5E-15 | 0.050 | 0.138 | 0.716 | 0.001 | 0.042 | 0.987 | -0.012 | 0.046 | 0.794 | 0.091 | 0.092 | 0.328 |
| rs10152739 | 15 | 38483866 | *SPRED1* | A | T | 0.25 | 0.27 | 0.99 | 0.022 | | 0.003 | 1E-10 | 0.190 | 0.120 | 0.1129 | 0.001 | 0.037 | 0.981 | 0.022 | 0.040 | 0.585 | -0.154 | 0.084 | 0.068 |
| rs316618 | 15 | 41796498 | *LTK* | T | A | 0.22 | 0.22 | 0.97 | -0.026 | | 0.004 | 3E-12 | -0.286 | 0.126 | 0.0234 | -0.002 | 0.039 | 0.956 | 0.022 | 0.042 | 0.611 | -0.168 | 0.088 | 0.056 |
| rs10744956 | 15 | 51269629 | *AP4E1* | A | G | 0.78 | 0.80 | 0.98 | 0.028 | | 0.004 | 4E-15 | -0.091 | 0.131 | 0.4889 | -0.020 | 0.039 | 0.613 | -0.024 | 0.043 | 0.579 | -0.001 | 0.086 | 0.994 |
| rs16964211 | 15 | 51530495 | *CYP19A1* | G | A | 0.05 | 0.06 | 0.99 | -0.057 | | 0.007 | 1E-15 | -0.191 | 0.227 | 0.3992 | 0.027 | 0.069 | 0.700 | -0.016 | 0.077 | 0.835 | 0.284 | 0.139 | 0.041 |
| rs782930 | 15 | 61408362 | *RORA* | G | A | 0.35 | 0.33 | 0.93 | -0.02 | | 0.003 | 3E-10 | -0.395 | 0.113 | 0.0005 | 0.045 | 0.034 | 0.182 | 0.036 | 0.037 | 0.330 | 0.103 | 0.072 | 0.151 |
| rs7177711 | 15 | 62379971 | *FAM148A* | A | G | 0.46 | 0.44 | 0.61 | -0.021 | | 0.003 | 5E-13 | 0.022 | 0.134 | 0.8714 | 0.006 | 0.041 | 0.886 | 0.005 | 0.045 | 0.910 | 0.011 | 0.096 | 0.907 |
| rs7162825 | 15 | 63439186 | *LACTB* | C | T | 0.50 | 0.50 | 0.98 | 0.016 | | 0.003 | 4E-08 | 0.035 | 0.105 | 0.7419 | -0.035 | 0.032 | 0.279 | -0.054 | 0.035 | 0.122 | 0.107 | 0.076 | 0.159 |
| rs10152591 | 15 | 70048157 | *TLE3* | A | C | 0.10 | 0.09 | 0.96 | -0.044 | | 0.005 | 2E-17 | -0.182 | 0.186 | 0.3287 | 0.006 | 0.060 | 0.925 | 0.004 | 0.067 | 0.952 | 0.009 | 0.125 | 0.941 |
| rs975210 | 15 | 70364352 | *TLE3* | G | A | 0.18 | 0.16 | 1.00 | 0.035 | | 0.004 | 1E-17 | 0.309 | 0.142 | 0.03 | -0.013 | 0.045 | 0.775 | 0.006 | 0.050 | 0.905 | -0.144 | 0.096 | 0.134 |
| rs11634405 | 15 | 72084693 | *THSD4* | A | G | 0.64 | 0.62 | 0.70 | -0.02 | | 0.003 | 4E-10 | -0.177 | 0.130 | 0.1713 | 0.058 | 0.040 | 0.148 | 0.060 | 0.044 | 0.169 | 0.036 | 0.088 | 0.686 |
| rs12904334 | 15 | 72842705 | *ARIH1* | G | A | 0.02 | 0.02 | 0.87 | 0.084 | | 0.013 | 4E-11 | 1.033 | 0.441 | 0.0193 | -0.053 | 0.139 | 0.702 | -0.075 | 0.153 | 0.623 | 0.110 | 0.309 | 0.722 |
| rs4337252 | 15 | 74226765 | *LOXL1* | G | C | 0.50 | 0.48 | 1.00 | 0.026 | | 0.003 | 3E-19 | 0.304 | 0.103 | 0.0031 | -0.016 | 0.032 | 0.624 | -0.007 | 0.036 | 0.853 | -0.083 | 0.070 | 0.235 |
| rs5742915 | 15 | 74336633 | *PML* | T | C | 0.47 | 0.45 | 1.00 | 0.035 | | 0.003 | 2E-29 | 0.041 | 0.104 | 0.6924 | -0.013 | 0.033 | 0.699 | -0.011 | 0.036 | 0.750 | -0.022 | 0.072 | 0.760 |
| rs16968242 | 15 | 76740219 | *SCAPER* | C | G | 0.07 | 0.07 | 0.95 | 0.035 | | 0.006 | 2E-09 | -0.107 | 0.208 | 0.6081 | 0.010 | 0.064 | 0.878 | 0.013 | 0.071 | 0.851 | -0.021 | 0.131 | 0.875 |
| rs12914466 | 15 | 81836638 | *TMC3* | G | A | 0.62 | 0.60 | 0.56 | 0.017 | | 0.003 | 1E-08 | 0.135 | 0.142 | 0.3436 | 0.015 | 0.043 | 0.726 | 0.003 | 0.047 | 0.955 | 0.099 | 0.095 | 0.298 |
| rs17349981 | 15 | 82231920 | *MEX3B* | A | T | 0.15 | 0.12 | 0.63 | -0.025 | | 0.004 | 4E-09 | -0.146 | 0.195 | 0.4535 | 0.118 | 0.061 | 0.054 | 0.111 | 0.067 | 0.096 | 0.149 | 0.141 | 0.290 |
| rs2257011 | 15 | 84266145 | *SH3GL3* | G | T | 0.50 | 0.49 | 0.86 | 0.044 | | 0.003 | 1E-47 | 0.262 | 0.111 | 0.0187 | -0.023 | 0.035 | 0.518 | -0.012 | 0.039 | 0.762 | -0.103 | 0.076 | 0.171 |
| rs7162542 | 15 | 84514290 | *ADAMTSL3* | C | G | 0.55 | 0.57 | 0.99 | 0.046 | | 0.003 | 8E-55 | 0.290 | 0.105 | 0.0056 | -0.019 | 0.032 | 0.551 | -0.002 | 0.036 | 0.952 | -0.143 | 0.070 | 0.042 |
| rs11855014 | 15 | 85728834 | *PDE8A* | G | A | 0.29 | 0.31 | 0.83 | -0.022 | | 0.003 | 9E-11 | 0.124 | 0.124 | 0.3201 | 0.038 | 0.037 | 0.301 | 0.055 | 0.041 | 0.176 | -0.080 | 0.084 | 0.340 |
| rs11633371 | 15 | 89356832 | *ACAN* | G | T | 0.49 | 0.48 | 0.96 | 0.028 | | 0.003 | 1E-20 | 0.280 | 0.106 | 0.0086 | -0.027 | 0.032 | 0.401 | -0.029 | 0.036 | 0.410 | -0.013 | 0.074 | 0.859 |
| rs16942341 | 15 | 89388905 | *ACAN* | C | T | 0.03 | 0.03 | 0.60 | -0.138 | | 0.01 | 3E-43 | -1.584 | 0.403 | <.0001 | 0.255 | 0.123 | 0.039 | 0.226 | 0.139 | 0.103 | 0.415 | 0.248 | 0.095 |
| rs3817428 | 15 | 89415247 | *ACAN* | C | G | 0.28 | 0.22 | 0.51 | -0.039 | | 0.003 | 2E-31 | -0.089 | 0.172 | 0.6045 | 0.144 | 0.053 | 0.006 | 0.149 | 0.058 | 0.010 | 0.112 | 0.121 | 0.353 |
| rs2238300 | 15 | 89851580 | *FANCI* | G | A | 0.40 | 0.38 | 1.00 | -0.021 | | 0.003 | 2E-12 | 0.042 | 0.107 | 0.6934 | -0.004 | 0.033 | 0.908 | 0.004 | 0.036 | 0.908 | -0.061 | 0.074 | 0.408 |
| rs7181724 | 15 | 94551607 | *MCTP2* | A | G | 0.45 | 0.45 | 0.68 | 0.02 | | 0.003 | 1E-10 | -0.051 | 0.127 | 0.6884 | -0.060 | 0.040 | 0.134 | -0.063 | 0.044 | 0.151 | -0.034 | 0.084 | 0.682 |
| rs2871865 | 15 | 99194896 | *IGF1R* | C | G | 0.12 | 0.12 | 0.89 | -0.062 | | 0.005 | 2E-34 | -0.625 | 0.172 | 0.0003 | -0.044 | 0.051 | 0.392 | -0.030 | 0.056 | 0.589 | -0.141 | 0.115 | 0.221 |
| rs2573625 | 15 | 100513158 | *ADAMTS17* | C | T | 0.67 | 0.64 | 0.77 | 0.029 | | 0.003 | 6E-20 | 0.220 | 0.127 | 0.0825 | 0.023 | 0.038 | 0.543 | 0.021 | 0.041 | 0.617 | 0.034 | 0.082 | 0.683 |
| rs4246302 | 15 | 100687967 | *ADAMTS17* | A | G | 0.32 | 0.31 | 0.80 | 0.027 | | 0.003 | 2E-16 | 0.148 | 0.125 | 0.2363 | 0.009 | 0.038 | 0.819 | 0.012 | 0.042 | 0.776 | -0.023 | 0.082 | 0.778 |
| rs4548838 | 15 | 100761190 | *ADAMTS17* | T | C | 0.54 | 0.55 | 0.94 | -0.033 | | 0.003 | 9E-28 | -0.405 | 0.108 | 0.0002 | -0.011 | 0.034 | 0.738 | -0.003 | 0.037 | 0.932 | -0.072 | 0.073 | 0.323 |
| rs8042424 | 15 | 101762539 | *CHSY1* | C | T | 0.26 | 0.24 | 0.82 | -0.023 | | 0.004 | 8E-11 | -0.162 | 0.135 | 0.2306 | 0.001 | 0.041 | 0.983 | -0.013 | 0.045 | 0.773 | 0.104 | 0.089 | 0.245 |
| rs11648796 | 16 | 792190 | *NARFL* | A | G | 0.25 | 0.29 | 0.72 | 0.033 | | 0.004 | 1E-18 | 0.135 | 0.135 | 0.3206 | 0.003 | 0.042 | 0.938 | -0.001 | 0.047 | 0.989 | 0.028 | 0.092 | 0.761 |
| rs12597498 | 16 | 990815 | *LMF1* | C | T | 0.37 | 0.37 | 0.99 | 0.018 | | 0.003 | 8E-09 | 0.304 | 0.109 | 0.0051 | 0.014 | 0.033 | 0.664 | 0.005 | 0.036 | 0.893 | 0.080 | 0.074 | 0.284 |
| rs26868 | 16 | 2249376 | *CASKIN1* | T | A | 0.47 | 0.41 | 0.89 | 0.029 | | 0.003 | 3E-18 | 0.199 | 0.113 | 0.0786 | -0.013 | 0.035 | 0.720 | -0.026 | 0.039 | 0.499 | 0.072 | 0.078 | 0.357 |
| rs2014467 | 16 | 2336394 | *ABCA3* | T | C | 0.34 | 0.35 | 0.55 | 0.019 | | 0.003 | 2E-09 | 0.174 | 0.146 | 0.2324 | -0.002 | 0.045 | 0.960 | -0.005 | 0.050 | 0.925 | 0.010 | 0.097 | 0.914 |
| rs12926008 | 16 | 2488211 | *CCNF* | T | C | 0.69 | 0.65 | 0.53 | 0.02 | | 0.003 | 3E-10 | 0.336 | 0.149 | 0.0242 | 0.040 | 0.045 | 0.384 | 0.066 | 0.050 | 0.189 | -0.143 | 0.098 | 0.142 |
| rs129963 | 16 | 3796147 | *CREBBP* | T | C | 0.58 | 0.54 | 1.00 | 0.021 | | 0.003 | 3E-10 | 0.065 | 0.104 | 0.5305 | 0.018 | 0.033 | 0.589 | 0.009 | 0.037 | 0.815 | 0.082 | 0.071 | 0.246 |
| rs2531992 | 16 | 4021734 | *ADCY9* | A | G | 0.85 | 0.83 | 0.78 | 0.027 | | 0.004 | 1E-10 | 0.408 | 0.161 | 0.011 | -0.061 | 0.048 | 0.204 | -0.082 | 0.053 | 0.121 | 0.069 | 0.110 | 0.527 |
| rs960006 | 16 | 4911195 | *UBN1* | T | C | 0.47 | 0.47 | 0.54 | 0.02 | | 0.003 | 1E-09 | 0.067 | 0.145 | 0.6427 | 0.010 | 0.044 | 0.813 | 0.006 | 0.048 | 0.903 | 0.038 | 0.099 | 0.706 |
| rs1659127 | 16 | 14388305 | *MKL2* | G | A | 0.34 | 0.33 | 1.00 | 0.03 | | 0.003 | 3E-19 | 0.134 | 0.112 | 0.2317 | -0.011 | 0.034 | 0.752 | -0.032 | 0.038 | 0.402 | 0.125 | 0.074 | 0.088 |
| rs2023693 | 16 | 20880040 | *DCUN1D3* | A | G | 0.60 | 0.57 | 0.76 | 0.017 | | 0.003 | 2E-08 | 0.265 | 0.121 | 0.0293 | 0.014 | 0.038 | 0.708 | 0.003 | 0.042 | 0.938 | 0.082 | 0.086 | 0.337 |
| rs11642612 | 16 | 30030195 | *FLJ25404* | A | C | 0.40 | 0.39 | 1.00 | 0.016 | | 0.003 | 4E-08 | 0.021 | 0.107 | 0.8466 | -0.013 | 0.033 | 0.686 | -0.019 | 0.036 | 0.603 | 0.027 | 0.073 | 0.711 |
| rs4785393 | 16 | 50259483 | *PAPD5* | A | G | 0.16 | 0.19 | 0.93 | 0.023 | | 0.004 | 1E-08 | -0.246 | 0.137 | 0.0726 | -0.011 | 0.042 | 0.789 | -0.023 | 0.046 | 0.621 | 0.074 | 0.088 | 0.401 |
| rs8058684 | 16 | 53515118 | *RBL2* | G | A | 0.31 | 0.32 | 1.00 | 0.021 | | 0.003 | 1E-10 | 0.061 | 0.113 | 0.5915 | -0.033 | 0.035 | 0.347 | -0.043 | 0.039 | 0.266 | 0.049 | 0.074 | 0.512 |
| rs1966913 | 16 | 67384226 | *LRRC36* | A | T | 0.04 | 0.05 | 0.85 | -0.044 | | 0.007 | 1E-09 | -0.572 | 0.267 | 0.0321 | -0.100 | 0.083 | 0.226 | -0.119 | 0.092 | 0.193 | 0.032 | 0.176 | 0.857 |
| rs3790086 | 16 | 69887707 | *WWP2* | C | G | 0.44 | 0.42 | 0.91 | -0.023 | | 0.003 | 3E-15 | -0.170 | 0.110 | 0.1221 | 0.039 | 0.034 | 0.244 | 0.049 | 0.037 | 0.185 | -0.028 | 0.076 | 0.712 |
| rs217181 | 16 | 72114002 | *HPR* | C | T | 0.20 | 0.19 | 1.00 | 0.024 | | 0.004 | 4E-10 | 0.260 | 0.131 | 0.0467 | 0.067 | 0.040 | 0.093 | 0.077 | 0.044 | 0.079 | 0.001 | 0.092 | 0.991 |
| rs11640018 | 16 | 75328308 | *CFDP1* | T | C | 0.37 | 0.38 | 0.55 | 0.019 | | 0.003 | 2E-09 | 0.059 | 0.143 | 0.6795 | 0.043 | 0.046 | 0.350 | 0.041 | 0.051 | 0.413 | 0.053 | 0.092 | 0.570 |
| rs4243206 | 16 | 81589983 | *CMIP* | A | C | 0.81 | 0.81 | 0.61 | -0.024 | | 0.004 | 2E-09 | -0.125 | 0.173 | 0.4702 | 0.030 | 0.053 | 0.574 | 0.034 | 0.059 | 0.567 | 0.013 | 0.113 | 0.906 |
| rs6420435 | 16 | 82184201 | *MPHOSPH6* | A | C | 0.79 | 0.75 | 0.91 | -0.023 | | 0.004 | 3E-10 | 0.078 | 0.126 | 0.5373 | -0.036 | 0.039 | 0.356 | -0.041 | 0.042 | 0.334 | 0.000 | 0.083 | 0.996 |
| rs2326458 | 16 | 84987679 | *ZDHHC7* | C | A | 0.75 | 0.74 | 0.95 | -0.022 | | 0.004 | 5E-10 | -0.166 | 0.123 | 0.1768 | 0.004 | 0.035 | 0.911 | -0.010 | 0.039 | 0.797 | 0.105 | 0.086 | 0.220 |
| rs4843367 | 16 | 86417890 | *FOXF1* | C | T | 0.34 | 0.36 | 0.74 | -0.019 | | 0.003 | 2E-09 | -0.095 | 0.125 | 0.4477 | -0.035 | 0.039 | 0.370 | -0.055 | 0.044 | 0.211 | 0.097 | 0.082 | 0.238 |
| rs8052560 | 16 | 88777242 | *C16orf84* | C | A | 0.79 | 0.80 | 1.00 | 0.036 | | 0.004 | 4E-16 | 0.129 | 0.129 | 0.317 | 0.023 | 0.041 | 0.574 | 0.032 | 0.045 | 0.477 | -0.035 | 0.090 | 0.697 |
| rs2377058 | 16 | 89734831 | *C16orf55* | A | G | 0.34 | 0.35 | 1.00 | 0.017 | | 0.003 | 4E-08 | 0.077 | 0.108 | 0.4773 | -0.005 | 0.034 | 0.874 | 0.000 | 0.037 | 0.998 | -0.040 | 0.072 | 0.577 |
| rs870183 | 17 | 599811 | *VPS53* | A | G | 0.53 | 0.54 | 1.00 | 0.017 | | 0.003 | 6E-09 | 0.036 | 0.104 | 0.7303 | 0.042 | 0.032 | 0.199 | 0.028 | 0.036 | 0.433 | 0.134 | 0.073 | 0.065 |
| rs9217 | 17 | 7363088 | *ZBTB4* | T | C | 0.37 | 0.35 | 1.00 | 0.028 | | 0.003 | 5E-20 | 0.434 | 0.109 | <.0001 | -0.061 | 0.033 | 0.067 | -0.077 | 0.037 | 0.037 | 0.040 | 0.075 | 0.587 |
| rs1625895 | 17 | 7578115 | *TP53* | T | C | 0.88 | 0.86 | 0.99 | 0.03 | | 0.005 | 4E-10 | 0.136 | 0.153 | 0.3754 | -0.025 | 0.046 | 0.586 | -0.025 | 0.051 | 0.623 | -0.023 | 0.102 | 0.823 |
| rs8067165 | 17 | 8031936 | *HES7* | C | G | 0.60 | 0.58 | 1.00 | 0.023 | | 0.003 | 7E-12 | 0.151 | 0.106 | 0.1531 | 0.015 | 0.033 | 0.641 | 0.016 | 0.036 | 0.656 | 0.011 | 0.070 | 0.881 |
| rs4640244 | 17 | 21284223 | *KCNJ12* | A | G | 0.39 | 0.34 | 0.56 | -0.025 | | 0.003 | 2E-13 | 0.049 | 0.149 | 0.7396 | -0.025 | 0.045 | 0.579 | -0.005 | 0.049 | 0.923 | -0.161 | 0.105 | 0.124 |
| rs3809790 | 17 | 27955540 | *SSH2* | C | T | 0.47 | 0.46 | 0.75 | -0.016 | | 0.003 | 5E-08 | 0.006 | 0.123 | 0.9578 | 0.000 | 0.036 | 0.989 | -0.009 | 0.040 | 0.818 | 0.069 | 0.078 | 0.379 |
| rs3760318 | 17 | 29247715 | *CENTA2* | G | A | 0.37 | 0.37 | 1.00 | -0.041 | | 0.003 | 3E-41 | -0.384 | 0.107 | 0.0003 | -0.043 | 0.033 | 0.188 | -0.045 | 0.036 | 0.205 | -0.033 | 0.074 | 0.657 |
| rs2028067 | 17 | 30239698 | *UTP6* | T | C | 0.17 | 0.20 | 0.60 | 0.031 | | 0.004 | 8E-16 | 0.121 | 0.168 | 0.4742 | 0.024 | 0.050 | 0.637 | 0.023 | 0.055 | 0.674 | 0.024 | 0.113 | 0.833 |
| rs2338115 | 17 | 36929578 | *PIP4K2B* | C | T | 0.54 | 0.52 | 0.71 | 0.023 | | 0.003 | 1E-14 | -0.312 | 0.126 | 0.013 | 0.002 | 0.037 | 0.961 | 0.022 | 0.041 | 0.587 | -0.143 | 0.086 | 0.096 |
| rs584828 | 17 | 38599230 | *IGFBP4* | C | T | 0.40 | 0.39 | 0.68 | -0.025 | | 0.003 | 4E-17 | -0.257 | 0.130 | 0.0482 | 0.109 | 0.040 | 0.006 | 0.105 | 0.043 | 0.016 | 0.134 | 0.091 | 0.144 |
| rs9766 | 17 | 40852841 | *EZH1* | G | A | 0.54 | 0.50 | 0.98 | 0.021 | | 0.003 | 9E-13 | 0.063 | 0.104 | 0.5473 | 0.006 | 0.033 | 0.857 | 0.011 | 0.036 | 0.764 | -0.023 | 0.070 | 0.740 |
| rs4986172 | 17 | 43216281 | *ACBD4* | C | T | 0.35 | 0.43 | 0.85 | -0.034 | | 0.003 | 8E-27 | -0.364 | 0.115 | 0.0016 | 0.027 | 0.036 | 0.459 | 0.034 | 0.039 | 0.392 | -0.021 | 0.080 | 0.793 |
| rs8073371 | 17 | 46096276 | *COPZ2* | C | T | 0.22 | 0.20 | 1.00 | -0.025 | | 0.004 | 4E-12 | 0.057 | 0.129 | 0.6572 | -0.095 | 0.043 | 0.029 | -0.093 | 0.048 | 0.054 | -0.103 | 0.092 | 0.259 |
| rs318095 | 17 | 46974734 | *ATP5G1* | T | C | 0.54 | 0.52 | 0.86 | -0.024 | | 0.003 | 2E-16 | -0.211 | 0.113 | 0.0611 | -0.022 | 0.035 | 0.530 | -0.039 | 0.038 | 0.311 | 0.092 | 0.078 | 0.240 |
| rs2072153 | 17 | 47390014 | *ZNF652* | G | C | 0.31 | 0.32 | 0.99 | 0.026 | | 0.003 | 4E-16 | 0.168 | 0.114 | 0.1415 | 0.057 | 0.035 | 0.105 | 0.065 | 0.039 | 0.091 | -0.005 | 0.079 | 0.951 |
| rs4605213 | 17 | 49244747 | *NME1-NME* | G | C | 0.34 | 0.35 | 0.92 | 0.018 | | 0.003 | 8E-09 | 0.017 | 0.114 | 0.8807 | 0.000 | 0.035 | 0.998 | 0.010 | 0.039 | 0.795 | -0.071 | 0.077 | 0.358 |
| rs11867943 | 17 | 54229842 | *ANKFN1* | A | T | 0.12 | 0.11 | 0.96 | 0.027 | | 0.005 | 1E-08 | 0.275 | 0.170 | 0.1054 | 0.118 | 0.051 | 0.022 | 0.110 | 0.057 | 0.052 | 0.182 | 0.111 | 0.103 |
| rs1401795 | 17 | 54839652 | *C17orf67* | A | G | 0.49 | 0.54 | 0.56 | -0.03 | | 0.003 | 1E-23 | -0.072 | 0.138 | 0.6023 | 0.013 | 0.044 | 0.759 | -0.001 | 0.048 | 0.981 | 0.110 | 0.101 | 0.273 |
| rs2079795 | 17 | 59496649 | *C17orf82* | T | C | 0.67 | 0.67 | 0.99 | -0.045 | | 0.003 | 2E-46 | -0.414 | 0.111 | 0.0002 | -0.013 | 0.034 | 0.705 | -0.027 | 0.037 | 0.462 | 0.086 | 0.077 | 0.264 |
| rs2378870 | 17 | 59638623 | *NACA2* | T | C | 0.63 | 0.61 | 0.98 | -0.02 | | 0.003 | 4E-10 | 0.165 | 0.107 | 0.124 | 0.025 | 0.033 | 0.454 | 0.031 | 0.036 | 0.390 | -0.017 | 0.073 | 0.811 |
| rs2044124 | 17 | 61845425 | *CCDC47* | T | C | 0.94 | 0.95 | 0.91 | -0.045 | | 0.007 | 9E-12 | -0.447 | 0.245 | 0.0679 | 0.187 | 0.079 | 0.018 | 0.177 | 0.087 | 0.042 | 0.272 | 0.174 | 0.118 |
| rs2854207 | 17 | 61947107 | *CSH2* | C | G | 0.27 | 0.26 | 0.92 | 0.046 | | 0.003 | 1E-42 | 0.200 | 0.123 | 0.1051 | 0.022 | 0.037 | 0.558 | 0.026 | 0.041 | 0.522 | 0.005 | 0.085 | 0.952 |
| rs2070776 | 17 | 62007498 | *CD79B* | A | G | 0.65 | 0.66 | 1.00 | 0.042 | | 0.003 | 6E-41 | 0.382 | 0.109 | 0.0005 | -0.064 | 0.034 | 0.059 | -0.052 | 0.038 | 0.166 | -0.146 | 0.077 | 0.056 |
| rs3923086 | 17 | 63549488 | *AXIN2* | A | C | 0.60 | 0.54 | 1.00 | 0.024 | | 0.003 | 5E-13 | 0.400 | 0.106 | 0.0002 | -0.032 | 0.032 | 0.318 | -0.031 | 0.035 | 0.374 | -0.032 | 0.072 | 0.660 |
| rs2072268 | 17 | 66303352 | *ARSG* | G | A | 0.48 | 0.52 | 0.72 | -0.02 | | 0.003 | 1E-10 | -0.248 | 0.123 | 0.0426 | -0.004 | 0.037 | 0.916 | 0.000 | 0.041 | 0.996 | -0.035 | 0.083 | 0.676 |
| rs11867479 | 17 | 68090207 | *KCNJ16* | C | T | 0.35 | 0.33 | 0.97 | 0.026 | | 0.003 | 2E-16 | 0.323 | 0.112 | 0.0038 | -0.013 | 0.034 | 0.692 | -0.032 | 0.037 | 0.393 | 0.118 | 0.080 | 0.141 |
| rs10083886 | 17 | 69923355 | *SOX9* | C | T | 0.26 | 0.27 | 0.99 | 0.019 | | 0.003 | 7E-09 | 0.157 | 0.118 | 0.1861 | 0.012 | 0.036 | 0.734 | 0.022 | 0.039 | 0.578 | -0.050 | 0.085 | 0.553 |
| rs2117563 | 17 | 73368985 | *GRB2* | A | G | 0.83 | 0.81 | 0.94 | 0.024 | | 0.004 | 1E-09 | 0.442 | 0.140 | 0.0016 | 0.059 | 0.042 | 0.165 | 0.074 | 0.047 | 0.114 | -0.042 | 0.091 | 0.642 |
| rs1552173 | 17 | 76718842 | *PSCD1* | C | T | 0.54 | 0.53 | 0.60 | -0.018 | | 0.003 | 8E-10 | -0.035 | 0.137 | 0.8002 | -0.006 | 0.040 | 0.871 | -0.024 | 0.044 | 0.580 | 0.122 | 0.094 | 0.192 |
| rs1478610 | 17 | 79422252 | *BAHCC1* | G | A | 0.37 | 0.37 | 0.60 | 0.025 | | 0.004 | 9E-10 | -0.063 | 0.140 | 0.6533 | -0.031 | 0.043 | 0.473 | -0.026 | 0.047 | 0.583 | -0.071 | 0.096 | 0.457 |
| rs888403 | 18 | 2766938 | *SMCHD1* | A | G | 0.36 | 0.30 | 0.54 | 0.019 | | 0.003 | 9E-09 | -0.200 | 0.153 | 0.1906 | -0.002 | 0.047 | 0.963 | -0.012 | 0.052 | 0.814 | 0.069 | 0.103 | 0.502 |
| rs692964 | 18 | 13094132 | *CEP192* | G | A | 0.60 | 0.63 | 0.73 | -0.019 | | 0.003 | 2E-10 | -0.137 | 0.125 | 0.275 | 0.009 | 0.039 | 0.828 | -0.012 | 0.043 | 0.785 | 0.154 | 0.093 | 0.097 |
| rs14062 | 18 | 19450303 | *MIB1* | A | G | 0.67 | 0.69 | 0.87 | 0.018 | | 0.003 | 8E-09 | 0.046 | 0.121 | 0.7023 | 0.000 | 0.037 | 0.991 | -0.011 | 0.041 | 0.793 | 0.083 | 0.080 | 0.298 |
| rs4369779 | 18 | 20735408 | *CABLES1* | T | C | 0.79 | 0.81 | 0.96 | 0.056 | | 0.004 | 2E-53 | 0.486 | 0.135 | 0.0003 | -0.026 | 0.042 | 0.540 | -0.054 | 0.046 | 0.238 | 0.172 | 0.096 | 0.074 |
| rs11661645 | 18 | 45888770 | *KIAA0427* | G | A | 0.31 | 0.32 | 0.90 | 0.02 | | 0.003 | 5E-10 | 0.180 | 0.119 | 0.1285 | -0.039 | 0.036 | 0.274 | -0.026 | 0.039 | 0.500 | -0.138 | 0.085 | 0.104 |
| rs2337143 | 18 | 46482070 | *SMAD7* | A | G | 0.65 | 0.66 | 0.77 | -0.018 | | 0.003 | 3E-08 | -0.417 | 0.124 | 0.0008 | 0.025 | 0.039 | 0.519 | 0.023 | 0.042 | 0.586 | 0.041 | 0.086 | 0.632 |
| rs12458127 | 18 | 46657358 | *DYM* | C | T | 0.08 | 0.06 | 0.77 | -0.057 | | 0.006 | 1E-21 | -0.399 | 0.251 | 0.1127 | -0.152 | 0.081 | 0.062 | -0.148 | 0.090 | 0.099 | -0.182 | 0.169 | 0.281 |
| rs9967417 | 18 | 46959500 | *DYM* | G | C | 0.57 | 0.60 | 0.73 | -0.04 | | 0.003 | 2E-40 | -0.278 | 0.125 | 0.0257 | -0.011 | 0.037 | 0.767 | 0.010 | 0.041 | 0.802 | -0.160 | 0.082 | 0.053 |
| rs11152213 | 18 | 57852948 | *MC4R* | A | C | 0.25 | 0.23 | 0.99 | 0.025 | | 0.004 | 7E-13 | 0.373 | 0.123 | 0.0025 | -0.040 | 0.039 | 0.307 | -0.058 | 0.044 | 0.187 | 0.066 | 0.082 | 0.417 |
| rs8097893 | 18 | 74983055 | *GALR1* | A | G | 0.05 | 0.04 | 0.51 | -0.042 | | 0.007 | 5E-10 | 0.340 | 0.374 | 0.3623 | 0.124 | 0.110 | 0.260 | 0.142 | 0.123 | 0.250 | 0.030 | 0.220 | 0.891 |
| rs11659752 | 18 | 77222862 | *NFATC1* | T | G | 0.30 | 0.30 | 0.82 | -0.024 | | 0.003 | 6E-13 | 0.044 | 0.124 | 0.7234 | -0.003 | 0.039 | 0.937 | 0.029 | 0.042 | 0.493 | -0.235 | 0.085 | 0.006 |
| rs11880992 | 19 | 2176403 | *DOT1L* | G | A | 0.40 | 0.42 | 0.95 | 0.033 | | 0.003 | 7E-28 | 0.081 | 0.108 | 0.4525 | -0.014 | 0.034 | 0.688 | -0.019 | 0.037 | 0.612 | 0.024 | 0.077 | 0.755 |
| rs2074977 | 19 | 3434028 | *NFIC* | A | C | 0.36 | 0.35 | 1.00 | 0.029 | | 0.003 | 2E-20 | -0.118 | 0.108 | 0.2768 | 0.006 | 0.034 | 0.856 | -0.012 | 0.037 | 0.745 | 0.134 | 0.077 | 0.083 |
| rs2123731 | 19 | 4929473 | *UHRF1* | A | G | 0.27 | 0.27 | 0.70 | -0.023 | | 0.004 | 2E-11 | -0.302 | 0.138 | 0.0293 | -0.033 | 0.044 | 0.457 | -0.025 | 0.048 | 0.608 | -0.082 | 0.095 | 0.389 |
| rs891088 | 19 | 7184762 | *INSR* | A | G | 0.26 | 0.27 | 0.83 | 0.029 | | 0.003 | 7E-18 | 0.243 | 0.131 | 0.0633 | 0.015 | 0.041 | 0.719 | 0.040 | 0.045 | 0.367 | -0.172 | 0.095 | 0.070 |
| rs1346490 | 19 | 7244233 | *INSR* | A | C | 0.39 | 0.43 | 0.71 | -0.018 | | 0.003 | 1E-08 | -0.003 | 0.125 | 0.9828 | 0.043 | 0.037 | 0.245 | 0.036 | 0.041 | 0.381 | 0.087 | 0.081 | 0.285 |
| rs8102380 | 19 | 10801185 | *ILF3* | G | A | 0.69 | 0.64 | 0.61 | -0.02 | | 0.003 | 8E-11 | -0.110 | 0.137 | 0.4207 | 0.035 | 0.043 | 0.411 | 0.026 | 0.047 | 0.583 | 0.103 | 0.095 | 0.278 |
| rs7259684 | 19 | 12186611 | *LOC72974* | A | G | 0.07 | 0.09 | 0.91 | 0.035 | | 0.006 | 4E-08 | 0.158 | 0.197 | 0.4241 | -0.117 | 0.063 | 0.061 | -0.118 | 0.070 | 0.090 | -0.110 | 0.129 | 0.394 |
| rs8103068 | 19 | 17522869 | *BST2* | T | C | 0.14 | 0.15 | 0.79 | -0.031 | | 0.005 | 2E-11 | -0.302 | 0.172 | 0.0796 | -0.055 | 0.050 | 0.272 | -0.075 | 0.055 | 0.176 | 0.069 | 0.109 | 0.527 |
| rs10401193 | 19 | 19591066 | *GATAD2A* | A | G | 0.18 | 0.17 | 0.99 | -0.028 | | 0.004 | 9E-14 | 0.065 | 0.137 | 0.6347 | 0.036 | 0.043 | 0.407 | 0.024 | 0.048 | 0.622 | 0.114 | 0.094 | 0.226 |
| rs8103992 | 19 | 19665643 | *PBX4* | A | C | 0.80 | 0.78 | 0.98 | -0.029 | | 0.004 | 1E-14 | 0.069 | 0.129 | 0.593 | 0.011 | 0.040 | 0.787 | 0.046 | 0.044 | 0.299 | -0.228 | 0.088 | 0.009 |
| rs7253628 | 19 | 31047269 | *ZNF536* | A | G | 0.16 | 0.15 | 0.98 | 0.024 | | 0.004 | 2E-09 | 0.222 | 0.145 | 0.1262 | -0.041 | 0.045 | 0.356 | -0.061 | 0.049 | 0.219 | 0.089 | 0.096 | 0.357 |
| rs4802134 | 19 | 38346685 | *SIPA1L3* | A | G | 0.79 | 0.75 | 1.00 | -0.027 | | 0.004 | 3E-11 | -0.088 | 0.122 | 0.4689 | -0.018 | 0.036 | 0.612 | -0.010 | 0.040 | 0.810 | -0.074 | 0.077 | 0.336 |
| rs4803468 | 19 | 41922352 | *BCKDHA* | A | G | 0.58 | 0.59 | 0.77 | -0.03 | | 0.003 | 2E-21 | -0.057 | 0.122 | 0.6418 | -0.025 | 0.037 | 0.504 | -0.047 | 0.041 | 0.257 | 0.121 | 0.083 | 0.146 |
| rs11880124 | 19 | 42683791 | *DEDD2* | A | G | 0.09 | 0.09 | 0.94 | -0.041 | | 0.005 | 2E-14 | -0.649 | 0.189 | 0.0006 | 0.073 | 0.055 | 0.185 | 0.074 | 0.061 | 0.223 | 0.063 | 0.128 | 0.623 |
| rs2682587 | 19 | 44082429 | *XRCC1* | C | A | 0.20 | 0.18 | 1.00 | 0.023 | | 0.004 | 2E-09 | -0.057 | 0.134 | 0.672 | -0.057 | 0.042 | 0.171 | -0.057 | 0.046 | 0.211 | -0.056 | 0.091 | 0.541 |
| rs7273787 | 20 | 4098567 | *SMOX* | A | G | 0.35 | 0.30 | 0.57 | 0.022 | | 0.003 | 3E-12 | 0.027 | 0.149 | 0.8556 | 0.007 | 0.045 | 0.876 | 0.008 | 0.050 | 0.879 | 0.004 | 0.100 | 0.968 |
| rs17721822 | 20 | 6469596 | *BMP2* | G | A | 0.37 | 0.35 | 0.94 | -0.035 | | 0.003 | 3E-29 | -0.135 | 0.113 | 0.2319 | -0.038 | 0.035 | 0.281 | -0.038 | 0.038 | 0.327 | -0.036 | 0.080 | 0.654 |
| rs1884897 | 20 | 6612832 | *BMP2* | A | G | 0.64 | 0.62 | 0.80 | -0.044 | | 0.003 | 1E-48 | -0.484 | 0.120 | <.0001 | -0.051 | 0.037 | 0.175 | -0.036 | 0.041 | 0.381 | -0.163 | 0.081 | 0.043 |
| rs6080830 | 20 | 17771113 | *BANF2* | A | G | 0.44 | 0.43 | 0.68 | -0.016 | | 0.003 | 4E-08 | -0.087 | 0.127 | 0.4949 | -0.080 | 0.039 | 0.041 | -0.098 | 0.043 | 0.023 | 0.048 | 0.084 | 0.569 |
| rs7261425 | 20 | 20068635 | *C20orf26* | C | G | 0.29 | 0.29 | 0.70 | -0.021 | | 0.003 | 2E-10 | -0.176 | 0.136 | 0.1952 | -0.043 | 0.042 | 0.299 | -0.045 | 0.046 | 0.330 | -0.035 | 0.094 | 0.709 |
| rs6137287 | 20 | 21180259 | *C20orf19* | C | T | 0.31 | 0.28 | 0.95 | 0.02 | | 0.003 | 4E-10 | 0.184 | 0.118 | 0.12 | 0.041 | 0.036 | 0.265 | 0.049 | 0.040 | 0.224 | -0.019 | 0.085 | 0.821 |
| rs1074683 | 20 | 32304653 | *PXMP4* | C | G | 0.24 | 0.24 | 0.97 | -0.044 | | 0.003 | 8E-38 | 0.010 | 0.123 | 0.9379 | 0.036 | 0.037 | 0.337 | 0.027 | 0.042 | 0.516 | 0.094 | 0.078 | 0.227 |
| rs1535466 | 20 | 33718706 | *EDEM2* | G | A | 0.73 | 0.75 | 1.00 | -0.027 | | 0.003 | 3E-16 | -0.285 | 0.118 | 0.0161 | -0.063 | 0.038 | 0.094 | -0.068 | 0.042 | 0.104 | -0.036 | 0.079 | 0.643 |
| rs143384 | 20 | 34025756 | *GDF5* | A | G | 0.42 | 0.43 | 0.86 | 0.075 | | 0.003 | 1E-121 | 0.387 | 0.112 | 0.0006 | 0.052 | 0.035 | 0.129 | 0.052 | 0.038 | 0.171 | 0.053 | 0.078 | 0.497 |
| rs2425163 | 20 | 34432670 | *PHF20* | A | G | 0.18 | 0.19 | 0.98 | 0.058 | | 0.004 | 3E-52 | 0.307 | 0.131 | 0.0193 | 0.048 | 0.041 | 0.247 | 0.062 | 0.045 | 0.170 | -0.043 | 0.092 | 0.641 |
| rs4812586 | 20 | 35544673 | *SAMHD1* | A | G | 0.16 | 0.14 | 1.00 | -0.03 | | 0.004 | 9E-14 | 0.106 | 0.151 | 0.4842 | 0.018 | 0.046 | 0.700 | 0.033 | 0.051 | 0.519 | -0.075 | 0.099 | 0.446 |
| rs2224538 | 20 | 38552078 | *MAFB* | T | C | 0.35 | 0.36 | 0.87 | -0.017 | | 0.003 | 2E-08 | -0.028 | 0.116 | 0.8106 | -0.039 | 0.037 | 0.298 | -0.064 | 0.041 | 0.119 | 0.121 | 0.078 | 0.122 |
| rs17450430 | 20 | 47772264 | *STAU1* | A | T | 0.24 | 0.25 | 0.67 | 0.035 | | 0.003 | 2E-24 | 0.211 | 0.146 | 0.1492 | -0.008 | 0.045 | 0.853 | -0.010 | 0.050 | 0.840 | 0.003 | 0.100 | 0.980 |
| rs6020202 | 20 | 48634821 | *SNAI1* | G | A | 0.22 | 0.24 | 1.00 | -0.022 | | 0.004 | 8E-10 | 0.025 | 0.124 | 0.8414 | -0.017 | 0.038 | 0.651 | -0.010 | 0.041 | 0.817 | -0.067 | 0.085 | 0.428 |
| rs1326023 | 20 | 54842378 | *MC3R* | A | G | 0.70 | 0.69 | 1.00 | -0.024 | | 0.003 | 1E-13 | 0.115 | 0.114 | 0.3154 | -0.027 | 0.036 | 0.454 | -0.030 | 0.039 | 0.445 | -0.006 | 0.077 | 0.937 |
| rs2057291 | 20 | 57472043 | *GNAS* | A | G | 0.66 | 0.66 | 0.54 | -0.02 | | 0.003 | 2E-10 | 0.134 | 0.150 | 0.3703 | 0.007 | 0.044 | 0.872 | 0.006 | 0.049 | 0.904 | 0.019 | 0.104 | 0.852 |
| rs3026499 | 20 | 57948773 | *EDN3* | G | A | 0.33 | 0.32 | 0.59 | -0.026 | | 0.005 | 5E-08 | -0.157 | 0.146 | 0.2832 | -0.056 | 0.045 | 0.209 | -0.066 | 0.049 | 0.179 | 0.005 | 0.098 | 0.963 |
| rs6061231 | 20 | 60956917 | *RPS21* | C | A | 0.28 | 0.28 | 1.00 | -0.021 | | 0.003 | 1E-10 | 0.189 | 0.116 | 0.1029 | 0.014 | 0.037 | 0.695 | 0.021 | 0.041 | 0.608 | -0.026 | 0.074 | 0.730 |
| rs2829941 | 21 | 27208935 | *APP* | G | T | 0.61 | 0.59 | 0.90 | 0.017 | | 0.003 | 3E-08 | 0.113 | 0.112 | 0.3141 | -0.006 | 0.035 | 0.860 | -0.027 | 0.038 | 0.487 | 0.135 | 0.073 | 0.067 |
| rs2834442 | 21 | 35690786 | *KCNE2* | T | A | 0.64 | 0.66 | 0.98 | 0.024 | | 0.003 | 4E-15 | 0.231 | 0.112 | 0.039 | 0.019 | 0.034 | 0.565 | 0.015 | 0.037 | 0.692 | 0.049 | 0.078 | 0.531 |
| rs2211866 | 21 | 39688107 | *KCNJ15* | A | G | 0.59 | 0.59 | 0.96 | -0.022 | | 0.003 | 4E-13 | -0.077 | 0.108 | 0.4755 | -0.015 | 0.033 | 0.659 | -0.013 | 0.037 | 0.728 | -0.029 | 0.073 | 0.695 |
| rs9977276 | 21 | 47436327 | *COL6A1* | T | G | 0.78 | 0.79 | 1.00 | 0.022 | | 0.004 | 3E-10 | -0.113 | 0.128 | 0.3756 | -0.007 | 0.039 | 0.858 | 0.007 | 0.042 | 0.878 | -0.109 | 0.083 | 0.190 |
| rs7284476 | 22 | 38129332 | *TRIOBP* | G | A | 0.43 | 0.41 | 0.76 | 0.018 | | 0.003 | 6E-09 | 0.067 | 0.122 | 0.5793 | 0.056 | 0.038 | 0.143 | 0.061 | 0.042 | 0.144 | 0.017 | 0.086 | 0.842 |
| rs5757318 | 22 | 39275656 | *CBX6* | A | T | 0.15 | 0.15 | 0.70 | 0.029 | | 0.005 | 2E-09 | 0.150 | 0.173 | 0.3846 | -0.024 | 0.055 | 0.670 | -0.039 | 0.061 | 0.525 | 0.075 | 0.118 | 0.528 |
| rs738288 | 22 | 39907661 | *SMCR7L* | G | A | 0.53 | 0.57 | 0.85 | -0.02 | | 0.003 | 6E-11 | -0.030 | 0.116 | 0.7953 | 0.039 | 0.036 | 0.285 | 0.050 | 0.040 | 0.209 | -0.044 | 0.082 | 0.587 |
|  |  |  |  |  |  |  |  |  |  | |  |  |  |  |  |  |  |  |  |  |  |  |  |  |
| CIMBA: The Consortium of Investigators of Modifiers of *BRCA1/2*; GWAS: genome-wide association studies | | | | | | | |  |  | |  |  |  |  |  |  |  |  |  |  |  |  |  |  |
| **Notes:** |  |  |  |  |  |  |  |  |  | |  |  |  |  |  |  |  |  |  |  |  |  |  |  |
| [1] Imputation quality of 1 indicates genotyped SNPs. | | | |  |  |  |  |  |  | |  |  |  |  |  |  |  |  |  |  |  |  |  |  |
| [2] Effect estimate after height standardization. | | | |  |  |  |  |  |  | |  |  |  |  |  |  |  |  |  |  |  |  |  |  |
| [3] *P*-values were calculated using student's *t*-test. All *P*-values are two-sided. | | | | | |  |  |  |  | |  |  |  |  |  |  |  |  |  |  |  |  |  |  |
| [4] Association with ovarian cancer were estamated using weighted Cox models that adjusted for principal components, birth cohort, menopausal status, country of enrollment and mutation status. | | | | | | | | | | | | | |  |  |  |  |  |  |  |  |  |  |  |
| [5] *P*-values were calculated using chi-squared test. All *P*-values are two-sided. | | | | | |  |  |  | |  |  |  |  |  |  |  |  |  |  |  |  |  |  |  |

| **Supplementary Table 2.** List of 93 BMI-associated genetic variants and their associations with BMI in prior published GWAS and in CIMBA, along with effect on ovarian cancer risk in CIMBA | | | | | | | | | | | | | | | | | | | | | | | |
| --- | --- | --- | --- | --- | --- | --- | --- | --- | --- | --- | --- | --- | --- | --- | --- | --- | --- | --- | --- | --- | --- | --- | --- |
| Rsid # | Chromosome | Position | Nearest gene | Reference allele | Effect allele | Effect allele frequency in published GWAS | Effect allele frequency in CIMBA | Imputation quality^1^ | Published association with BMI | | | Association with BMI in CIMBA | | | Association with ovarian cancer in CIMBA | | | Association with ovarian cancer in *BRCA1*carriers | | | Association with ovarian cancer in *BRCA2*carriers | | |
|  |  |  |  |  |  |  |  |  | Beta^2^ | SE | *P*-value^3^ | Beta, Kg/m^2^ | SE | *P*-value^3^ | Log Hazard Ratio^4^ | SE | *P*-value^5^ | Log Hazard Ratio^4^ | SE | *P*-value^5^ | Log Hazard Ratio^4^ | SE | *P*-value^5^ |
| rs977747 | 1 | 47684677 | *TAL1* | T | G | 0.61 | 0.61 | 0.97 | -0.017 | 0.003 | 8.7E-08 | -0.164 | 0.085 | 0.0544 | 0.005 | 0.033 | 0.8834 | 0.000 | 0.036 | 0.9948 | 0.036 | 0.071 | 0.6140 |
| rs657452 | 1 | 49589847 | *AGBL4* | A | G | 0.61 | 0.62 | 0.93 | -0.023 | 0.003 | 5.5E-13 | -0.108 | 0.087 | 0.214 | -0.047 | 0.033 | 0.1596 | -0.046 | 0.037 | 0.2092 | -0.053 | 0.073 | 0.4694 |
| rs11583200 | 1 | 50559820 | *ELAVL4* | C | T | 0.60 | 0.61 | 0.88 | -0.018 | 0.003 | 1.5E-08 | -0.167 | 0.090 | 0.0622 | -0.024 | 0.035 | 0.4898 | -0.005 | 0.039 | 0.9060 | -0.162 | 0.074 | 0.0275 |
| rs3101336 | 1 | 72751185 | *NEGR1* | T | C | 0.61 | 0.64 | 1.00 | 0.033 | 0.003 | 2.7E-26 | 0.141 | 0.085 | 0.0972 | -0.025 | 0.033 | 0.4576 | -0.042 | 0.037 | 0.2464 | 0.100 | 0.077 | 0.1982 |
| rs12566985 | 1 | 75002193 | *FPGT* | G | A | 0.55 | 0.57 | 0.99 | -0.024 | 0.003 | 3.3E-15 | -0.272 | 0.082 | 0.0009 | -0.053 | 0.032 | 0.0946 | -0.075 | 0.035 | 0.0329 | 0.085 | 0.067 | 0.2055 |
| rs12401738 | 1 | 78446761 | *FUBP1* | G | A | 0.35 | 0.33 | 0.81 | 0.021 | 0.003 | 1.2E-10 | 0.106 | 0.096 | 0.2704 | 0.038 | 0.039 | 0.3234 | 0.060 | 0.042 | 0.1597 | -0.115 | 0.089 | 0.1964 |
| rs11165643 | 1 | 96924097 | *PTBP2* | C | T | 0.58 | 0.59 | 0.68 | 0.022 | 0.003 | 2.1E-12 | 0.172 | 0.101 | 0.0905 | 0.004 | 0.040 | 0.9221 | 0.021 | 0.044 | 0.6390 | -0.106 | 0.083 | 0.2023 |
| rs17024393 | 1 | 110154688 | *GNAT2* | T | C | 0.04 | 0.03 | 0.98 | 0.066 | 0.009 | 7.0E-14 | 0.456 | 0.253 | 0.0714 | -0.146 | 0.093 | 0.1181 | -0.171 | 0.101 | 0.0914 | 0.048 | 0.229 | 0.8323 |
| rs543874 | 1 | 177889480 | *SEC16B* | A | G | 0.19 | 0.18 | 1.00 | 0.048 | 0.004 | 2.6E-35 | 0.286 | 0.104 | 0.006 | 0.046 | 0.042 | 0.2717 | 0.050 | 0.046 | 0.2810 | 0.028 | 0.089 | 0.7563 |
| rs2820292 | 1 | 201784287 | *NAV1* | A | C | 0.56 | 0.54 | 0.94 | 0.020 | 0.003 | 1.8E-10 | 0.281 | 0.086 | 0.001 | -0.017 | 0.033 | 0.6073 | -0.015 | 0.036 | 0.6704 | -0.027 | 0.075 | 0.7189 |
| rs13021737 | 2 | 632348 | *TMEM18* | A | G | 0.83 | 0.81 | 0.99 | 0.060 | 0.004 | 1.1E-50 | 0.479 | 0.106 | <.0001 | 0.020 | 0.042 | 0.6332 | 0.026 | 0.046 | 0.5688 | -0.027 | 0.091 | 0.7695 |
| rs10182181 | 2 | 25150296 | *ADCY3* | A | G | 0.46 | 0.45 | 0.64 | 0.031 | 0.003 | 8.8E-24 | 0.191 | 0.104 | 0.067 | 0.013 | 0.041 | 0.7565 | 0.031 | 0.046 | 0.4987 | -0.105 | 0.094 | 0.2669 |
| rs11126666 | 2 | 26928811 | *KCNK3* | G | A | 0.28 | 0.25 | 0.54 | 0.021 | 0.003 | 1.3E-09 | -0.033 | 0.130 | 0.8018 | -0.001 | 0.050 | 0.9775 | -0.004 | 0.056 | 0.9366 | 0.014 | 0.111 | 0.9001 |
| rs1016287 | 2 | 59305625 | *LINC011* | T | C | 0.71 | 0.70 | 0.93 | -0.023 | 0.003 | 2.3E-11 | -0.105 | 0.092 | 0.2527 | 0.028 | 0.037 | 0.4436 | 0.033 | 0.040 | 0.4134 | -0.010 | 0.081 | 0.9066 |
| rs11688816 | 2 | 63053048 | *EHBP1* | G | A | 0.48 | 0.50 | 1.00 | -0.017 | 0.003 | 1.9E-08 | 0.040 | 0.082 | 0.6282 | 0.009 | 0.033 | 0.7753 | 0.011 | 0.036 | 0.7513 | -0.008 | 0.072 | 0.9121 |
| rs2121279 | 2 | 143043285 | *LRP1B* | C | T | 0.15 | 0.12 | 0.83 | 0.024 | 0.004 | 2.3E-08 | -0.102 | 0.137 | 0.4584 | -0.018 | 0.053 | 0.7381 | -0.029 | 0.059 | 0.6225 | 0.051 | 0.115 | 0.6543 |
| rs1460676 | 2 | 164567689 | *FIGN* | T | C | 0.17 | 0.17 | 1.00 | 0.020 | 0.004 | 9.0E-07 | 0.018 | 0.112 | 0.8689 | 0.080 | 0.041 | 0.0502 | 0.104 | 0.045 | 0.0201 | -0.092 | 0.097 | 0.3433 |
| rs1528435 | 2 | 181550962 | *UBE2E3* | C | T | 0.63 | 0.62 | 0.88 | 0.018 | 0.003 | 1.2E-08 | 0.111 | 0.089 | 0.2112 | 0.030 | 0.036 | 0.4070 | 0.021 | 0.040 | 0.5925 | 0.083 | 0.074 | 0.2658 |
| rs17203016 | 2 | 208255518 | *CREB1* | A | G | 0.20 | 0.18 | 0.90 | 0.021 | 0.004 | 8.2E-08 | -0.236 | 0.112 | 0.0352 | 0.064 | 0.044 | 0.1464 | 0.070 | 0.048 | 0.1450 | 0.014 | 0.096 | 0.8874 |
| rs7599312 | 2 | 213413231 | *ERBB4* | G | A | 0.28 | 0.27 | 0.88 | -0.022 | 0.003 | 1.2E-10 | 0.092 | 0.098 | 0.3483 | 0.052 | 0.039 | 0.1736 | 0.048 | 0.042 | 0.2630 | 0.078 | 0.085 | 0.3558 |
| rs492400 | 2 | 219349752 | *USP37* | C | T | 0.58 | 0.59 | 0.86 | -0.016 | 0.003 | 4.2E-07 | -0.216 | 0.090 | 0.0168 | -0.004 | 0.036 | 0.9139 | 0.000 | 0.039 | 0.9940 | -0.035 | 0.082 | 0.6667 |
| rs2176040 | 2 | 227092802 | *LOC6467* | A | G | 0.63 | 0.64 | 1.00 | -0.014 | 0.003 | 6.1E-06 | 0.003 | 0.086 | 0.9706 | 0.024 | 0.034 | 0.4749 | 0.025 | 0.037 | 0.5054 | 0.023 | 0.076 | 0.7617 |
| rs6804842 | 3 | 25106437 | *RARB* | A | G | 0.57 | 0.58 | 0.58 | 0.019 | 0.003 | 2.5E-09 | -0.050 | 0.109 | 0.6453 | 0.087 | 0.044 | 0.0461 | 0.099 | 0.048 | 0.0398 | 0.004 | 0.090 | 0.9627 |
| rs2365389 | 3 | 61236462 | *FHIT* | C | T | 0.42 | 0.41 | 0.96 | -0.020 | 0.003 | 1.6E-10 | -0.101 | 0.085 | 0.2358 | 0.022 | 0.033 | 0.5041 | 0.015 | 0.037 | 0.6915 | 0.076 | 0.072 | 0.2916 |
| rs3849570 | 3 | 81792112 | *GBE1* | C | A | 0.36 | 0.30 | 0.84 | 0.019 | 0.003 | 2.6E-08 | 0.117 | 0.097 | 0.2271 | -0.039 | 0.038 | 0.3032 | -0.060 | 0.042 | 0.1533 | 0.109 | 0.086 | 0.2055 |
| rs13078960 | 3 | 85807590 | *CADM2* | T | G | 0.20 | 0.20 | 0.91 | 0.030 | 0.004 | 1.7E-14 | 0.071 | 0.107 | 0.5102 | -0.079 | 0.042 | 0.0585 | -0.069 | 0.046 | 0.1302 | -0.136 | 0.096 | 0.1552 |
| rs16851483 | 3 | 141275436 | *RASA2* | G | T | 0.07 | 0.07 | 1.00 | 0.048 | 0.008 | 3.6E-10 | -0.141 | 0.163 | 0.3871 | -0.203 | 0.068 | 0.0030 | -0.204 | 0.075 | 0.0068 | -0.188 | 0.148 | 0.2033 |
| rs1516725 | 3 | 185824004 | *ETV5* | T | C | 0.87 | 0.86 | 0.88 | 0.045 | 0.005 | 1.9E-22 | -0.071 | 0.127 | 0.5764 | 0.043 | 0.050 | 0.3829 | 0.053 | 0.055 | 0.3376 | -0.020 | 0.108 | 0.8511 |
| rs10938397 | 4 | 45182527 | *GNPDA2* | A | G | 0.43 | 0.43 | 1.00 | 0.040 | 0.003 | 3.2E-38 | 0.302 | 0.083 | 0.0003 | 0.004 | 0.033 | 0.8913 | 0.016 | 0.036 | 0.6491 | -0.076 | 0.073 | 0.2982 |
| rs17001654 | 4 | 77129568 | *SCARB2* | C | G | 0.15 | 0.17 | 0.75 | 0.031 | 0.005 | 7.8E-09 | 0.044 | 0.128 | 0.7285 | 0.019 | 0.048 | 0.6883 | 0.021 | 0.053 | 0.6881 | 0.006 | 0.106 | 0.9567 |
| rs13107325 | 4 | 103188709 | *SLC39A8* | C | T | 0.07 | 0.09 | 0.76 | 0.048 | 0.007 | 1.8E-12 | 0.163 | 0.171 | 0.3384 | 0.035 | 0.067 | 0.6061 | 0.031 | 0.074 | 0.6751 | 0.056 | 0.150 | 0.7092 |
| rs11727676 | 4 | 145659064 | *HHIP* | T | C | 0.09 | 0.09 | 0.68 | -0.036 | 0.006 | 2.6E-08 | -0.055 | 0.172 | 0.751 | 0.060 | 0.064 | 0.3526 | 0.059 | 0.070 | 0.4042 | 0.066 | 0.159 | 0.6756 |
| rs2112347 | 5 | 75015242 | *POC5* | T | G | 0.37 | 0.37 | 0.99 | -0.026 | 0.003 | 6.2E-17 | -0.239 | 0.085 | 0.0052 | -0.008 | 0.033 | 0.8196 | 0.013 | 0.037 | 0.7139 | -0.158 | 0.076 | 0.0369 |
| rs7715256 | 5 | 153537893 | *GALNT10* | G | T | 0.58 | 0.56 | 0.99 | -0.016 | 0.003 | 1.7E-07 | -0.167 | 0.083 | 0.0449 | -0.049 | 0.033 | 0.1373 | -0.049 | 0.036 | 0.1770 | -0.051 | 0.071 | 0.4782 |
| rs205262 | 6 | 34563164 | *C6orf10* | A | G | 0.27 | 0.28 | 1.00 | 0.022 | 0.004 | 1.8E-10 | 0.185 | 0.091 | 0.0419 | -0.015 | 0.036 | 0.6817 | -0.017 | 0.039 | 0.6608 | 0.003 | 0.078 | 0.9739 |
| rs2033529 | 6 | 40348653 | *TDRG1* | A | G | 0.29 | 0.30 | 0.63 | 0.019 | 0.003 | 1.4E-08 | -0.015 | 0.113 | 0.8912 | -0.040 | 0.045 | 0.3724 | -0.034 | 0.049 | 0.4949 | -0.077 | 0.098 | 0.4344 |
| rs2207139 | 6 | 50845490 | *TFAP2B* | A | G | 0.18 | 0.16 | 0.99 | 0.045 | 0.004 | 4.1E-29 | 0.170 | 0.110 | 0.1224 | 0.120 | 0.043 | 0.0050 | 0.146 | 0.047 | 0.0019 | -0.057 | 0.094 | 0.5439 |
| rs9400239 | 6 | 108977663 | *FOXO3* | T | C | 0.69 | 0.70 | 0.97 | 0.019 | 0.003 | 1.6E-08 | 0.105 | 0.091 | 0.2458 | 0.016 | 0.035 | 0.6492 | 0.024 | 0.039 | 0.5304 | -0.036 | 0.075 | 0.6292 |
| rs9374842 | 6 | 120185665 | *LOC2857* | C | T | 0.75 | 0.77 | 0.89 | 0.019 | 0.004 | 9.7E-08 | 0.055 | 0.105 | 0.6025 | 0.021 | 0.040 | 0.5949 | 0.018 | 0.044 | 0.6728 | 0.040 | 0.089 | 0.6523 |
| rs13191362 | 6 | 163033350 | *PARK2* | A | G | 0.12 | 0.12 | 0.91 | -0.028 | 0.005 | 7.3E-09 | -0.026 | 0.131 | 0.8453 | 0.022 | 0.052 | 0.6650 | 0.054 | 0.057 | 0.3383 | -0.192 | 0.117 | 0.1001 |
| rs1167827 | 7 | 75163169 | *HIP1* | A | G | 0.55 | 0.58 | 1.00 | 0.020 | 0.003 | 6.3E-10 | -0.077 | 0.084 | 0.3553 | -0.010 | 0.032 | 0.7688 | 0.006 | 0.036 | 0.8681 | -0.110 | 0.072 | 0.1268 |
| rs9641123 | 7 | 93197732 | *CALCR* | G | C | 0.43 | 0.41 | 1.00 | 0.019 | 0.004 | 5.0E-07 | 0.266 | 0.084 | 0.0016 | 0.014 | 0.031 | 0.6584 | 0.003 | 0.035 | 0.9228 | 0.082 | 0.069 | 0.2323 |
| rs6465468 | 7 | 95169514 | *ASB4* | G | T | 0.30 | 0.26 | 0.65 | 0.017 | 0.004 | 2.3E-06 | 0.031 | 0.117 | 0.7881 | -0.074 | 0.046 | 0.1082 | -0.076 | 0.051 | 0.1339 | -0.056 | 0.099 | 0.5758 |
| rs17405819 | 8 | 76806584 | *HNF4G* | T | C | 0.30 | 0.31 | 0.97 | -0.022 | 0.003 | 2.1E-11 | -0.203 | 0.089 | 0.023 | -0.010 | 0.035 | 0.7822 | -0.020 | 0.039 | 0.6132 | 0.054 | 0.074 | 0.4689 |
| rs16907751 | 8 | 81375457 | *ZBTB10* | C | T | 0.08 | 0.12 | 0.79 | -0.035 | 0.007 | 1.3E-07 | -0.187 | 0.145 | 0.1977 | -0.027 | 0.056 | 0.6278 | -0.052 | 0.063 | 0.4017 | 0.131 | 0.118 | 0.2670 |
| rs2033732 | 8 | 85079709 | *RALYL* | T | C | 0.75 | 0.75 | 0.72 | 0.019 | 0.004 | 4.9E-08 | 0.068 | 0.112 | 0.5437 | -0.088 | 0.042 | 0.0369 | -0.047 | 0.047 | 0.3162 | -0.345 | 0.088 | <.0001 |
| rs4740619 | 9 | 15634326 | *C9orf93* | T | C | 0.46 | 0.46 | 0.97 | -0.018 | 0.003 | 4.6E-09 | -0.065 | 0.083 | 0.4323 | 0.034 | 0.034 | 0.3128 | 0.032 | 0.037 | 0.3994 | 0.051 | 0.072 | 0.4843 |
| rs10968576 | 9 | 28414339 | *LINGO2* | A | G | 0.32 | 0.30 | 0.99 | 0.025 | 0.003 | 6.6E-14 | 0.098 | 0.089 | 0.2701 | 0.048 | 0.035 | 0.1739 | 0.056 | 0.039 | 0.1475 | -0.004 | 0.080 | 0.9639 |
| rs6477694 | 9 | 111932342 | *EPB41L4* | C | T | 0.63 | 0.67 | 0.97 | -0.017 | 0.003 | 2.7E-08 | -0.152 | 0.088 | 0.0851 | -0.042 | 0.034 | 0.2077 | -0.042 | 0.037 | 0.2565 | -0.045 | 0.071 | 0.5226 |
| rs1928295 | 9 | 120378483 | *TLR4* | T | C | 0.45 | 0.45 | 0.91 | -0.019 | 0.003 | 7.9E-10 | -0.139 | 0.087 | 0.1099 | -0.047 | 0.034 | 0.1612 | -0.041 | 0.037 | 0.2647 | -0.084 | 0.077 | 0.2744 |
| rs10733682 | 9 | 129460914 | *LMX1B* | A | G | 0.52 | 0.51 | 0.83 | -0.017 | 0.003 | 1.8E-08 | -0.070 | 0.091 | 0.4389 | 0.026 | 0.036 | 0.4748 | 0.029 | 0.039 | 0.4533 | 0.002 | 0.080 | 0.9764 |
| rs7899106 | 10 | 87410904 | *GRID1* | A | G | 0.05 | 0.05 | 0.95 | 0.040 | 0.007 | 3.0E-08 | 0.084 | 0.191 | 0.6602 | 0.097 | 0.075 | 0.1964 | 0.126 | 0.081 | 0.1228 | -0.132 | 0.176 | 0.4525 |
| rs17094222 | 10 | 102395440 | *HIF1AN* | T | C | 0.21 | 0.22 | 0.74 | 0.025 | 0.004 | 5.9E-11 | 0.140 | 0.114 | 0.219 | -0.019 | 0.044 | 0.6750 | -0.020 | 0.049 | 0.6898 | -0.012 | 0.097 | 0.8987 |
| rs11191560 | 10 | 104869038 | *NT5C2* | T | C | 0.09 | 0.09 | 0.86 | 0.031 | 0.005 | 8.5E-09 | 0.029 | 0.160 | 0.8581 | -0.113 | 0.061 | 0.0636 | -0.123 | 0.067 | 0.0655 | -0.051 | 0.136 | 0.7070 |
| rs7903146 | 10 | 114758349 | *TCF7L2* | C | T | 0.29 | 0.30 | 1.00 | -0.023 | 0.003 | 1.1E-11 | -0.151 | 0.089 | 0.0896 | -0.061 | 0.034 | 0.0746 | -0.061 | 0.038 | 0.1055 | -0.061 | 0.074 | 0.4080 |
| rs4256980 | 11 | 8673939 | *TRIM66* | C | G | 0.65 | 0.64 | 1.00 | 0.021 | 0.003 | 2.9E-11 | 0.093 | 0.086 | 0.2804 | -0.041 | 0.033 | 0.2173 | -0.038 | 0.036 | 0.2930 | -0.060 | 0.074 | 0.4214 |
| rs11030104 | 11 | 27684517 | *BDNF* | A | G | 0.21 | 0.23 | 0.99 | -0.041 | 0.004 | 5.6E-28 | -0.369 | 0.098 | 0.0002 | 0.039 | 0.039 | 0.3073 | 0.068 | 0.042 | 0.1101 | -0.147 | 0.085 | 0.0850 |
| rs2176598 | 11 | 43864278 | *HSD17B1* | T | C | 0.75 | 0.74 | 0.98 | -0.020 | 0.004 | 3.0E-08 | 0.049 | 0.095 | 0.6044 | 0.013 | 0.037 | 0.7153 | 0.028 | 0.041 | 0.4975 | -0.081 | 0.075 | 0.2791 |
| rs3817334 | 11 | 47650993 | *MTCH2* | C | T | 0.41 | 0.43 | 0.75 | 0.026 | 0.003 | 5.2E-17 | 0.170 | 0.097 | 0.0781 | 0.034 | 0.037 | 0.3547 | 0.036 | 0.040 | 0.3671 | 0.019 | 0.084 | 0.8219 |
| rs12286929 | 11 | 115022404 | *CADM1* | A | G | 0.52 | 0.51 | 0.72 | 0.022 | 0.003 | 1.3E-12 | -0.001 | 0.097 | 0.9879 | -0.056 | 0.038 | 0.1402 | -0.055 | 0.042 | 0.1948 | -0.069 | 0.081 | 0.3895 |
| rs7138803 | 12 | 50247468 | *BCDIN3D* | G | A | 0.38 | 0.39 | 0.95 | 0.032 | 0.003 | 8.2E-24 | 0.079 | 0.086 | 0.3581 | 0.027 | 0.033 | 0.4177 | 0.036 | 0.037 | 0.3301 | -0.028 | 0.073 | 0.7044 |
| rs11057405 | 12 | 122781897 | *CLIP1* | G | A | 0.10 | 0.10 | 0.69 | -0.031 | 0.006 | 2.0E-08 | -0.283 | 0.160 | 0.0765 | 0.084 | 0.068 | 0.2179 | 0.086 | 0.075 | 0.2522 | 0.076 | 0.148 | 0.6088 |
| rs12429545 | 13 | 54102206 | *OLFM4* | G | A | 0.13 | 0.14 | 0.52 | 0.033 | 0.005 | 1.1E-12 | 0.282 | 0.161 | 0.0794 | 0.084 | 0.064 | 0.1902 | 0.077 | 0.071 | 0.2794 | 0.134 | 0.144 | 0.3488 |
| rs9540493 | 13 | 66205704 | *MIR548X* | A | G | 0.54 | 0.55 | 0.62 | -0.017 | 0.003 | 1.4E-07 | 0.029 | 0.104 | 0.7778 | -0.045 | 0.041 | 0.2635 | -0.058 | 0.045 | 0.1951 | 0.046 | 0.085 | 0.5824 |
| rs1441264 | 13 | 79580919 | *MIR548A* | G | A | 0.61 | 0.64 | 0.55 | 0.018 | 0.003 | 6.0E-08 | 0.114 | 0.115 | 0.3219 | 0.001 | 0.044 | 0.9736 | 0.006 | 0.049 | 0.9034 | -0.030 | 0.093 | 0.7455 |
| rs10132280 | 14 | 25928179 | *STXBP6* | C | A | 0.32 | 0.32 | 0.88 | -0.023 | 0.003 | 1.1E-11 | -0.262 | 0.095 | 0.0058 | -0.051 | 0.037 | 0.1726 | -0.066 | 0.041 | 0.1104 | 0.049 | 0.080 | 0.5403 |
| rs12885454 | 14 | 29736838 | *PRKD1* | C | A | 0.36 | 0.34 | 0.99 | -0.021 | 0.003 | 1.9E-10 | -0.129 | 0.086 | 0.1356 | -0.043 | 0.034 | 0.1983 | -0.055 | 0.037 | 0.1390 | 0.039 | 0.076 | 0.6033 |
| rs7141420 | 14 | 79899454 | *NRXN3* | C | T | 0.53 | 0.50 | 0.64 | 0.023 | 0.003 | 1.2E-14 | -0.014 | 0.102 | 0.8951 | 0.006 | 0.040 | 0.8734 | 0.004 | 0.044 | 0.9332 | 0.029 | 0.095 | 0.7596 |
| rs3736485 | 15 | 51748610 | *DMXL2* | A | G | 0.55 | 0.51 | 0.89 | -0.018 | 0.003 | 7.4E-09 | -0.092 | 0.087 | 0.2915 | 0.009 | 0.035 | 0.7923 | 0.004 | 0.039 | 0.9190 | 0.043 | 0.072 | 0.5526 |
| rs16951275 | 15 | 68077168 | *MAP2K5* | T | C | 0.22 | 0.24 | 1.00 | -0.031 | 0.004 | 1.9E-17 | -0.265 | 0.096 | 0.0055 | 0.013 | 0.037 | 0.7218 | 0.032 | 0.040 | 0.4236 | -0.130 | 0.084 | 0.1245 |
| rs7164727 | 15 | 73093991 | *LOC1002* | C | T | 0.69 | 0.67 | 0.91 | 0.018 | 0.003 | 6.8E-08 | -0.004 | 0.092 | 0.9632 | 0.033 | 0.035 | 0.3578 | 0.008 | 0.039 | 0.8384 | 0.198 | 0.079 | 0.0121 |
| rs758747 | 16 | 3627358 | *NLRC3* | C | T | 0.27 | 0.29 | 1.00 | 0.023 | 0.004 | 7.5E-10 | 0.041 | 0.090 | 0.645 | 0.005 | 0.035 | 0.8935 | 0.002 | 0.038 | 0.9688 | 0.032 | 0.072 | 0.6600 |
| rs12446632 | 16 | 19935389 | *GPRC5B* | G | A | 0.13 | 0.14 | 0.98 | -0.040 | 0.005 | 1.5E-18 | -0.254 | 0.116 | 0.0282 | 0.007 | 0.046 | 0.8845 | 0.005 | 0.050 | 0.9145 | 0.008 | 0.109 | 0.9402 |
| rs2650492 | 16 | 28333411 | *SBK1* | G | A | 0.30 | 0.23 | 0.61 | 0.021 | 0.004 | 1.9E-09 | 0.235 | 0.126 | 0.0623 | -0.053 | 0.050 | 0.2954 | -0.055 | 0.055 | 0.3215 | -0.028 | 0.109 | 0.7970 |
| rs3888190 | 16 | 28889486 | *ATP2A1* | C | A | 0.40 | 0.36 | 1.00 | 0.031 | 0.003 | 3.1E-23 | 0.160 | 0.085 | 0.0604 | -0.025 | 0.034 | 0.4748 | -0.022 | 0.038 | 0.5647 | -0.042 | 0.077 | 0.5839 |
| rs4787491 | 16 | 30015337 | *INO80E* | A | G | 0.51 | 0.54 | 0.92 | 0.016 | 0.003 | 2.2E-06 | 0.212 | 0.086 | 0.0134 | -0.022 | 0.035 | 0.5247 | -0.025 | 0.039 | 0.5115 | -0.004 | 0.075 | 0.9608 |
| rs9925964 | 16 | 31129895 | *KAT8* | A | G | 0.38 | 0.39 | 0.99 | -0.019 | 0.003 | 8.1E-10 | -0.238 | 0.084 | 0.0047 | 0.034 | 0.033 | 0.2994 | 0.035 | 0.036 | 0.3384 | 0.029 | 0.070 | 0.6797 |
| rs2080454 | 16 | 49062590 | *CBLN1* | C | A | 0.59 | 0.59 | 0.72 | -0.017 | 0.003 | 6.6E-08 | -0.011 | 0.097 | 0.9114 | 0.073 | 0.038 | 0.0568 | 0.074 | 0.042 | 0.0794 | 0.060 | 0.085 | 0.4771 |
| rs1558902 | 16 | 53803574 | *FTO* | T | A | 0.42 | 0.42 | 1.00 | 0.082 | 0.003 | 7.5E-153 | 0.492 | 0.083 | <.0001 | 0.059 | 0.031 | 0.0613 | 0.070 | 0.035 | 0.0422 | -0.021 | 0.070 | 0.7598 |
| rs9914578 | 17 | 2005136 | *SMG6* | C | G | 0.21 | 0.19 | 0.98 | 0.020 | 0.004 | 9.0E-08 | 0.136 | 0.104 | 0.1915 | 0.021 | 0.040 | 0.5872 | 0.008 | 0.044 | 0.8612 | 0.113 | 0.088 | 0.1993 |
| rs1000940 | 17 | 5283252 | *RABEP1* | A | G | 0.32 | 0.31 | 0.99 | 0.019 | 0.003 | 1.3E-08 | 0.074 | 0.088 | 0.3998 | -0.018 | 0.036 | 0.6137 | -0.010 | 0.039 | 0.7912 | -0.076 | 0.079 | 0.3371 |
| rs12940622 | 17 | 78615571 | *RPTOR* | G | A | 0.43 | 0.42 | 0.73 | -0.018 | 0.003 | 2.5E-09 | -0.040 | 0.097 | 0.6779 | -0.012 | 0.037 | 0.7392 | -0.010 | 0.041 | 0.8039 | -0.027 | 0.082 | 0.7445 |
| rs1808579 | 18 | 21104888 | *C18orf8* | C | T | 0.47 | 0.44 | 0.91 | -0.017 | 0.003 | 4.2E-08 | -0.086 | 0.087 | 0.3217 | 0.026 | 0.034 | 0.4414 | 0.040 | 0.037 | 0.2856 | -0.068 | 0.075 | 0.3650 |
| rs7239883 | 18 | 40147671 | *LOC2842* | G | A | 0.61 | 0.60 | 0.85 | -0.016 | 0.003 | 1.6E-07 | -0.098 | 0.091 | 0.284 | -0.005 | 0.036 | 0.9002 | -0.029 | 0.040 | 0.4699 | 0.163 | 0.081 | 0.0450 |
| rs7243357 | 18 | 56883319 | *GRP* | T | G | 0.19 | 0.18 | 0.94 | -0.022 | 0.004 | 3.9E-08 | -0.025 | 0.111 | 0.8186 | 0.016 | 0.043 | 0.7074 | 0.009 | 0.047 | 0.8532 | 0.067 | 0.091 | 0.4640 |
| rs6567160 | 18 | 57829135 | *MC4R* | T | C | 0.24 | 0.23 | 0.99 | 0.056 | 0.004 | 3.9E-53 | 0.408 | 0.097 | <.0001 | -0.038 | 0.040 | 0.3315 | -0.054 | 0.044 | 0.2159 | 0.057 | 0.082 | 0.4855 |
| rs17724992 | 19 | 18454825 | *PGPEP1* | A | G | 0.25 | 0.26 | 1.00 | -0.019 | 0.004 | 3.4E-08 | -0.090 | 0.093 | 0.333 | -0.057 | 0.036 | 0.1124 | -0.060 | 0.040 | 0.1308 | -0.042 | 0.078 | 0.5941 |
| rs29941 | 19 | 34309532 | *KCTD15* | A | G | 0.67 | 0.69 | 1.00 | 0.018 | 0.003 | 2.4E-08 | 0.118 | 0.089 | 0.1833 | -0.006 | 0.034 | 0.8581 | -0.002 | 0.037 | 0.9613 | -0.031 | 0.074 | 0.6744 |
| rs2075650 | 19 | 45395619 | *TOMM40* | A | G | 0.15 | 0.14 | 0.75 | -0.026 | 0.005 | 1.3E-08 | -0.080 | 0.135 | 0.5536 | 0.057 | 0.053 | 0.2845 | 0.053 | 0.058 | 0.3618 | 0.071 | 0.118 | 0.5470 |
| rs2287019 | 19 | 46202172 | *QPCTL* | C | T | 0.20 | 0.21 | 0.67 | -0.036 | 0.004 | 4.6E-18 | -0.093 | 0.122 | 0.4445 | -0.004 | 0.050 | 0.9289 | -0.029 | 0.055 | 0.6052 | 0.172 | 0.103 | 0.0935 |
| rs3810291 | 19 | 47569003 | *ZC3H4* | G | A | 0.67 | 0.67 | 1.00 | 0.028 | 0.004 | 4.8E-15 | 0.075 | 0.087 | 0.3908 | -0.013 | 0.034 | 0.7063 | -0.018 | 0.038 | 0.6384 | 0.017 | 0.073 | 0.8201 |
| rs6091540 | 20 | 51087862 | *ZFP64* | C | T | 0.28 | 0.28 | 0.64 | -0.019 | 0.004 | 8.0E-08 | 0.051 | 0.114 | 0.6513 | 0.022 | 0.044 | 0.6153 | 0.024 | 0.048 | 0.6182 | 0.016 | 0.098 | 0.8716 |
| rs2836754 | 21 | 40291740 | *ETS2* | T | C | 0.61 | 0.62 | 1.00 | 0.016 | 0.003 | 4.2E-07 | 0.051 | 0.084 | 0.542 | -0.051 | 0.033 | 0.1204 | -0.054 | 0.036 | 0.1355 | -0.032 | 0.071 | 0.6574 |
|  |  |  |  |  |  |  |  |  |  |  |  |  |  |  |  |  |  |  |  |  |  |  |  |
| CIMBA: The Consortium of Investigators of Modifiers of *BRCA1/2*; GWAS: genome-wide association studies | | | | | | | |  |  |  |  |  |  |  |  |  |  |  |  |  |  |  |  |
| **Notes:** |  |  |  |  |  |  |  |  |  |  |  |  |  |  |  |  |  |  |  |  |  |  |  |
| [1] Imputation quality of 1 indicates genotyped SNPs. | | | |  |  |  |  |  |  |  |  |  |  |  |  |  |  |  |  |  |  |  |  |
| [2] Effect estimate after BMI standardization. | | | |  |  |  |  |  |  |  |  |  |  |  |  |  |  |  |  |  |  |  |  |
| [3] *P*-values were calculated using student's *t*-test. All *P*-values are two-sided. | | | | | |  |  |  |  |  |  |  |  |  |  |  |  |  |  |  |  |  |  |
| [4] Association with ovarian cancer were estamated using weighted Cox models that adjusted for principal components, birth cohort, menopausal status, country of enrollment and mutation status. | | | | | | | | | | | | |  |  |  |  |  |  |  |  |  |  |  |
| [5] *P*-values were calculated using chi-squared test. All *P*-values are two-sided. | | | | | |  |  |  |  |  |  |  |  |  |  |  |  |  |  |  |  |  |  |

| **Supplementary Table 3.** Associations of the height genetic score (height-GS) with height and ovarian cancer risk factors | | | | | |
| --- | --- | --- | --- | --- | --- |
|  | Number of participants | Summary effect | Standard error | *P*-value | % variation explained |
|  |  |  |  |  |  |
| Measured height, cm |  |  |  |  |  |
| All participants | 7,657 | 0.993 | 0.029 | 7.00E-241 | 13.4 |
| *BRCA1* carriers | 4,502 | 1.004 | 0.037 | 3.84E-149 | 14.0 |
| *BRCA2* carriers | 3,155 | 0.980 | 0.046 | 2.48E-94 | 12.6 |
| Case participants | 784 | 0.939 | 0.087 | 1.20E-25 | 13.1 |
| Control participants | 6,873 | 1.000 | 0.031 | 2.31E-218 | 13.5 |
| Traditional risk factors |  |  |  |  |  |
| BMI, kg/m^2^ | 7,516 | -0.010 | 0.024 | 0.66 |  |
| Weight, kg | 7,569 | 0.798 | 0.064 | 2.73E-35 |  |
| Age at baseline, y | 22,588 | -0.128 | 0.032 | 4.52E-05 |  |
| Age at menarche, y | 7,459 | 0.028 | 0.007 | 7.25E-05 |  |
| Parous, yes vs no | 8,394 | -0.010 | 0.011 | 0.35 |  |
| Age at first live birth, y | 6,290 | -0.013 | 0.025 | 0.58 |  |
| Menopausal status, pre vs post | 8,445 | 0.012 | 0.009 | 0.18 |  |
| Age at menopause, y | 4,336 | -0.080 | 0.036 | 0.03 |  |
|  |  |  |  |  |  |
| **Notes:** |  |  |  |  |  |
| Regression coefficient is presented for continuous variables and natural log-scale odds ratio for binary variables, per unit increase of the weighted height genetic score. P-values were calculated from linear regression models for all variables except for parity and menopausal status (logistic regression models). | | | | | |

| **Supplementary Table 4.** Associations of the body mass index genetic score (BMI-GS) with BMI and ovarian cancer risk factors | | | | | |
| --- | --- | --- | --- | --- | --- |
|  | Number of participants | Summary effect | Standard error | *P*-value | % variation explained |
|  |  |  |  |  |  |
| Observed BMI at date of questionniare, kg/m^2^ | |  |  |  |  |
| All participants | 7,516 | 0.966 | 0.069 | 2.79E-44 | 2.6 |
| *BRCA1* carrier | 4,401 | 0.912 | 0.088 | 8.05E-25 | 2.4 |
| *BRCA2* carrier | 3,115 | 1.048 | 0.110 | 2.10E-21 | 2.9 |
| Case participants | 772 | 0.671 | 0.215 | 1.88E-03 | 1.3 |
| Control participants | 6,744 | 1.000 | 0.073 | 1.11E-42 | 2.7 |
| Premenopausal control participants | 3,253 | 0.949 | 0.101 | 1.09E-20 | 2.6 |
| Postmenopausal control participants | 3,152 | 1.052 | 0.109 | 8.10E-22 | 2.9 |
| Observed BMI in young adulthood, kg/m^2^ |  |  |  |  |  |
| All participants | 5,417 | 0.971 | 0.102 | 2.31E-21 | 1.7 |
| *BRCA1* carrier | 3,134 | 0.942 | 0.132 | 1.24E-12 | 1.6 |
| *BRCA2* carrier | 2,283 | 1.015 | 0.160 | 2.76E-10 | 1.7 |
| Case participants | 536 | 0.734 | 0.285 | 1.04E-02 | 1.2 |
| Control participants | 4,881 | 1.000 | 0.109 | 5.69E-20 | 1.7 |
| Premenopausal control participants | 2,180 | 1.171 | 0.168 | 4.18E-12 | 2.2 |
| Postmenopausal control participants | 2,365 | 0.872 | 0.153 | 1.27E-08 | 1.4 |
| Traditional risk factors |  |  |  |  |  |
| Height | 7,657 | 0.371 | 0.092 | 5.40E-05 |  |
| Age at baseline, y | 22,588 | -0.165 | 0.085 | 0.79 |  |
| Age at menarche, y | 7,459 | -0.104 | 0.020 | 3.30E-07 |  |
| Parous, yes vs no | 8,394 | 0.028 | 0.031 | 0.38 |  |
| Age at first live birth, y | 6,290 | -0.131 | 0.073 | 0.07 |  |
| Menopausal status, pre vs post | 8,445 | -0.027 | 0.026 | 0.28 |  |
| Age at menopause, y | 4,336 | -0.200 | 0.108 | 0.06 |  |
|  |  |  |  |  |  |
| **Notes:** |  |  |  |  |  |
| Regression coefficient is presented for continuous variables and natural log-scale odds ratio for binary variables, per unit increase of the weighted BMI genetic score. *P*-values were calculated from linear regression models for all variables except for parity and menopausal status (logistic regression models). | | | | | |
